# *Drosophila* as a Genetic Model for Hematopoiesis

**DOI:** 10.1534/genetics.118.300223

**Published:** 2019-02-05

**Authors:** Utpal Banerjee, Juliet R. Girard, Lauren M. Goins, Carrie M. Spratford

**Affiliations:** * Department of Molecular, Cell, and Developmental Biology, University of California, Los Angeles, California 90095; † Molecular Biology Institute, University of California, Los Angeles, California 90095; ‡ Department of Biological Chemistry, University of California, Los Angeles, California 90095; § Eli and Edythe Broad Center of Regenerative Medicine and Stem Cell Research, University of California, Los Angeles, California 90095

**Keywords:** crystal cell, *Drosophila*, FlyBook, hematopoiesis, hemocyte, innate immunity, lamellocyte, lymph gland, plasmatocyte, stress response

## Abstract

In this FlyBook chapter, we present a survey of the current literature on the development of the hematopoietic system in *Drosophila*. The *Drosophila* blood system consists entirely of cells that function in innate immunity, tissue integrity, wound healing, and various forms of stress response, and are therefore functionally similar to myeloid cells in mammals. The primary cell types are specialized for phagocytic, melanization, and encapsulation functions. As in mammalian systems, multiple sites of hematopoiesis are evident in *Drosophila* and the mechanisms involved in this process employ many of the same molecular strategies that exemplify blood development in humans. *Drosophila* blood progenitors respond to internal and external stress by coopting developmental pathways that involve both local and systemic signals. An important goal of these *Drosophila* studies is to develop the tools and mechanisms critical to further our understanding of human hematopoiesis during homeostasis and dysfunction.

## Evolution of Blood

THE study of invertebrate hemocytes, and their likely evolutionary link to mammalian blood, has a long and chequered past. It seems likely that the earliest metazoan hemocytes are analogous to mammalian blood cells, *i.e.*, they share similar functions but might have arisen through a polyphyletic system of independent evolutionary events. Homology between the two evolutionarily distant systems would imply a monophyletic pathway, and it seems very likely that some cell types evolved only once while others might have evolved independently [reviewed in Millar and Ratcliffe (1989)]. Historically, this debate is further complicated due to the variety of names that have been assigned to blood cells within the invertebrate phyla, even as they represent identical cell types [reviewed in Ratcliffe and Rowley (1981)]. This is in contrast to the well-established nomenclature for cells of the hematopoietic system in mammals [reviewed in Weissman *et al.* (2001)]. Molecular and genetic approaches are now accessible for use broadly across metazoans and such investigations will shed further light onto this important evolutionary question [reviewed in Hartenstein (2006)].

Debates over analogy and homology are not specific to the blood. For example, homology in eye development remained elusive despite clear functional and molecular similarities between them [reviewed in Gehring (1996)]. Visual transduction by invertebrate rhabdomeric-Rhodopsin (r-R) (Arendt *et al.* 2004) and vertebrate ciliary-Rhodopsin (c-R) were thought to have evolved independently, until the unexpected finding that both r-R and c-R are found in the invertebrate ragworm (Arendt *et al.* 2004). This nonmodel system study was critical to the findings that rhodopsins are specialized through evolution for photoreceptors, retinal ganglion cells, and cells that control circadian rhythms, as needed [reviewed in Ernst *et al.* (2014)].

**Figure 1:**
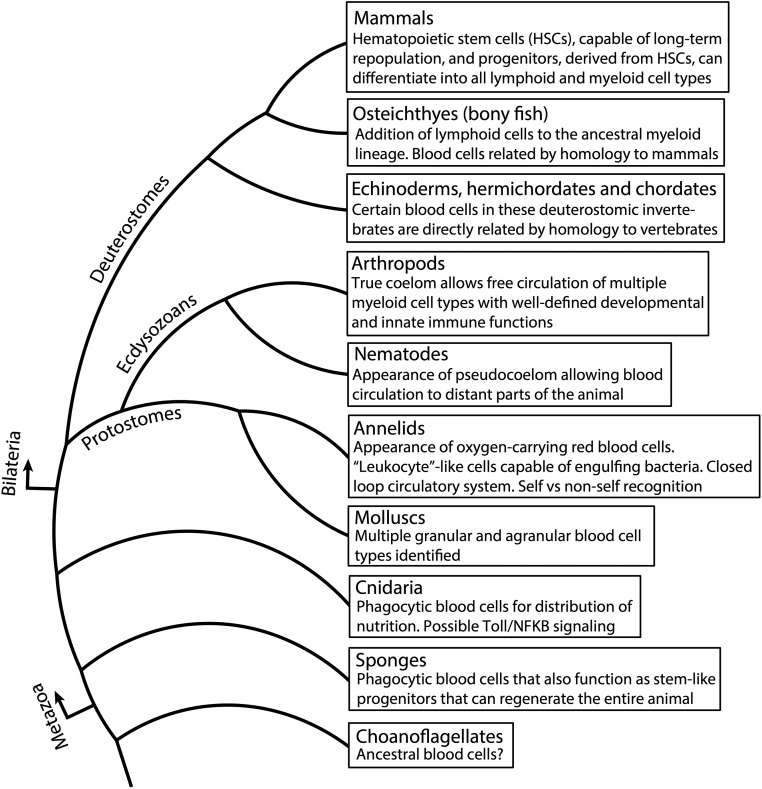
Phylogenetic tree depicting key events during the evolution of metazoan blood cells. HSCs, hematopoietic stem cells.

We can anticipate a similar scenario for the evolution of metazoan hematopoiesis (Figure 1). Blood cells likely arose in the choanoflagellate ancestors of metazoans since they are readily apparent in several species of diploblastic sponges, which lack a mesoderm. These species contain a group of cells, termed archaeocytes, that can efficiently generate all of the 10 cell types that give rise to the entire animal (De Sutter and Buscema 1977; De Sutter and Van de Vyver 1977; Simpson 1984). The rest of the cell types lack this regenerative potential and, thus, the archaeocytes are *bona fide* stem cells that are maintained through the life of the animal. Interestingly, these circulating archaeocytes are phagocytic, not unlike those seen in more evolved animals, such as the mammalian macrophages and microglia. The primary function of these phagocytic cells is to gather nutrition through engulfment and deliver this to the rest of the cells of the animal. Phagocytes are considered to be the only blood cell type that has been maintained throughout evolution in a monophyletic manner, radiating out for specialized functions that reflect the adaptive needs of each separate clade. Phagocytes in higher animals are neither totipotent, nor gatherers of nutrition, but they have retained the specialized function that allows them to recognize and engulf pathogens, or vestiges of apoptotic and nonself tissue. In general, the concept of a multifunctional cell type that has then compartmentalized a subset of its functions to form more specialized cells is a common theme seen in metazoan evolution [reviewed in Millar and Ratcliffe (1989)].

Like sponges, cnidarians are also diploblastic, with a largely acellular layer of mesoglea in between the ectoderm and the endoderm. Many species within this phylum do not have blood cells since diffusion of water and nutrients is fairly unrestricted in the mesoglea, often aided by symbiotic interactions with algae (for example, in corals). However, in a cnidarian such as the hydra, phagocytic blood cells populate and move through the mesoglea distributing nutrition (Cooper 1976). Recent studies provide evidence of Toll/NFΚB signaling in sea anemones, which raises the possibility that innate immunity preceded the traditional cnidarian–bilaterian split and might have evolved at about the same time as the most ancient blood cells (Brennan *et al.* 2017).

The first signs of additional differentiated blood cell types are seen with the evolution of the “pseudocoelom” in flatworms and nematodes, but the most rapid diversification and evolution of the blood tissue is observed with the advent of the true coelom in triploblastic animals that have evolved a well-defined mesodermal germ layer. Annelids have a closed loop circulatory system. Erythrocytes or “red blood cells” that carry oxygen to other body parts first appeared in marine (polychaete) annelids (Cooper 1976). Additionally, annelid blood contains cells that have been referred to as leukocytes, which are functionally akin to granulocytes, lymphocytes, and monocytes, as components of an immune system that can distinguish self from nonself [reviewed in Vetvicka and Šíma (2009)]. It is hypothesized that ancestors of annelids and other bilaterians might also have been coelomic, and that primitive blood cells arose from its linings; but in the absence of fossil data, it cannot be ruled out that the common ancestor had a “solid” mesenchyme within which the blood cells first arose. In either case, the bilaterians all built upon a basic ancestral framework, and generated diversity through the gain and loss of cell types depending on their respective adaptive strategies. This basic framework of the hematopoietic/vascular/innate immune system laid down in segmented worms is identifiable in molluscs [reviewed in Pila *et al.* (2016)], arthropods, echinoderms (starfish and sea urchins), and tunicates (sea squirts and ciona).

The phagocytic cell type first evident in sponges is considered homologous to macrophages. Additionally, the protostome invertebrates possess several blood cell types that are analogous to those seen in vertebrates. For example, crystal cells and lamellocytes in *Drosophila* have functions in common with platelets and giant cells in humans, but it is unlikely that these fly and mammalian cells arose through a monophyletic path. Finally, the lymphoid system is generally believed to have made its first appearance in bony fishes (osteichthyes), but scattered elements of this lineage are seen in the invertebrate deuterostomes (echinoderms, hemichordates, and chordates). There is good evidence that the vertebrate hematopoietic system, from fishes on to the mammals, follows a monophyletic evolutionary scheme that arose from the deuterostome invertebrates (echinoderms and tunicates) (Cooper 1976). For the purposes of this review on *Drosophila* hematopoiesis, based on currently available information, it is safe to assume that arthropod blood cells are restricted in their similarities to the myeloid, but not any of the lymphoid, cell types in mammals.

There is a close relationship between the development of the hematopoietic and vascular systems. With the appearance of the third body layer (the mesoderm) in triploblastic animals, a body cavity (the coelom) formed separating the endodermal and ectodermal layers. In most triploblasts, the blood–vascular system develops and functions in addition to the coelomic system. With the advent of vasculature, the circulating blood cells could reach longer distances of body length carrying nutrients and gases. Hartenstein and coworkers have reviewed the blood–vascular system from a phylogenetic point of view (Hartenstein 2006; Hartenstein and Mandal 2006; Grigorian and Hartenstein 2013). They point to the similarities of the endothelial layer lining the vertebrate vascular system with the mesothelium that lines (sometimes discontinuously) the coelom. Depending on the organism, the endothelial and/or mesothelial layers either directly give rise to blood precursors or to groups of cells that form structures such as the lymph gland in *Drosophila*, which constitutes a hematopoietic organ. In mammals, blood cells bud out of the endothelial lining of the dorsal aorta and ultimately migrate to individual sites of hematopoiesis, such as the fetal liver and the adult bone marrow [reviewed in Dzierzak and Speck (2008)]. In both mammals and *Drosophila*, the first set of hematopoietic and vascular cells share a common precursor, the so-called hemangioblast [reviewed in: Medvinsky and Dzierzak (1996), Ema and Rossant (2003), and Mandal *et al.* (2004)]. The larval hematopoietic organ (historically named the lymph gland) and the “dorsal vessel” (consisting of a pumping heart and aorta) both arise from this hemogenic mesodermal layer. It is not an uncommon mistake to refer to these tissues as “lymphoid organs,” which they are not. These organs serve very different purposes from the vertebrate lymph nodes/glands. Hematopoietic organs similar to the lymph gland have been seen in many arthropods ranging from insects to crustaceans, as well as in some molluscs [reviewed in Pila *et al.* (2016)]. However, other members of these phyla have dispersed sites of hematopoiesis along the mesothelium reminiscent of sessile sites of *Drosophila* larval, pupal, and adult subcuticular epidermal pockets. When comparing the mammalian and *Drosophila* blood–vascular systems, the conservation of molecular pathways is more impressive than the similarities between the tissue types. This suggests that molecular circuits that specify cell fate evolved early and these cassettes of genes work together as a unit, combined as necessary, to generate tissues sharing common functions.

A phylogenetic description of arthropod cell types is presented in several well-written reviews (Jones 1970; Ratcliffe and Rowley 1981; Pelc 1986; Lavine and Strand 2002; Ribeiro and Brehelin 2006). The principal classes of blood cell types are categorized as prohemocytes, plasmatocytes, granulocytes, oenocytoids, and spherule cells (or adipohemocytes). Prohemocytes (or hemocyte progenitors and preprogenitors) give rise to all differentiated blood cells. Plasmatocytes are phagocytic, arose in primitive metazoans, and are very likely directly related by homology to macrophages. They are also capable of further specialization through additional differentiation or fusion to form giant flattened cells. In *Drosophila*, differentiation of plasmatocytes can lead to cells that are named lamellocytes, while in mammals, giant cells arise through fusion of macrophages [reviewed in McNally and Anderson (2011)]. Granulocytes, missing in *Drosophila* but present in other Diptera (Kaaya and Ratcliffe 1982), contain acidic granules, but otherwise serve a similar phagocytic function as plasmatocytes. The same is true of adipohemocytes, also missing in *Drosophila*, which are likely to be another terminally differentiated form of plasmatocytes. Oenocytoids generally store prophenoloxidase, important for melanization, clotting, and the immune response. In *Drosophila*, the oenocytoids contain crystalline inclusions of prophenoloxidase and are called crystal cells. They perform many functions, served by analogous nonphagocytic cell types of the mammalian innate defense system that are unlikely to be related by direct homology. Yet, it is interesting to note that the transcription factor Lozenge, critical for specification of crystal cell fate, is an ortholog of Runx1, the earliest determinant of all mammalian blood cells [Lebestky *et al.* 2000; reviewed in Dzierzak and Speck (2008)].

Adult mammalian hematopoietic stem cells (HSCs) have been associated with several well-defined and established attributes. These include the ability to self-renew, to differentiate into all blood lineages, to be niche-dependent, and to repopulate the entire repertoire of blood cells for the long-term. In *Drosophila*, a majority of these criteria are met for the male and female germline stem cells and the stem cells of the intestine, all of which require self-renewal throughout the life of the animal. Similarly, neuroblasts have been referred to as neural stem cells due to their close association with and retention by a niche, their self-renewal and asymmetric cell division capabilities, and their label retention properties [reviewed in Morrison and Spradling (2008)]. During homeostasis, the prohemocytes of *Drosophila* are maintained by signals from a niche, they rapidly proliferate before decreasing their rates of division, and they subsequently differentiate to give rise to all blood cell types. A small number of these progenitors are inherited by adult flies (Ghosh *et al.* 2015), but their role in populating the adult repertoire needs to be further studied. It has not yet been established whether the progenitors divide asymmetrically to generate a copy of themselves and a differentiating daughter cell, which is often a hallmark of stem cells. The short life span of *Drosophila* also makes it uncertain if *de novo* adult hematopoiesis has a life-long replacement function, similar to that seen for germ and intestinal cells that are replaced on a daily basis. In this context, it is once again valuable to look at nonmodel systems through the lens of evolution. In long-lived crustaceans such as lobsters, blood progenitors continue to be maintained throughout life, generating all necessary blood cell types, and could reasonably be called stem cells. The archaeocytes in sponges are the ultimate, true stem cells, as they give rise to every cell type of the animal even when they are isolated from the adult. Yet, these cells are also phagocytic and likely direct phylogenetic ancestors to the mammalian macrophage lineage. It seems reasonable that terms such as stem cells should be defined in the developmental context of the stage, tissue, and species, rather than by a rigid set of criteria set in one single system. In the *Drosophila* lymph gland, cells close to the niche or the dorsal vessel may have the most permissive developmental potential (Minakhina and Steward 2010; Dey *et al.* 2016). However, to designate any group of hematopoietic cells in *Drosophila* as stem cells, they should fulfill at least one or more criteria, such as clear evidence of self-renewal or asymmetric cell division. Until then, it seems most appropriate to call these cells preprohemocytes or preprogenitors, which is how we refer to them in this article.

## Drosophila Blood Cell Types

Three morphologically distinct types of mature hemocytes have been identified in *Drosophila*: plasmatocytes that have phagocytic and antimicrobial functions; crystal cells that facilitate wound healing, innate immunity, and the hypoxic response; and lamellocytes, which are specialized cells that primarily respond to wasp parasitization [Rizki and Rizki 1979; reviewed in Evans *et al.* (2003) and Letourneau *et al.* (2016)]. Plasmatocytes comprise ∼90–95% of hemocytes and crystal cells account for ∼2–5% during embryogenesis, in larvae, and in adults (Tepass *et al.* 1994; Lebestky *et al.* 2000; Ghosh *et al.* 2015; Leitão and Sucena 2015). Lamellocytes are rarely seen in healthy larvae, but are induced upon deposition of eggs by parasitic wasps (Rizki and Rizki 1992). The three cell types were first distinguished based on their ultrastructure and cytochemistry, and later refined through studies of biological function, as well as expression patterns of cell surface antigens, enhancer traps, and transcription factors that specify hemocyte fate (Shrestha and Gateff 1982; Rodriguez *et al.* 1996; Braun *et al.* 1997; Lo *et al.* 2002; Tirouvanziam *et al.* 2004; Kurucz *et al.* 2007b).

Many of the genes used as markers are also functionally significant for the individual blood cell types. Similar to mammalian macrophages, plasmatocytes eliminate both apoptotic cells and invading particles (Franc *et al.* 1996). During embryogenesis, plasmatocytes primarily function to endocytose apoptotic cells and secrete extracellular matrix (ECM) proteins as they aid in tissue remodeling (Olofsson and Page 2005; Bunt *et al.* 2010). Plasmatocytes express the free radical scavenging enzyme Peroxidasin as well as several cell-surface molecules involved in phagocytosis, such as the receptors Nimrod C1 (P1 antigen) and Eater (Nelson *et al.* 1994; Kocks *et al.* 2005; Kurucz *et al.* 2007a,b; Bretscher *et al.* 2015). Crystal cells contain crystalline inclusions of the prophenoloxidase (ProPO) enzymes, which mediate melanization, and these cells primarily function to initiate the melanization cascade during injury and the innate immune response (Binggeli *et al.* 2014; Dudzic *et al.* 2015). Melanization leads to a darkening and hardening of damaged tissue that assists in wound healing, while the free radicals produced during the melanization process neutralize pathogens. Lamellocytes are large, flat cells that encapsulate wasp eggs that are injected by the parasite’s ovipositor through the *Drosophila* larval cuticle (Rizki and Rizki 1992). βPS-integrin (encoded by *myospheroid*; *mys*) is highly expressed in lamellocytes, and while not required for differentiation, it is required for the encapsulation response by lamellocytes (Irving *et al.* 2005).

### Plasmatocytes

#### Summary:

Over the years, ultrastructural analyses have revealed intriguing differences between morphologically distinct subsets of plasmatocytes [Shrestha and Gateff 1982; Rizki and Rizki 1984; Lanot *et al.* 2001; Grigorian *et al.* 2011b; reviewed in Grigorian and Hartenstein (2013) and Grigorian *et al.* (2013)]. These likely represent different stages of plasmatocyte maturation as it has proven more difficult to distinguish between them using molecular markers. Such markers include Hemolectin (Hml), Peroxidasin (Pxn), NimC1 (P1 antigen), Croquemort (Crq), Collagen, and Eater (Tepass *et al.* 1994; Franc *et al.* 1996; Asha *et al.* 2003; Sinenko and Mathey-Prevot 2004; Jung *et al.* 2005; Olofsson and Page 2005; Kurucz *et al.* 2007a,b; Sorrentino *et al.* 2007; Stofanko *et al.* 2008; Tokusumi *et al.* 2009a; Makhijani *et al.* 2011; Evans *et al.* 2014). While each of these is a reasonably representative identifier of the plasmatocyte fate, none is 100% effective in marking every plasmatocyte and, at least in some instances, this might represent differences in the differentiation/maturation process. This variability usually does not pose a serious problem for most analyses, although future studies combining ultrastructure with marker analysis may help elucidate the basis for this variability in marker expression patterns. An antibody against the P1 antigen is widely used to identify mature plasmatocytes in larvae and adult flies, but does not recognize embryonic plasmatocytes (Kurucz *et al.* 2007a). On a practical note, it is worth keeping in mind that a large number of commonly used *Drosophila* stocks and chromosomes are “P1-negative” (Honti *et al.* 2013). This variation is inherited as a recessive trait, and can therefore confound clonal and other forms of immunohistochemical analyses that utilize staining of the heterozygous tissue as a control. Coupled with the fact that the total blood cell number in an animal can show a considerable degree of variation, one cannot overstate the importance of proper statistical analysis in studies of blood phenotypes.

In addition to phagocytosis of apoptotic cells, embryonic plasmatocytes function in the secretion of ECM proteins. During the second-half of embryogenesis, all cell surfaces that are in contact with the hemolymph become covered with basement membranes due to the widespread secretion of ECM molecules from the blood cells and the fat body [reviewed in Fessler and Fessler (1989), Tepass *et al.* (1994), and Martinek *et al.* (2008)]. An exception is the cell surface of the circulating hemocytes themselves that constitute a major source of these ECM molecules, including Papilin, Laminin, Collagen IV, Glutactin, and Tiggrin (Tig) [reviewed in Fessler and Fessler (1989), Kusche-Gullberg *et al.* (1992), and Fogerty *et al.* (1994)]. Regulated macrophage migration is essential for uniform delivery of matrix proteins such as Collagen IV, Perlecan, and Laminin A (Matsubayashi *et al.* 2017; Sánchez-Sánchez *et al.* 2017). Deposition of these basement membrane components by hemocytes is crucial for embryonic renal tubule morphogenesis (Bunt *et al.* 2010). These key functions may explain why embryos depleted of plasmatocytes have very low viability (Defaye *et al.* 2009; Shia *et al.* 2009). Basement membrane deposition by plasmatocytes is also important for later stages of development. For example, plasmatocytes are known to associate with the adult ovarian stem cell niche where they deposit ECM components to form the basement membrane (Van De Bor *et al.* 2015). Niche signaling, morphology, and stem cell number are all adversely affected if ECM components are knocked down in hemocytes, or if the plasmatocytes are depleted (Van De Bor *et al.* 2015). Thus, although larvae depleted of a majority of plasmatocytes can survive to adulthood, these animals might have defects in organogenesis.

#### Specifics:

A detailed mechanistic description of how embryonic plasmatocytes detect, engulf, and degrade apoptotic corpses is beyond the scope of this article, but this is an intensely studied field, recently reviewed by experts (Ulvila *et al.* 2011; Wood and Martin 2017). In brief, plasmatocytes express several proteins in combination that identify entities on the surface of cells they engulf, including lipids such as phosphatidylserine (Tung *et al.* 2013). The cell-surface proteins involved in this recognition process include, but are not limited to, Crq, an ortholog of the vertebrate CD36 scavenger receptor, the CED-1 homolog Draper, isoforms of the immunoglobulin-superfamily receptor Dscam (Down syndrome cell adhesion molecule), and the α-PS3 (encoded by *scab*)/Integrin βν heterodimer (Franc *et al.* 1996; Manaka *et al.* 2004; Watson *et al.* 2005; Nonaka *et al.* 2013). Six-microns-under (encoded by *NimC4*), a CED-1/Draper family phagocytosis receptor, is expressed in both phagocytic glial cells and plasmatocytes (Kurant *et al.* 2008). A prominent signaling cascade downstream of apoptotic engagement involves Pallbearer, an F-box E3 ubiquitin ligase that is a subunit of a Skp/Cullin/F-box complex, which promotes proteasomal degradation (Xiao *et al.* 2015). Interaction of an apoptotic cell with plasmatocytes bearing receptors such as Draper induces the release of intracellular calcium and this mechanism is essential for efficient phagocytosis of the apoptotic cell (Cuttell *et al.* 2008). This calcium flash also causes a further increase in the expression of Draper, which primes the macrophages during development for further engulfment activity during injury and infection (Weavers *et al.* 2016a). When phagocytosis is blocked either by depleting plasmatocytes or eliminating Crq, the embryonic ventral nerve cord fails to condense, illustrating the importance of phagocytosis of apoptotic cells for proper embryogenesis (Sears *et al.* 2003; Defaye *et al.* 2009; Guillou *et al.* 2016). The most prominent developmental function in the absence of any infection is evident during pupal development when plasmatocytes remove large amounts of cellular debris that result from the remodeling activities associated with metamorphosis (Lanot *et al.* 2001; Regan *et al.* 2013).

The transmembrane receptor Eater binds to bacterial surfaces, and is expressed in both mature and immature plasmatocytes (Kocks *et al.* 2005; Kroeger *et al.* 2012). This protein assists in efficient phagocytosis of both *Eschericia coli* and *Staphylococcuc aureus* bacteria *in vitro* and *in vivo*. *eater* null flies are more susceptible than wild-type to infection by bacterial pathogens (Kocks *et al.* 2005; Charroux and Royet 2009; Defaye *et al.* 2009). Mature plasmatocytes produce antimicrobial peptides (AMPs) in response to bacterial challenge of larvae (Irving *et al.* 2005; Kurucz *et al.* 2007b). However, the major roles of hemocytes in the host defense system are the phagocytosis of microorganisms, surveillance of damaged tissue, and the first stages of encapsulation of large parasitic invaders (Russo *et al.* 1996; Babcock *et al.* 2008; Pastor-Pareja *et al.* 2008; Charroux and Royet 2009; Defaye *et al.* 2009; Kelsey *et al.* 2012; Anderl *et al.* 2016).

The *Drosophila* GATA protein Serpent (Srp) is a master regulator of early hemocyte specification. Srp directly controls *eater* expression at the transcriptional level. The *eater* minimal enhancer transgene often serves as a marker for mature plasmatocytes; however, the endogenous expression of the gene seems less restricted. Widespread distribution of *eater* mRNA is seen throughout the primary and secondary lobes of the lymph gland, consistent with their ubiquitous Srp expression (Kocks *et al.* 2005; Tokusumi *et al.* 2009a; Kroeger *et al.* 2012). A different GATA transcription factor, Pannier (Pnr), along with JAK/STAT signaling plays a role in plasmatocyte development in the lymph gland (Minakhina *et al.* 2011). The gene pair *glide/glial cells missing (gcm)* and *glial cells missing 2 (gcm2)*, a critical determinant of the embryonic plasmatocyte fate, is also controlled by Srp (Bernardoni *et al.* 1997). A genome-wide screen determined that transcriptional targets of Gcm include components of the Notch, Hedgehog (Hh), Wingless (Wg)/Wnt, Fibroblast Growth Factor (FGF) Receptor (FGFR), and JAK/STAT signaling pathways, all known to be important for hemocyte differentiation (Cattenoz *et al.* 2016). Gcm and Gcm2 play a role in the terminal differentiation of functional plasmatocytes, since hemocytes in double-mutant animals express the early marker Pxn but fail to express the late marker Crq. Single mutants of *gcm* or *gcm2* reduce the number of hemocytes that express Crq, while a large deficiency removing both genes eliminates all Crq^+^ cells (Alfonso and Jones 2002). Similarly, *gcm*/*gcm2* double mutants lack expression of the phagocytosis receptor Draper, another indication that the hemocytes are not terminally differentiated (Freeman *et al.* 2003). *gcm* is expressed prior to *Pxn* in embryonic hemocytes and overexpression of *gcm* results in an increase in Pxn^+^ cells (Bernardoni *et al.* 1997). During stages 12–13 of wild-type embryogenesis, functional macrophages begin to terminally differentiate and spread throughout the embryo, and at this stage hemocytes cease expression of *gcm*/*gcm2*. However, plasmatocytes fail to migrate in a *gcm/ gcm2* double-mutant background, perhaps reflecting a secondary effect due to improper maturation of the plasmatocytes (Alfonso and Jones 2002). While Gcm is critical for embryonic hematopoiesis, it remains unclear whether it plays a role in the lymph gland due to the fact that *gcm-GAL4*, one of the few reagents available to study Gcm function, is not expressed in the lymph gland or circulating cells in larvae (Avet-Rochex *et al.* 2010).

### Crystal cells

#### Summary:

Crystal cells can be morphologically distinguished from plasmatocytes due to their lack of cytoplasmic processes and by their less electron dense cytoplasm due to fewer ribosomes (Shrestha and Gateff 1982). A quick and easy, though coarse, way to visualize larval crystal cells through the cuticle is by heating/boiling the larvae, which allows activation of the melanization cascade within the crystal cells (Rizki 1957; Lanot *et al.* 2001). Crystal cells can be more reliably identified in the *Black cells* (Babcock *et al.* 2008) mutant (a dominant mutation in *PPO1*), as well as by using reporter constructs and antibodies against early markers such as Lozenge (Lz) or Pebbled/Hindsight (Hnt), and the late marker ProPO (Lebestky *et al.* 2000; Jung *et al.* 2005; Gajewski *et al.* 2007; Tokusumi *et al.* 2009a; Benmimoun *et al.* 2012; Terriente-Felix *et al.* 2013; Evans *et al.* 2014). ProPO is inactive within crystal cells but, upon release, is activated by a proteolytic cascade that involves Hayan and Sp7 (also known as MP2 or PAE1) (Tang *et al.* 2006; Nam *et al.* 2012). Serine protease inhibitors (Serpins) prevent aberrant activation of this cascade. When released from crystal cells, activated phenoloxidase (PO) converts tyrosine-derived phenols to quinones, which in turn polymerize to form melanin [reviewed in Cerenius *et al.* (2008)]. Three separate genes—*PPO1*, *PPO2*, and *PPO3*—encode ProPO enzymes in *Drosophila* (Binggeli *et al.* 2014; Dudzic *et al.* 2015). PPO3 is expressed in lamellocytes while crystal cells express both PPO1 and PPO2, which are the primary enzymes involved in melanization after injury (Irving *et al.* 2005; Binggeli *et al.* 2014; Dudzic *et al.* 2015). Although both PPO1 and PPO2 are expressed in crystal cells, only PPO2 has been shown to colocalize with the crystalline inclusions, suggesting that PPO1 might be secreted into the hemolymph (Binggeli *et al.* 2014).

Crystal cells are first seen in the embryo, but their function at this stage is unclear. However, during injury or infection in the larval stages, crystal cells help in wound healing and the immune response to pathogens through their PO activity. Larvae deficient for PPO1 and PPO2 are rendered more susceptible to microbial infection (Binggeli *et al.* 2014; Dudzic *et al.* 2015), possibly due to an absence of cytotoxic reactive oxygen species (ROS) generated as by-products of the melanization cascade [reviewed in Nappi *et al.* (1995)]. ROS also play a role in signaling during wound healing (Nam *et al.* 2012). Larvae and adult flies deficient in melanization (*e.g.*, in *Bc* or *lz* mutants) exhibit less-efficient wound healing and a high mortality rate after wounding (Rizki *et al.* 1980; Rämet *et al.* 2002; Galko and Krasnow 2004; Neyen *et al.* 2015).

The Runx domain transcription factor Lz, the closest invertebrate ortholog of Runx1 (also called acute myeloid leukemia-1 or AML1 in humans), is the critical transcription factor necessary for crystal cell specification, as well as differentiation during both embryonic and larval hematopoiesis (Lebestky *et al.* 2000). Both the transcript and the protein are detected by stage 10 of embryogenesis, and mature crystal cells become evident shortly thereafter in stage 11/12 (Fossett *et al.* 2003; Bataille *et al.* 2005). Temperature shift experiments involving *lz^ts1^;Bc* embryos demonstrate that Lz function is continuously required during stages 10–14 for crystal cell development. Lz^+^ crystal cell precursors arise from a subset of Serpent-expressing cells, both in the embryonic head mesoderm and in the larval lymph gland, where cells expressing *lz* are first seen during the second instar, and continuously increase in number through the third instar. These Lz^+^ crystal cell precursors are scattered among the differentiating cells of the lymph gland (Lebestky *et al.* 2000). Crystal cells are completely lost in both embryos and larvae when Lz activity or expression is blocked, while plasmatocyte development remains unaffected. Wg signaling also seems to play a role in crystal cell formation since expression of a dominant negative form of the Wg receptor Frizzled-2 (Fz2) [but not Frizzled (Fz)] in Lz^+^ crystal cells reduces their number. Wg is expressed in some but not all Lz^+^ cells, and its overexpression causes an increased number of crystal cells (Sinenko *et al.* 2009).

Throughout the early *Drosophila* life cycle, crystal cells comprise only 2–5% of total hemocyte numbers under normal conditions (Lanot *et al.* 2001; Kurucz *et al.* 2007b). A complex regulatory circuit involving Srp, Lz, and the Friend of GATA homolog U-shaped (Ush) functions to both specify crystal cells, and also limit their number. Srp and Lz physically interact, and synergize to activate an autoregulatory loop that controls *lz* transcription and specifies crystal cell fate. Thus, forced expression of Lz can only induce crystal cells in Srp^+^ cells and loss of Srp reduces Lz expression. Following the initial specification of the crystal cell lineage, Srp and Lz function together to upregulate Ush expression, which in turn functions with Srp to limit Lz expression, and thus control the number of crystal cells. Srp and Lz together play a later role in the control of ProPO expression in mature crystal cells (Fossett *et al.* 2001, 2003; Waltzer *et al.* 2002, 2003; Muratoglu *et al.* 2006; Ferjoux *et al.* 2007; Gajewski *et al.* 2007).

The major signaling pathway required for crystal cell differentiation is Notch. Crystal cells are reduced in the head mesoderm and lymph glands in *Notch* and *Suppressor of Hairless* [*Su(H)*] mutants (Duvic *et al.* 2002; Lebestky *et al.* 2003; Bataille *et al.* 2005). Lz is a direct transcriptional target of this pathway and its expression is decreased in these mutants. Notch transcriptional activity remains unchanged in an *lz* mutant background showing that Lz functions downstream of Notch (Lebestky *et al.* 2003). Notch and Lz function together to activate target genes *klumpfuss* and *pebbled/hindsight*, which promote crystal cell differentiation, and prevent them from assuming alternate fates (Terriente-Felix *et al.* 2013). In addition to specifying crystal cell fate, Notch signaling also plays an earlier role in cell proliferation as lymph gland clones mutant for Notch or Su(H) are smaller than their wild-type counterparts (Lebestky *et al.* 2003; Dey *et al.* 2016).

#### Specifics:

Serrate (Ser), rather than Delta is the Notch ligand involved in promoting Lz expression in the lymph gland (Duvic *et al.* 2002; Lebestky *et al.* 2003). Ser protein is expressed in scattered cells found in close proximity to crystal cells throughout the lymph gland, which arise from Dome^+^ progenitors but do not contribute to the crystal cell lineage (Lebestky *et al.* 2003; Crozatier *et al.* 2004; Ferguson and Martinez-Agosto 2014). Instead, the Ser^+^ cells also express Yorkie (Yki) and Scalloped (Sd), and the Yki pathway is essential for proper Ser expression and crystal cell formation (Milton *et al.* 2014). Depletion of Yki or Sd in Ser^+^ cells results in a significant decrease in ProPO^+^ cells (Ferguson and Martinez-Agosto 2014).

Following the initial specification and differentiation of a Lz^+^ cell by the canonical Ser/Notch signal, the maturation and maintenance of the crystal cell fate requires a second, noncanonical function of Notch. Lz induces nitric oxide (NO) synthase, which uses arginine as a substrate to produce NO in the developing crystal cell. In a manner similar to ROS, NO stabilizes Sima, the *Drosophila* ortholog of the mammalian hypoxia-inducible factor-α (Hif-α), protein by inactivating its binding partner involved in Sima degradation. The stabilized Sima protein is able to form a complex with the intracellular domain of Notch, and together this complex binds Su(H) and activates a unique set of genes that is not involved in the hypoxia response. Nevertheless, upon exposure to hypoxic conditions, additional Sima protein is stabilized, giving rise to more robust crystal cell formation and maintenance. Loss of Sima has no effect on the initial specification of the crystal cell, but such loss leads to a bursting phenotype due to the crystal cell’s inability to maintain its integrity. Overexpression of Sima in either Lz^+^ or Hml^+^ cells causes a dramatic expansion of crystal cells. Removal of Ser early (50–60 hr after egg lay) decreases crystal cell numbers, but late removal (60–76 hr after egg lay) does not affect this population. Additional experiments manipulating downstream effectors of Ser/Notch interaction support the idea that Sima functions in a ligand-independent Notch signaling pathway to stabilize full-length Notch and maintain crystal cell numbers (Mukherjee *et al.* 2011).

### Lamellocytes

#### Summary:

While not typically seen in healthy animals, lamellocytes are induced during larval stages under stress conditions such as wasp parasitization, injury, or in the presence of abnormal/damaged tissues. These cells are large, flat, disc-shaped, and show irregular margins with cytoplasmic processes and a relatively small nucleus (Shrestha and Gateff 1982). Lamellocytes typically contain more lysosomes and phagocytic vacuoles than plasmatocytes although they do not exhibit phagocytic ability (Lanot *et al.* 2001; Kurucz *et al.* 2007b). Lamellocytes are detected in white prepupae, but not in adult animals (Rizki 1957; Shrestha and Gateff 1982; Lemaitre *et al.* 1995). Good markers for lamellocytes include Atilla, β-PS integrin (encoded by *myospheroid*, *mys*), α-PS4 integrin, Misshapen (Msn), Puckered, PPO3, and L6 or L2 antigens [Irving *et al.* 2005; Kurucz *et al.* 2007b; Nam *et al.* 2008; Honti *et al.* 2009; Tokusumi *et al.* 2009b; reviewed in Evans *et al.* (2014), Honti *et al.* (2014), Dudzic *et al.* (2015), and Anderl *et al.* (2016)]. Mys is also expressed in hemocyte progenitors and in plasmatocytes, and while Mys is not required for lamellocyte differentiation, the encoded integrin is essential for the encapsulation of parasitoid wasp eggs and the formation of melanotic tumors (Irving *et al.* 2005; Stofanko *et al.* 2008). The Rho-GTPase Rac1 allows proper localization of Mys to the cellular periphery of lamellocytes and participates in the activation of Focal adhesion kinase in lamellocytes during the encapsulation process (Williams *et al.* 2006; Xavier and Williams 2011). The L1-type cell adhesion protein Neuroglian is also localized to the cell periphery and is required for proper encapsulation of wasp eggs (Williams 2009). As detailed below, the Notch, JAK/STAT, JNK, Toll, EGFR, and ecdysone pathways all contribute to the production of this single cell type, the lamellocyte (Sorrentino *et al.* 2002, 2004; Kurucz *et al.* 2003; Zettervall *et al.* 2004; Williams *et al.* 2006; Small *et al.* 2014; Louradour *et al.* 2017).

#### Specifics:

Larval circulating plasmatocytes are activated upon wasp parasitization. These plasmatocytes adhere to the wasp egg and are reported to transdifferentiate into “type II” lamellocytes. This is indicated by their expression of Msn. However, these cells retain Eater and P1 expression, normally seen only in plasmatocytes. In contrast, circulating “type I” lamellocytes do not show significant levels of Eater expression and arise from “lamelloblast” or “prelamellocyte” precursor populations. Unlike mature lamellocytes, the progenitor populations incorporate EdU and proliferate. The previously identified L-antigens show different temporal patterns in the different hemocyte populations after parasitization and can be used in some cases to identify the subpopulations of cells (Anderl *et al.* 2016).

The JNK (Basket, Bsk) pathway plays a pivotal role in specifying lamellocyte fate. Components of this pathway—Bsk, Msn, Puckered (Puc), Hemipterous (Hep), Kayak (Kay; Fos), and FOXO—are all involved in this process (Braun *et al.* 1997; Zettervall *et al.* 2004; Williams *et al.* 2006; Stofanko *et al.* 2008; Tokusumi *et al.* 2009b, 2017). Overexpression of Kay, Rac1, FOXO, or a constitutively active Hep in hemocytes causes lamellocytes to differentiate in the absence of wasp parasitization (Zettervall *et al.* 2004; Williams *et al.* 2006; Stofanko *et al.* 2008; Tokusumi *et al.* 2017). Kay is required for lamellocyte-specific *msn* enhancer activity. Loss of either Kay or FOXO impairs lamellocyte production in parasitized larvae (Tokusumi *et al.* 2009b, 2017).

Toll signaling also plays a role in lamellocyte formation as constitutive activation of Toll (using *Toll^10B^* or *Toll^9Q^* mutations), overexpression of Dorsal, or loss of Cactus causes increased numbers of circulating lamellocytes by a process that involves both the fat body and blood cells (Lemaitre *et al.* 1995; Qiu *et al.* 1998; Schmid *et al.* 2014). Overexpression of *Toll^10B^* or loss of Jumeau (Jumu), a member of the forkhead transcription factor family, throughout the lymph gland, induces lamellocyte differentiation as well as nuclear translocation of Dif (Dorsal-related immunity factor), indicating a cell autonomous role of Toll activation in lamellocyte differentiation in the lymph gland (Hao and Jin 2017). Loss of *ird1* (*immune response-deficient 1*) enhances lamellocyte formation in *Toll^10B^* mutants and in fact induces lamellocyte formation on its own (Schmid *et al.* 2016). Toll also plays a role in the cellular response to wasp parasitization (Louradour *et al.* 2017).

The GATA protein Srp and the Friend of GATA homolog Ush are also involved in lamellocyte differentiation. Loss of even a single copy of *ush* results in a significant increase in the number of lamellocytes in circulation, a phenotype suppressed by a concurrent single-copy loss of *srp* (Gajewski *et al.* 2007). Overactivation of the JAK/STAT signal in hemocytes increases lamellocyte differentiation in the absence of wasp infestation (Harrison *et al.* 1995; Luo *et al.* 1995, 1997; Zettervall *et al.* 2004). This induction of supernumerary lamellocytes is in part due to the positive regulation of Ush by JAK/STAT signaling (Gao *et al.* 2009). Overexpression of Ush and loss of the *Drosophila* JAK (encoded by *hopscotch*, *hop*) both reduce circulating lamellocyte numbers in response to wasp parasitization (Sorrentino *et al.* 2004, 2007). However, the role of JAK/STAT signaling in lamellocyte differentiation is more complex and might involve nonautonomous and systemic signaling. Single-cell clones that overexpress *hop* cause both autonomous and nonautonomous induction of lamellocytes within the lymph gland (Minakhina *et al.* 2011). As an example of systemic effects, knockdown of JAK/STAT signaling in body wall muscle cells impedes postparasitization differentiation of lamellocytes. One explanation for these results is that JAK/STAT activation in hemocytes causes them to secrete Upd2/Upd3, which in turn activates JAK/STAT signaling in body wall muscles (Yang *et al.* 2015). In contrast, JAK/STAT signaling is switched off in lymph gland progenitors following wasp parasitization to allow lamellocyte differentiation. Unlike JAK/STAT, the role of Notch signaling in lamellocyte differentiation is less well studied, although it is reported that lamellocyte formation in the lymph gland is inhibited by Notch (Small *et al.* 2014).

## Sites of Hematopoietic Development

**Figure 2:**
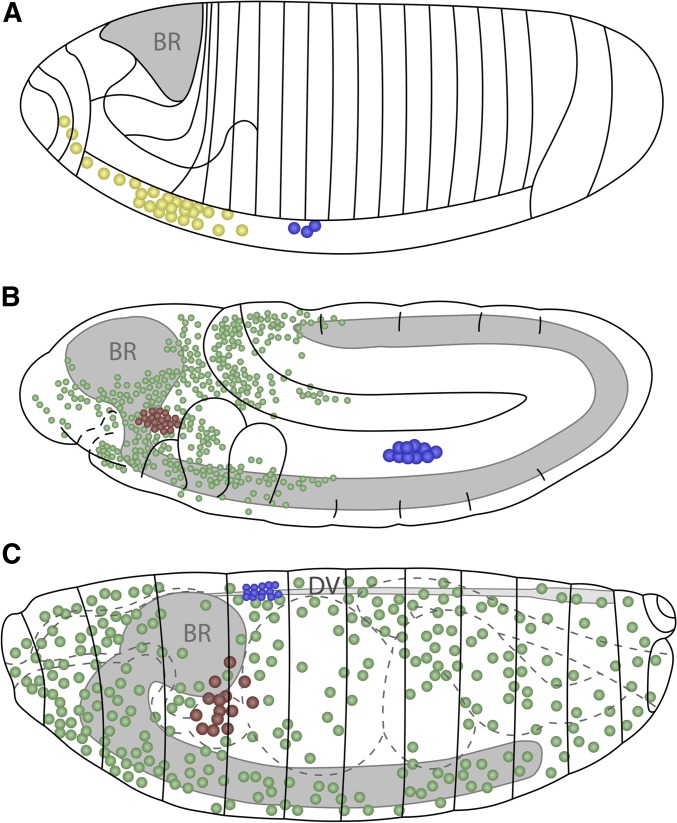
Embryonic hematopoiesis. (A) Stage 5 embryo. Precursors for embryonic hemocytes (yellow) are specified from the head mesoderm, while lymph gland precursors (blue) arise from the thoracic region of the dorsal mesoderm. BR, gray. (B) Stage 11 embryo. Embryonic prohemocytes migrate and differentiate into plasmatocytes (green) and crystal cells (red). The lymph gland anlage proliferate and are seen in the trunk region. (C) Stage 17 embryo. Plasmatocytes migrate throughout the embryo, while crystal cells accumulate near the proventriculus. During dorsal closure, the lymph gland precursors on either side of the embryo move dorsally and are positioned flanking the DV. Later, these cells will constitute the lymph gland with pairs of distinguishable primary and posterior lobes. Schematics in (A–C) adapted from Volker Hartenstein, see Lebestky *et al.* (2000). BR, brain; DV, dorsal vessel.

The process of hematopoiesis, defined as the segregation of blood cells from a broader group of mesodermal precursors, occurs in two waves in the *Drosophila* embryo (Figure 2). The first wave originates in the procephalic or head mesoderm, and gives rise to both circulating and sessile pools of hemocytes that populate all four life stages of this holometabolous insect (Tepass *et al.* 1994; Holz *et al.* 2003; Honti *et al.* 2010; Ghosh *et al.* 2015). The second wave begins in the dorsal mesoderm giving rise to the dorsal vessel and the lymph gland that are, respectively, the heart–aorta that controls the open circulation of hemolymph and the major hematopoietic organ during the larval stages (Rugendorff *et al.* 1994). Cells from the lymph gland, together with cells derived from the head mesoderm, eventually contribute to the adult hemocyte population (Holz *et al.* 2003; Ghosh *et al.* 2015).

While the exact total number of blood cells in *Drosophila* varies with stage and environmental effects, it is generally recognized that several hundred blood cells are made in the embryo (Tepass *et al.* 1994). This number decreases somewhat at hatching and then expands through the larval stages to > 5000 hemocytes during the pupal stage (Lanot *et al.* 2001; Makhijani *et al.* 2011). At this point, there is a high demand for blood cells to accommodate the extensive histolysis and tissue reengineering during metamorphosis (Lanot *et al.* 2001; Regan *et al.* 2013). The total number of hemocytes in adults likely ranges between 1000 and 2000 cells per animal (Lanot *et al.* 2001).

### Embryonic procephalic mesoderm

#### Summary:

During the first wave of hematopoiesis, hemocytes originate from the procephalic or head mesoderm of the embryo (Tepass *et al.* 1994; Holz *et al.* 2003) (Figure 2A). In contrast to other later-developing mesodermal cell types, transplantation studies show that embryonic hemocytes are already specified at the cellular blastoderm stage (stage 5) (Holz *et al.* 2003). The head mesoderm undergoes four divisions during embryonic stages 8–11. After the final division, the majority of head mesoderm cells (∼300 on either side of the embryo) are recognizable as hemocytes and this number remains constant throughout the rest of embryogenesis (Tepass *et al.* 1994). During stages 10–12, the head mesoderm also contains a cluster of 20–30 crystal cell precursors (Lebestky *et al.* 2000).

Several factors first identified as important regulators of mammalian hematopoiesis are also essential for embryonic hematopoiesis in *Drosophila*. In particular, the GATA, Friend of GATA (FOG), and RUNX protein families are conserved hematopoietic regulators that act combinatorially to regulate lineage commitment in *Drosophila* hematopoiesis. The GATA factor Serpent (Srp) is required for the development and differentiation of both plasmatocytes and crystal cells. This regulator controls key proteins such as Gcm, Lz, and Ush, which are necessary for hemocyte development [Bernardoni *et al.* 1997; Lebestky *et al.* 2000; Fossett *et al.* 2001; Alfonso and Jones 2002; Waltzer *et al.* 2002; reviewed in Evans *et al.* (2003)].

#### Specifics:

In many ways, the process of procephalic hematopoiesis in *Drosophila* is, at least superficially, similar to initial waves of mammalian hematopoiesis that give rise to early and intermediate progenitors. Analogous to primitive hematopoiesis, the first wave that occurs in mammals shares many common molecular strategies observed during this phase of *Drosophila* blood development [reviewed in Evans *et al.* (2003)]. Of the six known mammalian GATA factors, GATA-1, -2, and -3 are involved in hematopoiesis [reviewed in Orkin and Zon (2008)]. Mammalian GATA-1/2 are required early during the specification of blood progenitors undergoing primitive erythropoiesis (Fujiwara *et al.* 2004). Depending on the context, members of the FOG family of proteins enhance or inhibit GATA transcription factor activity [reviewed in Cantor and Orkin (2005)]. Another early marker for mammalian hematopoiesis is the RUNX family transcription factor RUNX1 (also known as AML1), which is essential for the very first steps of blood formation within the dorsal aorta and later in many other hematopoietic processes. Chromosomal translocations into this locus are responsible for a variety of human leukemias (Okuda *et al.* 1996; Yokomizo *et al.* 2001; Chen *et al.* 2009). Each of these transcription factor classes also plays critical roles in *Drosophila* hematopoiesis, including the GATA factors Serpent (Srp) and Pannier (Pnr), the FOG homolog Ush, and the RUNX domain protein Lozenge (Lz).

Srp is a critical regulator of hematopoiesis that is first expressed in the head mesoderm of stage-5 embryos (Abel *et al.* 1993; Sam *et al.* 1996; Brückner *et al.* 2004). Later-stage *srp* mutant embryos are devoid of all mature hemocytes (Rehorn *et al.* 1996). Srp expression in hemocyte precursors is controlled through the combinatorial activity of Snail, Buttonhead, Empty spiracles, and Even-skipped, which together confine Srp expression to the head mesoderm (Yin *et al.* 1997; Spahn *et al.* 2014). Indeed, ectopic expression of Srp in the trunk mesoderm, even in the absence of head mesoderm as in *bicoid* (*bcd*) mutant embryos, induces the formation of hemocytes and the fat body at the expense of other mesodermal tissues. Snail and Buttonhead, with a minor contribution from Empty spiracles, drive early Srp expression while Even-skipped, expressed posterior (P) to the Srp region, negatively regulates Srp expression limiting it to the head mesoderm (Spahn *et al.* 2014).

At the end of blastoderm stage 5, Srp^+^ hemocyte precursors begin to form distinct subsets from which the plasmatocyte and crystal cell populations are derived. The majority of Srp^+^ cells begin to express Gcm and Gcm2, which are both required for terminal plasmatocyte differentiation (Bernardoni *et al.* 1997; Alfonso and Jones 2002). A small subset of Srp^+^ cells in the embryonic head mesoderm do not express Gcm but express Lz, the key crystal cell determinant (Lebestky *et al.* 2000). It is essential for Gcm expression to be downregulated to form a mature crystal cell. A combined loss of both Gcm and Gcm2 leads to an increase in the Lz^+^ population. Gcm/Gcm2 are initially expressed in all blood precursors, but are then downregulated specifically in the most anterior (A) row of cells, which begin to express Lz. There are then two potential fates for Lz^+^ cells: in the continued absence of Gcm/Gcm2 in crystal cell progenitors near the proventriculus they become mature crystal cells, but when Gcm/Gcm2 is expressed in Lz^+^ cells distant from the proventriculus they become plasmatocytes (Bataille *et al.* 2005). Overexpression of Gcm with *lz-GAL4* represses crystal cell fate by inhibiting Lz expression, while Lz is unable to override the plasmatocyte fate even when ubiquitously expressed in the head mesoderm (Lebestky *et al.* 2000; Waltzer *et al.* 2003).

As early as stage 8 of embryogenesis, Srp also controls the expression of Ush, which together with Srp and Lz plays important roles in crystal cell production. The contribution of these proteins has been carefully refined to reveal that in Srp^+^Lz^+^ crystal cell precursors, Srp and Lz promote crystal cell lineage commitment. In Srp^+^Gcm^+^ plasmatocyte precursors, Srp functions with Ush to suppress crystal cell fate and induce plasmatocyte differentiation genes. A complex feedback circuit with Srp, Lz, and Ush functions to both specify crystal cells and limit their production (Fossett *et al.* 2001, 2003; Waltzer *et al.* 2002, 2003; Muratoglu *et al.* 2006).

Prior to embryonic stage 12, the morphology of the blood cells is similar to the ultrastructure of prohemocytes, which are typically small, round mesodermal cells (Tepass *et al.* 1994). A total of ∼700 hemocytes begin spreading throughout the embryo at the beginning of germ band retraction (early stage 12). These prohemocytes migrate and begin to differentiate, morphologically resembling plasmatocytes and become highly polarized with dynamic, large, actin-rich filopodia and lamellipodia, which continually extend and retract (Tepass *et al.* 1994; Wood *et al.* 2006). These cells now exhibit phagocytic activity as they engulf apoptotic cells within the developing tissues (Tepass *et al.* 1994) (Figure 2, B and C). Plasmatocytes continue to spread through stages 13–14, migrating medially from either end of the embryo along three specific developmentally regulated routes directed by the PDGF/VEGF receptor (Pvr) ligands Pvf2 and Pvf3 [Tepass *et al.* 1994; Heino *et al.* 2001; Alfonso and Jones 2002; Cho *et al.* 2002; Wood *et al.* 2006; Parsons and Foley 2013; reviewed in Ratheesh *et al.* (2015)]. However, this conclusion may have to be interpreted in the context of an additional proposed antiapoptotic function of Pvr in embryonic blood cells. Apoptosis seen in *Pvr^1^* mutant embryos can be reversed through expression of the viral caspase inhibitor p35 and this inhibition also restores hemocyte migration (Brückner *et al.* 2004; Parsons and Foley 2013). By late stage 14, plasmatocytes are evenly distributed throughout the embryo, with the exception of dense clusters around the head, foregut, and hindgut (Tepass *et al.* 1994). In contrast, stage 17 embryos have crystal cells clustered around the proventriculus (Lebestky *et al.* 2000).

### Sessile pools and circulating larval hemocytes

#### Summary:

**Figure 3:**
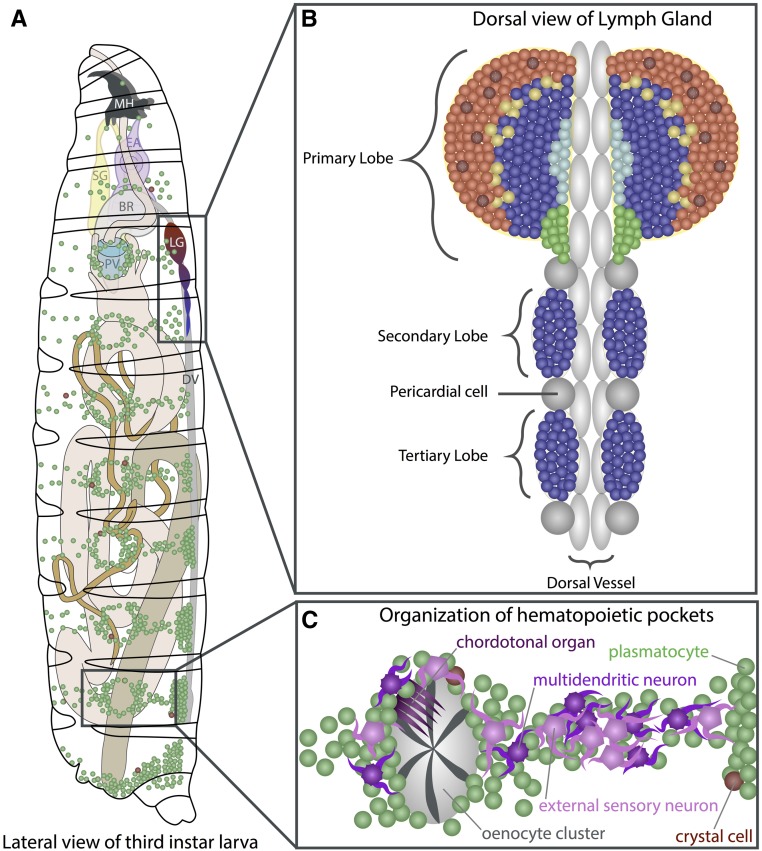
Larval hematopoiesis. (A) In the third-instar larva, the LG lobes are positioned spanning the DV (gray) posterior to the BR. Plasmatocytes (green) and crystal cells (red) circulate in the hemolymph throughout the larva and are also seen in segmentally distributed sessile pools. (B) The lobes of the LG flank the DV. The primary lobes are the largest and most anterior within the LG, and consist of several distinct cell types and zones (see Figure 4). The posterior lobes (blue) are smaller and remain largely undifferentiated. Pericardial cells (gray spheres) separate the individual lobes of the LG. (C) Detailed view of sessile hematopoietic pockets. The majority of sessile hemocytes, including plasmatocytes and crystal cells, reside along the dorsal side of the larva in stereotypically arranged lateral patches termed hematopoietic pockets. Clusters of oenocytes (gray) also reside within these regions, but are dispensable for the formation of the sessile pools. Activity of external sensory and multidendritic neurons (purple) is necessary for adherence, proliferation, and maintenance of hemocytes within hematopoietic pockets. Schematic in (A) adapted from Volker Hartenstein, in (C) adapted from Gold and Brueckner (2015). BR, brain; DV, dorsal vessel; EA, eye-antennal disc; LG, lymph gland; MH, mouth hooks; PV, proventriculus; SG, salivary gland.

During the larval instars, hemocytes derived from the head mesoderm during embryogenesis spread throughout the animal and are found in two locations: a subset of them circulates in the hemolymph and the rest are attached to the body wall in sessile pools (Shrestha and Gateff 1982; Lanot *et al.* 2001) (Figure 3A). Unlike circulating hemocytes, which are readily released upon bleeding, release of sessile hemocytes requires physical disruption by pressure applied to the larval cuticle. The sessile hemocytes are secluded from the open hemocoel, and are positioned in between the epidermal and muscle layers of the larva in clusters of cells termed epidermal–muscular pockets (Makhijani *et al.* 2011) (Figure 3C). These cells initially form a pattern of lateral patches that later extend into dorsal stripes. Formation of this pattern is dependent upon hemocytes homing to the pockets, followed by adhesion and lateral migration. Peripheral neurons that innervate these pockets are required for hemocyte homing to the sites and provide Activin-β/TGF-β (Transforming Growth Factor) signals to promote adhesion and proliferation (Makhijani *et al.* 2011, 2017). Both Rac1 GTPase and the Jun N-terminal kinase (JNK; Basket, Bsk) are required for proper adhesion and targeting of hemocytes to these sessile pools (Williams *et al.* 2006). The Nimrod family transmembrane receptor Eater is also required for hemocyte attachment to the sessile compartment (Bretscher *et al.* 2015). The final step of lateral migration is mediated by Rho1 and the actin cytoskeleton (Makhijani *et al.* 2011). Following external mechanical disruption, the sessile pools disperse but spontaneously reform after 30–60 min, presumably through the same mechanisms that regulate initial pattern formation. Peripheral neurons that innervate these pockets have been proposed to act as a niche to help control hematopoiesis in this tissue through the inhibition of apoptosis and maintenance of sessile hemocytes (Makhijani *et al.* 2011). Ecdysone signaling induces dispersal and activation of sessile hemocytes upon pupariation, and this facilitates tissue remodeling during metamorphosis (Regan *et al.* 2013).

Mature plasmatocytes transdifferentiate into crystal cells within these sessile pools, a process mediated by a Notch-dependent signaling mechanism (Leitão and Sucena 2015). The number of crystal cells in the sessile pools increases by means of transdifferentiation of mature Hml^+^ Lz^−^ plasmatocytes, initially into Hml^+^ Lz^+^ cells that continue to be P1^+^ and retain some phagocytic activity. However, as they mature and express higher levels of Lz, they lose this phagocytic potential and also lose all plasmatocyte markers to become mature crystal cells. During this transdifferentiation process, Hml^+^ Lz^−^ plasmatocytes signal through Notch to physically adjacent cells that are to become Hml^+^ Lz^+^, likely mediated by the ligand Serrate. This process requires cell-to-cell contact, which is achievable in sessile pools, but not when these pools are repeatedly disrupted by mechanical means. The result of such a manipulation is a decrease in the overall number of circulating crystal cells (Leitão and Sucena 2015). Mature plasmatocytes within sessile pools can also form lamellocytes upon wasp infestation (Honti *et al.* 2009). Therefore, although *de novo* conversion of a mesodermal precursor to blood tissue does not seem to occur within the sessile pools, the sessile hemocyte pool is a hematopoietic compartment that contributes to differentiation of hemocytes during larval development as well as in response to immune challenge.

Plasmatocytes can also transdifferentiate into lamellocytes, although the mechanism is not yet fully clear (Avet-Rochex *et al.* 2010; Honti *et al.* 2010; Stofanko *et al.* 2010; Anderl *et al.* 2016). Knockdown of *ush* causes plasmatocytes to transdifferentiate into lamellocytes even in the absence of wasp infestation (Avet-Rochex *et al.* 2010). In addition, overexpression of Srp, *Vinculin-RNAi* (RNA interference), or a dominant negative form of ecdysone receptor induces the formation of lamellocytes in nonparasitized larvae that simultaneously express the lamellocyte marker L1 and the plasmatocyte marker Eater, suggesting a plasmatocyte–lamellocyte conversion (Kroeger *et al.* 2012). However, transdifferentiation of plasmatocytes is unlikely to be the exclusive mechanism for lamellocyte formation, particularly in the lymph gland where lamellocytes can arise directly from a prohemocyte pool in response to wasp infestation (Makki *et al.* 2010; Oyallon *et al.* 2016).

#### Specifics:

In second-instar larvae, the sessile hemocytes are primarily located at the posterior end. In the third instar, they continue to remain as two large clumps of 100–200 hemocytes on segments A8 and A9, forming an organ-like structure termed the posterior hematopoietic tissue (Kurucz *et al.* 2007b; Markus *et al.* 2009). This concentration of hemocytes, which appears early in development, is in addition to the segmentally distributed sessile cells in the epidermal–muscular pockets that are most apparent in the third instar (Makhijani *et al.* 2011). Within the sessile pools, the hemocytes are densely packed and form stable cell-to-cell contacts, and these cells constitute at least a one-third of all larval hemocytes (Lanot *et al.* 2001).

The majority of circulating and sessile larval hemocytes are derived directly from Pxn^+^ cells formed during embryonic hematopoiesis and not from a pool of undifferentiated lymph gland prohemocytes (Makhijani *et al.* 2011). The circulating hemocytes do proliferate, but the sessile hemocytes incorporate EdU at a much higher rate (Makhijani *et al.* 2011; Anderl *et al.* 2016). Overall, the sessile pool is dynamic in its hematopoietic activity and has therefore been thought to function as a compartment that exchanges with the circulating cells, but is independently regulated. Large hepatocyte-like cells called oenocytes, as well as peripheral neurons, reside in or near these hematopoietic pockets, but it is only the peripheral neurons that are required for proper sessile hemocyte cluster formation (Lanot *et al.* 2001; Makhijani *et al.* 2011) (Figure 3C). When the peripheral nervous system (PNS) is perturbed using *atonal* (*ato^1^*) mutants or if the neurons are ablated with diphtheria toxin, the number and pattern of sessile hemocyte clusters is severely altered. Furthermore, hemocytes can be recruited to ectopic sites by misexpression of the proneural gene *scute* (*sc*), which creates supernumerary peripheral neurons (Makhijani *et al.* 2011). The multidendritic sensory neurons and chordotonal organs of the PNS express Activin-β, a ligand for the TGF-β family, which promotes the adhesion and proliferation of hemocytes within the hematopoietic pockets (Makhijani *et al.* 2017). A functional connection between the PNS and the hematopoietic system might have been conserved in vertebrates, since signals from the sympathetic nervous system help regulate HSC proliferation and egress from the bone marrow (Hanoun *et al.* 2015).

Several classes of mutants that either increase the number of circulating hemocytes, affect the dorsal sessile compartments, or induce the spreading of sessile hemocytes throughout the cuticle were identified in an overexpression screen for candidate genes. For example, overexpression of the αPS3 integrin Scab in Pxn^+^ cells disrupts the dorsal sessile compartments, and decreases circulating and lymph gland hemocyte numbers, but also results in hemocyte accumulation on the dorsal vessel. Similarly, overexpression of Kruppel or the CBP homolog Nejire disrupts sessile hemocyte compartments, but surprisingly also induces lamellocyte formation (Stofanko *et al.* 2008). A correlation between lamellocyte formation and release from sessile pools is also seen when Wnt/Wg signaling is disrupted by overexpression of Shaggy (Sgg) or a dominant negative form of Pangolin (Pan)/T-cell factor (TCF), and also upon wasp parasitization (Zettervall *et al.* 2004). In response to wasp infestation, hemocytes in these sessile and circulating pools differentiate into lamellocytes. During this process, the circulating hemocyte population also increases as the sessile pool is released into the hemolymph (Honti *et al.* 2009). Constitutive activation of Toll signaling in *Toll^10B^* mutants also disrupts the sessile hemocyte pools, a phenotype that is suppressed in an *ird1* mutant (Schmid *et al.* 2016).

In *eater* null mutant larvae, both plasmatocytes and crystal cells are virtually absent in the sessile pockets, which results in an apparent increase in the number of circulating hemocytes. Specific knockdown of *eater* in the plasmatocyte lineage using *Hml-GAL4* disrupts plasmatocyte adhesion, which nonautonomously impedes crystal cell attachment to the sessile compartment. This effect is not observed when *eater* is depleted in crystal cells using *lz-GAL4* (Bretscher *et al.* 2015). These experiments illustrate that the sessile plasmatocytes provide an instructive cue for crystal cells to adhere to the sessile compartment, in addition to the Serrate-dependent cue required for transdifferentiation of crystal cells (Bretscher *et al.* 2015; Leitão and Sucena 2015).

Release of hemocytes from the sessile compartments is controlled by several pathways. For example, overexpression of wild-type Rac1 disrupts the sessile population and increases the number of circulating cells (Zettervall *et al.* 2004). Rac1 GTPase requires both JNK activation and actin polymerization to release sessile hemocytes (Williams *et al.* 2006). Ecdysone signaling is another example of a pathway involved in sessile hemocyte release. An ecdysone pulse that occurs at the onset of pupariation is received by hemocytes, resulting in changes in hemocyte morphology, migration, and dispersal, all of which are disrupted upon expression of a dominant negative form of an ecdysone receptor (EcRB1^DN^) in hemocytes. EcR can transcriptionally activate several genes involved in Rac GTPase-mediated actin remodeling, which likely contributes to its effect on hemocyte dispersal during pupariation (Regan *et al.* 2013).

### Dorsal mesoderm

#### Summary:

During the second wave of embryonic hematopoiesis, a region of the dorsal mesoderm called the cardiogenic mesoderm gives rise to both the lymph gland and the dorsal vessel (Rugendorff *et al.* 1994) (Figure 2C and Figure 3B). Lymph gland progenitors and cardioblasts are closely related, and clonal analysis provides evidence for the presence of a hemangioblast population consisting of cells, which in a single division gives rise to one cell that differentiates into the dorsal vessel and another that differentiates into blood (Mandal *et al.* 2004). This is reminiscent of the hemangioblast population in vertebrates that constitutes progenitor cells in the aorta–gonad–mesonephros (AGM) mesenchyme, and produces both blood and vascular cells (Medvinsky and Dzierzak 1996). Several additional molecular and developmental similarities have been noted between these two systems [reviewed in Evans *et al.* (2003)].

Precursors of the lymph gland appear as a local bulge within the cardiogenic mesoderm during stage 13 of embryonic development (Rugendorff *et al.* 1994; Holz *et al.* 2003). These precursors then migrate dorsally to form a tight cluster associated with the dorsal vessel and eventually form a paired chain comprising multiple lobes flanking the dorsal vessel. Cell clusters positive for the zinc finger protein Odd-skipped (Odd) in the three thoracic segments, T1–T3, coalesce to form the lymph gland, while Odd^+^ clusters in the abdominal segments form pericardial cells (Mandal *et al.* 2004). By stages 11–12, mesodermal expression of the homeotic gene *Antennapedia* (*Antp*) is restricted to the T3 segment. This Antp expression is further restricted to 5–6 cells at the posterior boundary of the lymph gland as these cell clusters coalesce during stages 13–16. Antp^+^ cells are the first to proliferate within the larval lymph gland, giving rise to a population of ∼30 cells that have been named the posterior signaling center (PSC) (Mandal *et al.* 2007). These cells provide signals that control the development of the rest of the lymph gland and also participate in the larval response to wasp parasitization. Antp is maintained in the PSC throughout larval development, similar to the expression pattern of the *Drosophila* early B-cell factor (EBF) ortholog, Collier/Knot (Crozatier *et al.* 2004). The rest of the lymph gland cells that form the primary lobes develop from the Odd^+^ clusters that arise from segments T1–T2. The homeodomain cofactor Homothorax (Hth) is initially expressed throughout the embryonic lymph gland and is later downregulated within the PSC. Antp and Hth function in a mutually antagonistic manner, with Antp specifying the PSC and Hth specifying the blood primordium (Mandal *et al.* 2007).

The cardiogenic mesoderm is a subcompartment of the dorsal mesoderm and therefore multiple factors that control dorsal mesoderm formation are also critical for lymph gland development. Examples include BMP/Dpp (bone morphogenetic protein/Decapentaplegic) and FGFR/Heartless (Htl), which control expression of the homeodomain transcription factor Tinman (Tin) and the GATA factor Pannier (Pnr). In addition, Wingless (Wg/Wnt1) positively regulates cardiogenic mesoderm specification and Notch negatively regulates it. Mutations in any of these entities—*dpp*, *htl*, *tin*, *pnr*, or *wg*—cause loss of lymph gland and other associated structures derived from the cardiogenic mesoderm. In contrast, loss of Notch has the opposite effect with substantially more cells arising within the cardiogenic mesoderm (Mandal *et al.* 2004).

#### Specifics:

Following gastrulation and during stages 6–9, the mesodermal cells are not committed to any particular lineage and express mixed markers such as Tin, required for heart development, and Mef2, which regulates muscle formation. These genes are controlled by the mesoderm determinants Twist (Twi) and Snail (Sna) (Bodmer *et al.* 1990; Leptin 1991; Lilly *et al.* 1994; Taylor *et al.* 1995; Yin *et al.* 1997; Cripps *et al.* 1998; Nguyen and Xu 1998). Later in development, the lineage-specific genes will become restricted in their expression, controlled by signals from the segmented ectoderm.

At stage 11, the mesoderm splits into the various lineages that will give rise to the organs derived from them [Eastham 1930; Bate and Rushton 1993; Borkowski *et al.* 1995; Riechmann *et al.* 1997; reviewed in Hartenstein and Chipman (2015)]. Like the ectoderm, the mesoderm is also segmented into myomeres, further split into A and P domains, and abuts the overlying ectoderm. Each of these segmental units moves the A domain toward the ectoderm and the P domains are pushed inside toward the endoderm. During stage 12 (germ band retraction), the A domains fuse to form a continuously linear primordium that will give rise to somatic muscles, dorsal vessel, the lymph gland, and other associated tissues. The P domains, pushed inside, also fuse and will give rise to the visceral mesoderm as well as the fat body. Maintenance of the A domain requires Wg signaling, while the P domain is maintained by Hh (Azpiazu *et al.* 1996; Park *et al.* 1996). These signals are interpreted in the context of pair-rule transcription factors that form the 14 segmental stripes. Each of these fused metameric structures is then differentially specified along the dorsal/ventral axis to position the formation of various organs. This description holds for the mesoderm in the segmented parts of the embryo. The head mesoderm, from which the circulating and sessile hemocytes are derived, have a very different developmental logic. In fact, hemocytes, but not fat and muscles, are the major derivatives of the head mesoderm [reviewed in Hartenstein and Chipman (2015)].

Dpp expressed in the ectoderm specifies the fate of the dorsal mesoderm that will give rise to dorsal vessel/lymph gland (anteriorly) and visceral muscles (posteriorly) in each segment. The ventral part will give rise to somatic mesoderm (A) and fat body (P). Loss-of-function mutations in *dpp* lack the dorsal vessel while overexpression causes heart cells to form from ventral cells (Frasch 1995). The transcription factor Tin is a direct target of the Dpp signal, and its expression also requires the function of FGFR (Shishido *et al.* 1997; Zaffran *et al.* 2002). At this stage of development, expression of Tin defines the region that is designated dorsal mesoderm (Bodmer 1993). Within this dorsal mesoderm, the A quadrant, which is high in both Wg and Dpp activity, defines the cardiogenic mesoderm from which the heart, blood, and the pericardial nephrocytes will arise. This bears many similarities to the AGM region in vertebrate definitive hematopoiesis that arises from the lateral plate mesoderm, also in response to BMP and FGF (Marshall *et al.* 2000; Nishikawa *et al.* 2001). Furthermore, cells sharing an immediate common ancestor within the cardiogenic mesoderm can be fated to become either a dorsal vessel or a lymph gland precursor, leading to the designation of such cells as hemangioblasts in comparison with similar cells in mammals, which can become components of either the blood vessel or the hematopoietic system [reviewed in Marshall and Thrasher (2001) and Mandal *et al.* (2004)].

The homeodomain protein Tin, initially expressed broadly in the mesoderm, is later restricted to the cardiogenic mesoderm. Interestingly, Tin is a homolog of the vertebrate Nkx2.5, which is considered a heart-specific marker in both vertebrates and in *Drosophila* (Lien *et al.* 1999). In reality, *Drosophila* Tin is expressed in the common progenitor for both heart and blood cells, and then becomes heart-specific only when these lineages diverge (Mandal *et al.* 2004). The dorsal mesoderm requires Tin and Pnr, both of which are controlled by FGFR and Dpp signaling (Frasch 1995; Beiman *et al.* 1996; Klinedinst and Bodmer 2003). Prior to the specification of lymph gland precursors from cardioblasts at the time of germ band retraction (stages 12–13), the entire cardiogenic mesoderm expresses Tin, but by stage 13, Tin and Pnr become confined to cardioblasts. This refinement is essential for lymph gland specification because ectopic expression of Tin or Pnr throughout the entire mesoderm, or in the cardiogenic mesoderm, reduces the numbers of both lymph gland and pericardial cells. Odd continues to be expressed throughout the cardiogenic mesoderm while Srp is upregulated in lymph gland precursors (Mandal *et al.* 2004).

Notch is active during stages 11–13 and plays a dual role in lymph gland specification. At stage 11/12, Notch is required for specification of the cardiogenic mesoderm, while during stages 12/13 Notch inhibits expression of Tin and upregulates Odd and Srp in a Delta-dependent manner (Mandal *et al.* 2004; Grigorian *et al.* 2011a). Consequently, during stages 12/13, reduction of Notch causes an increased number of cardioblasts at the expense of lymph gland precursors, while expression of activated Notch (N^act^) gives rise to a larger lymph gland (Mandal *et al.* 2004). Null mutations in *Delta* convert all cells of the cardiogenic mesoderm into cardioblasts (Grigorian *et al.* 2011a). Delta ligand expression is widespread until stage 12, but then becomes spatially restricted to cardioblasts and persists through stage 14. EGFR and FGFR are also required for specification and maintenance of the cardiogenic mesoderm, since expression of a constitutively active form of Ras in these cells increases their numbers (Grigorian *et al.* 2011a).

Following the split of the cardioblast and lymph gland lineages, the pericardial cells are distinguished from lymph gland precursors through regulation by Srp. In *srp* null embryos, Odd^+^ cells still form a cluster resembling the early lymph gland; however, these Odd^+^ cells now express the pericardial marker Pericardin. On the other hand, Srp expression throughout the cardiogenic mesoderm induces pericardial cells to adopt the lymph gland fate (Mandal *et al.* 2004).

Tin, Pnr, and Srp control the conserved basic helix-loop-helix transcription factor Hand, which is critical for both heart and lymph gland development. Hand expression is initiated in the cardiogenic region in late stage 12 and, while it is expressed in cardioblasts, pericardial cells, and lymph gland precursors, it is regulated differently in these cell types. In cardioblasts and pericardial cells, Tin and Pnr control Hand expression, while in lymph gland precursors, Srp controls Hand expression (Han and Olson 2005). *Hand* null mutant embryos and larvae exhibit complete loss of lymph gland, pericardial cell, and cardiac precursors through apoptosis. Thus, it is likely that a primary function of Hand is to promote cell survival in the cardiogenic mesoderm (Han *et al.* 2006).

Cells of the lymph gland primary lobe that will eventually give rise to hemocytes arise from embryonic segments T1–T2, while the cells of the PSC arise from 5–6 Antp^+^ cells that originate from the T3 segment. The expression of Antp is maintained in the PSC throughout larval development (Mandal *et al.* 2007). Collier is expressed in two clusters of cells in T2 and T3 that later coalesce. Following germ band retraction, Collier expression remains high in 3–5 cells at the P tip of the lymph gland and at low levels throughout the rest of the lymph gland cells (Crozatier *et al.* 2004). The PSC cells initially form in *collier* mutants but are lost by the third instar, indicating that Collier is specifically required for PSC maintenance and not for its specification (Crozatier *et al.* 2004). Collier expression is seen prior to, and is independent of, Srp as it continues to be expressed in *srp^6G^* mutant embryos. However, Collier expression in the PSC depends on Antp and is not maintained in *Antp* mutant embryos. In contrast, loss of *collier* does not affect initial Antp expression (Mandal *et al.* 2007).

In addition to T1–T3, Odd is also expressed in the abdominal segments A1–A6 in stage 11 embryos (Ward and Skeath 2000). These latter clusters form pericardial cells (Rugendorff *et al.* 1994; Holz *et al.* 2003; Mandal *et al.* 2007). Positional cues provided by the homeobox protein, Ultrabithorax (Ubx), in segments A1–A5, restricts primary lobe lymph gland formation to the thoracic regions of the cardiogenic mesoderm. Loss of Ubx results in abnormal expansion of lymph gland cells into the abdominal segments [Rodriguez *et al.* 1996; Mandal *et al.* 2004; reviewed in Hartenstein and Chipman (2015)].

Later in development, following dorsal closure (stage 17), the rows of cardioblasts on either side of the embryo come together and fuse. The dorsal vessel forms as a double row of cardioblasts lining a central lumen through which the hemolymph circulates (Rugendorff *et al.* 1994). In the mature *Drosophila* embryo, the lymph gland appears as a paired cluster of ∼20 cells flanking the anterior region of the dorsal vessel (Mandal *et al.* 2004). The cells of the lymph gland continue to express both Srp and Odd throughout larval development (Jung *et al.* 2005).

Based on the nomenclature used for the larval heart, the wide, posterior part of the dorsal vessel is referred to as the heart, and the anterior part as the aorta (Miller 1950; Rizki 1978). The hemolymph is pumped through the lumen of the dorsal vessel in the P→A direction, exiting the aorta along with the circulating hemocytes, which are released at the anterior end into open circulation within the body cavity. In addition to the incurrent opening at the posterior end of the heart through which the majority of the blood enters, the rest enters through small, segmentally distributed lateral openings or ostia along the length of the heart. The aorta part of the dorsal vessel does not have these ostia and maintains unidirectional flow. Together, the aorta and heart represent a contractile tube lined by a layer of myoepithelial vascular cardioblasts. Alary muscles connect the dorsal vessel to the epidermis. Encircling the anterior tip of the dorsal vessel is the ring gland, an organ involved in maintaining a complex endocrine system, including steroid hormones that maintain growth cycles of the developing larva and pupa. Along the edge of the dorsal vessel and posterior to the ring gland are the primary lobes of the lymph gland (Figure 3B). This is followed by a series of smaller lobes that flank the dorsal vessel (Rugendorff *et al.* 1994). In between the major lobes, and also in a loose row posterior to the lymph gland on either side of the dorsal vessel, are large, nonpolarized pericardial nephrocytes. Although collectively termed pericardial cells, they are not all derived from identical precursors (Zmojdzian *et al.* 2018). Similar to vertebrate nephrocytes, mature pericardial cells have extensive arrays of folding membranes to provide greater surface area, and are involved in the ultrafiltration and excretion of hemolymph (Weavers *et al.* 2009). While all structures described above originate from the cardiogenic mesoderm, it is only the lymph gland lobes where hematopoietic progenitors reside.

## Larval Lymph Gland: Zones, Cells, and Signals

### Summary

**Figure 4:**
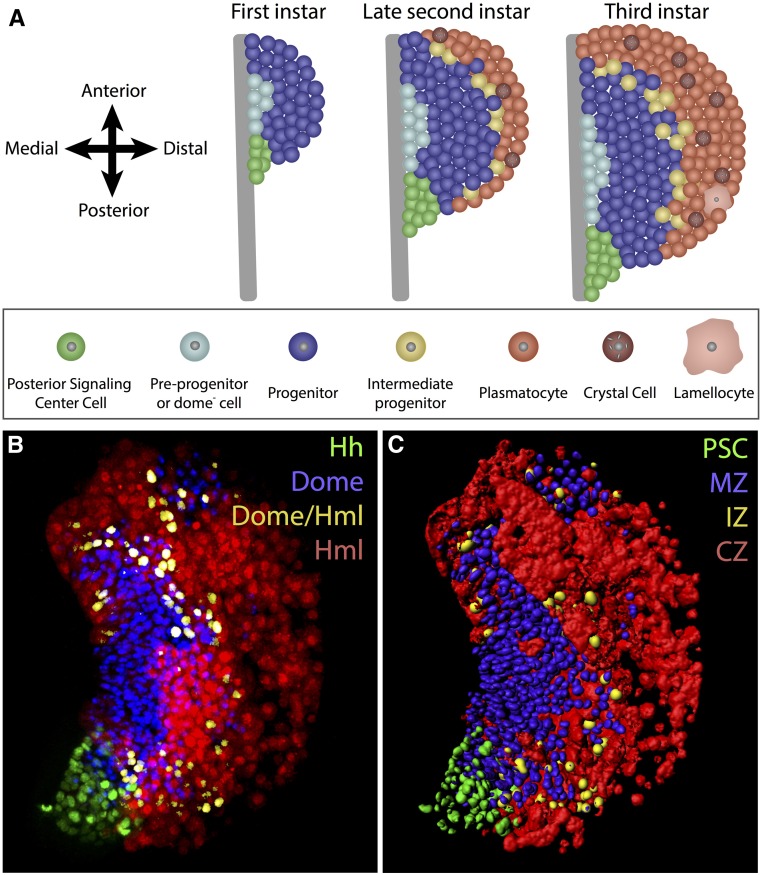
LG zones and cell types in the primary lobe. (A) Schematic diagrams of LG primary lobes from first (left), late second (middle), and third (right) instar larvae. By convention, we designate the region close to the dorsal vessel as medial and the opposite edge as distal. Anterior is up in all LG images. The PSC (green) acts as a niche for LG progenitors and is present throughout development in all three instars. The majority of cells in the first-instar primary lobe are undifferentiated Domeless^+^ progenitors that belong to the MZ (dark blue). A small number of *domeless*^−^ and Notch^+^ preprohemocytes (light blue) lie on the medial edge of the lobe adjacent to the dorsal vessel (gray), and are capable of contributing to the MZ population during the first 5–20 hr of first instar development. The second-instar LG develops mature hemocytes that make up the CZ (red). This zone mostly includes plasmatocytes and a small number of crystal cells. Additionally, an IZ (yellow) at the interface of the MZ and the CZ contains cells that express both progenitor (*domeless*) and differentiating hemocyte (*Peroxidasin* and *Hemolectin*) markers, but they lack mature hemocyte markers such as P1 or Lozenge/Hindsight. A population of *domeless*^−^ cells remains along the medial edge of the lobe, although they no longer retain active Notch signaling. During the third instar, the overall LG size increases and a larger number of differentiated hemocytes are seen. (B) Image of a third-instar primary lobe obtained using fluorescence microscopy. *Hh-GFP* (PSC; green), *domeMESO-BFP* (MZ; blue), *Hml-DsRed* (CZ; red), and intermediate progenitors (pseudocolored as yellow based on overlap of *domeMESO-BFP* and *Hml-DsRed*). (C) Computer rendering of the confocal data shown in (B) using Imaris software. This software generates accurate three-dimensional models from which quantitative data can be readily derived. CZ, cortical zone; Hh, Hedhehog; Hml, Hemolectin; IZ, intermediate zone; LG, lymph gland; MZ, medullary zone; PSC, posterior signaling center.

Structurally, the larval lymph gland is divided into multiple lobes. The anterior-most lobe, also called the primary lobe, is the best characterized (Figure 3B). Located posterior to the primary lobe are the secondary, tertiary, and quaternary lobes, which are much smaller in size and in the absence of immune challenge exhibit significantly lower levels of hematopoietic activity compared to the primary lobes. Based on extensive morphological and molecular marker analysis, the primary lobe of the lymph gland can be divided into zones, each containing cells that are functionally distinct. These zones include the PSC, which acts as a niche to regulate progenitor maintenance; the Medullary Zone (MZ) consisting of medially located hemocyte progenitors; and the distal Cortical Zone (CZ), which is populated by maturing hemocytes (Jung *et al.* 2005) (Figure 4). This subdivision has revealed an underlying spatial and temporal regulation of blood cell development within the primary lobe throughout the larval stages. Technically, the first zone to arise as a separate cell population is the PSC since it can be distinguished as a separate cluster of cells in the embryo, as described above. In larval development, the PSC is located at the posterior cusp of the primary lobe, adjacent to a single pericardial cell that separates it from the secondary lobe. Akin to the purely signaling function ascribed to the stroma in mammalian development, clonal analysis demonstrates that the cells of the PSC do not give rise to any progenitor or differentiated blood cell types, but do provide signals to regulate progenitor maintenance or differentiation (Crozatier *et al.* 2004; Krzemień *et al.* 2007; Mandal *et al.* 2007; Mondal *et al.* 2011; Benmimoun *et al.* 2012; Pennetier *et al.* 2012; Tokusumi *et al.* 2012; Khadilkar *et al.* 2014, 2017; Dey *et al.* 2016). Prior to the midsecond instar, the cells within the lymph gland primary lobe are progenitors that express a reporter for *domeless* (*dome*). These Dome^+^ progenitors will populate the MZ and differentiate at their distal edge to initiate formation of the CZ (Jung *et al.* 2005; Evans *et al.* 2009; Krzemien *et al.* 2010; Minakhina and Steward 2010; Dey *et al.* 2016) (Figure 4). As yet, there is no direct evidence that these Dome^+^ cells self-renew while simultaneously producing a differentiated offspring, as is evident during germline differentiation (Fuller and Spradling 2007).

### Specifics

The first- and early second-instar lymph glands are largely populated by progenitor cells that express Dome, the receptor upstream of JAK/STAT (Jung *et al.* 2005; Dey *et al.* 2016). During these early stages, the primary lymph gland lobe has a smooth appearance and consists of a rapidly dividing population of Dome^+^ progenitors that are tightly packed with close cell-to-cell contacts (Jung *et al.* 2005) (Figure 4A). In the first larval instar, a small population of cells on the medial side of the lymph gland, along the dorsal vessel and perhaps extending adjacent to the PSC, do not express Dome as the more distal cells do. These cells are preprogenitors and were named as such based on marker expression (Jung *et al.* 2005). In more current literature, genetic and molecular analyses have sought to determine the nature of this cell population, and have found that the preprogenitors give rise to the Dome^+^ cells of the MZ (Minakhina and Steward 2010; Dey *et al.* 2016).

Dome^+^ progenitors attenuate their rate of proliferation during the midsecond instar as they change in overall density. Cells at the periphery begin to increase their spacing and granularity coincident with loss of E-cad (encoded by *shotgun*) as they initiate differentiation. It is from this point on in development that the separate zones are readily identifiable within the primary lobe. In addition to morphological criteria, the zones become distinct based on their marker expression patterns. The PSC cells are distinguished by their expression of Antp, Hh, and Collier (Lebestky *et al.* 2003; Crozatier *et al.* 2004; Mandal *et al.* 2007). The MZ expresses high levels of *dome* and *upd3* reporters, ROS, Wg, E-cad, and very low levels of Collier (Hombría *et al.* 2005; Jung *et al.* 2005; Krzemień *et al.* 2007; Owusu-Ansah and Banerjee 2009; Benmimoun *et al.* 2015; Oyallon *et al.* 2016). ECM proteins, including Viking (Vkg), a component of Collagen IV, and the *Drosophila* homolog of mammalian Perlecan (Trol), are densely interwoven between individual cells of the MZ and are sparser in surrounding groups of differentiated cells of the CZ (Krzemien *et al.* 2010; Grigorian *et al.* 2013). Plasmatocytes within the CZ are identified by Hml, Eater, the NimC antigen P1, and Peroxidasin (Pxn) (Jung *et al.* 2005; Kurucz *et al.* 2007a,b; Stofanko *et al.* 2008; Sinenko *et al.* 2009; Makhijani *et al.* 2011). Crystal cells are identified by the expression of Lozenge (Lz), Hindsight (Hnt), Sima (*Drosophila* Hif-α), prophenoloxidases (PPO1 and PPO2), and by the *Bc* mutation/reporter (Rizki *et al.* 1980; Lebestky *et al.* 2000; Jung *et al.* 2005; Tokusumi *et al.* 2009a; Mukherjee *et al.* 2011; Binggeli *et al.* 2014). In the rare instance that lamellocytes are detected within lymph glands of nonparasitized larvae, they are easily identified as giant cells within the CZ that express L1/Atilla, Misshapen, α-PS4 integrin, and its partner Myospheroid (Braun *et al.* 1997; Crozatier *et al.* 2004; Irving *et al.* 2005; Kurucz *et al.* 2007b; Honti *et al.* 2009; Tokusumi *et al.* 2009b). The individual zones and the patterns of gene expression of cells occupying them remain distinguishable through the late third instar, becoming less so as the lymph gland begins to dissociate during early pupal stages (Grigorian *et al.* 2011b).

Positioned between the progenitors of the Dome^+^ MZ and the differentiating cells of the Pxn^+^ CZ, there is a small population of cells that simultaneously express markers for both zones. These were initially described as being in a “transition state” as these cells are both Dome^+^ and Pxn^+^ (Sinenko *et al.* 2009). Simultaneous expression of dominant negative forms of Fz and Fz2 increases the number of these transitioning cells (Sinenko *et al.* 2009). More recent literature has referred to these cells as belonging to a separate zone termed the Intermediate Zone (IZ) (Krzemien *et al.* 2010) (Figure 4). This nomenclature is now widely used (including in this review) and the cells therein are referred to as intermediate progenitors. While these cells are positive for the earliest differentiation markers Hml and Pxn, they lack later, mature markers for plasmatocytes (P1) and crystal cells (Lz) (Krzemien *et al.* 2010). This intermediate progenitor population is not yet fully characterized, but given that cells within the IZ transit from a relatively quiescent multipotent state to one in which their fate is specified, this zone is likely quite important, and deserving of a more comprehensive and mechanistic characterization of pathways governing their fate (Kalamarz *et al.* 2012; Kroeger *et al.* 2012).

### Preprogenitors

#### Summary:

A small group of cells located at the most medial portion of the lymph gland near the dorsal vessel just above the PSC are Dome^−^ and were designated preprogenitors (Jung *et al.* 2005) (Figure 4A). Clones generated in the lymph gland during embryogenesis and the first larval instar were analyzed to determine if large single clones, such as those arising from stem cells, can form during development (Minakhina and Steward 2010). The authors report that clones generated near the PSC can occupy up to one-third of the lymph gland and are maintained in position near the PSC. The authors suggest that the size of these clones is dependent on the Zfrp8 protein, and that cells derived from these clones are competent to become either plasmatocytes or crystal cells. Clones distant from the PSC are small and scattered (Minakhina and Steward 2010). Large clones are also induced from the region of the Dome*^−^* preprogenitors, adjacent to the dorsal vessel. These Dome^−^ preprogenitors are seen 5–22 hr after hatching and have been termed HSCs based on this clonal analysis (Dey *et al.* 2016). As stated earlier, the widely recognized vertebrate term HSC is associated with additional characteristics not yet established in *Drosophila*, and while these results are consistent with a stem-like identity for the Dome^−^ cells, given the very transient nature of this population, it is preferable to retain their designation as preprogenitors until future studies settle the issue of self-renewal linked to asymmetric cell division within this population.

#### Specifics:

About 4–5 Dome^−^ cells are seen 8 hr after egg hatching in first-instar larvae. They are larger in size than their Dome^+^ neighbors and express low levels of Dorothy (Dot) (Dey *et al.* 2016). Importantly, these cells also express several indicators of active Notch pathway signaling including *Notch-GAL4*, *Su(H)-lacZ*, and a target gene for the pathway, *Enhancer of split m*β (*E(spl)m*β). In addition to the Notch pathway reporters, Trio, a guanyl-nucleotide exchange factor previously implicated in stem cell maintenance in mammals, is also expressed in these cells. A majority of these Notch^+^ cells are in S phase and, as they progress through G2 and mitosis, they transition to become Dome^+^ progenitors by 22 hr after egg hatching during the late first larval instar. Clonal analysis shows that these Notch^+^ preprogenitors are multipotent and contribute to the Dome^+^ population (Dey *et al.* 2016).

**Figure 5:**
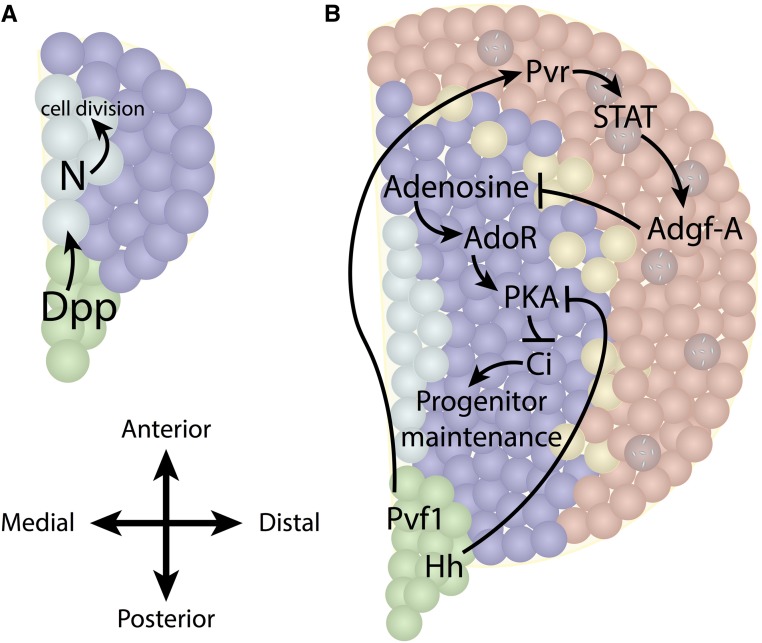
Interzonal signaling. (A) During the first instar, a Dpp signal originates from the PSC and activates the Notch pathway by an unknown mechanism in 5–8 preprogenitor cells along the medial edge of the primary lobe. The preprogenitors are *domeless*^−^, proliferate, and give rise to the Domeless^+^ progenitors of the MZ. (B) Late second instar onward, the PSC secretes both Hh and Pvf1, which are needed to maintain progenitor quiescence. The niche-derived Hh signal is important for generating an active version of Ci (Ci^ACT^) in the MZ. The Pvf1-derived signal is sensed by the cells of the CZ, which trigger a molecular cascade known as the equilibrium signal that also further enhances the stability of Ci^ACT^ in the MZ. The combination of the niche-derived Hh signal and the equilibrium signal arising from newly differentiated cells together maintain a progenitor cell population that is adaptable to both homeostatic and stress-induced conditions. All arrows represent genetic regulation and not necessarily direct molecular steps within a transduction cascade. Adgf-A, Adenosine deaminase growth factor-A; AdoR, adenosine receptor; Ci, Cubitus interruptus; CZ, cortical zone; Dpp, Decapentaplegic; Hh, Hedhehog; MZ, medullary zone; PSC, posterior signaling center; Pvr, PDGF/VEGF receptor.

This preprogenitor fate is also dependent upon Dpp signaling from the niche (Figure 5A). Phosphorylated Mothers against dpp (pMad) is elevated within these preprogenitors and is critical for their Notch-induced E(spl)mβ expression. Knockdown of *dpp* in the PSC or depletion of Mad in the Notch^+^ preprogenitors causes a threefold decrease in lymph gland size at third instar, consistent with a role of Notch in preprogenitor proliferation. This population of Dome^−^ and Notch-pathway active cells is not seen after the first instar, and thus far represents the earliest postembryonic hematopoietic preprogenitor population within the lymph gland (Dey *et al.* 2016).

Later in the second and third instars, Dome^−^ lymph gland cells neighboring the dorsal vessel express a GFP reporter for the TEAD family transcription factor Scalloped (Sd) (Ferguson and Martinez-Agosto 2017). Pvf2, one of the three ligands known to bind and activate the PDGF/VEGF receptor Pvr, is dependent on Sd and expressed in cells positive for this transcription factor. An RNAi knockdown of *Pvf2* specifically within these cells, but not in the Dome^+^ cells, causes an early proliferation defect resulting in a small lymph gland. Overexpression of Pvf2 in these cells rescues the lymph gland growth defect of *sd* hypomorphic mutant animals. These data suggest that Pvf2 expression is essential for normal lymph gland proliferation during early larval stages (Ferguson and Martinez-Agosto 2017). Although these Dome^−^ Sd^+^ cells are located in a similar region to the Dome^−^ Notch^+^ cells seen in the first instar (Dey *et al.* 2016), and loss of either population affects the total number of cells in the mature lymph gland, further experimentation is needed to establish the relationship between these two populations to fully understand the progenitor hierarchy during blood cell development.

### Progenitors and differentiation: the MZ and CZ

The MZ cells are the common progenitors for all mature blood cell types (Jung *et al.* 2005; Defaye *et al.* 2009; Krzemien *et al.* 2010; Minakhina and Steward 2010; Dey *et al.* 2016). These progenitors are critically dependent on a multitude of signals from three different origins to retain their quiescence and multipotency. These are the autonomous/autocrine signals that originate within the zone and are received by the same population, signalling via secreted factors that originate from a different zone within the lymph gland that affect the MZ and, finally, systemic signals that result from active sensing of the internal and external environment.

A truly unique feature of the MZ is the strict control of progenitor proliferation during development. During the first and early second larval instars, all non-PSC cells within the lymph gland, except the small number of preprogenitors described above, are Dome^+^. Bromodeoxyuridine (BrdU) incorporation studies show that during these stages, the Dome^+^ cells proliferate extensively in an asynchronous manner. Then, coincident with the first appearance of differentiated cells during the mid-to-late second instar, proliferation slows dramatically and very few of the cells incorporate BrdU. As development proceeds, and the separation of the MZ and CZ becomes more prominent, the Dome^+^ cells of the MZ continue to maintain a low proliferative state, while the CZ maintains higher proliferative rates throughout the third instar to expand its population (Jung *et al.* 2005; Krzemien *et al.* 2010). This control of proliferation is critical for the MZ cells to remain multipotent and undifferentiated. Progenitor maintenance requires a niche-derived signal, as well as one that emanates from the differentiating cells, termed an “equilibrium signal.” Loss of either signal causes the MZ cells to lose multipotency, as they proliferate and subsequently differentiate (Mandal *et al.* 2007; Mondal *et al.* 2011). An important question to resolve is the mechanism by which the proliferative and fate-determinative events are coupled during hematopoiesis. A reasonable hypothesis to test in the future is that differentiation signals are received by all cells, but are not interpreted as such by cells that are maintained in a nonproliferative state. A relief in the proliferation block at the boundary between the zones would then allow these cells to differentiate. The molecular mechanisms underlying the maintenance and release of progenitors from quiescence is a current topic of investigation within the field.

In the following sections, we elaborate upon some of the key molecular events that control the balance between progenitors and mature hemocytes within the larval lymph gland.

#### ECM:

Differences in the expression of ECM proteins contribute to morphological distinctions between the zones of the lymph gland (Lanot *et al.* 2001; Jung *et al.* 2005; Krzemien *et al.* 2010; Grigorian *et al.* 2013). The progenitor cells of the MZ are densely arranged and express the cell adhesion molecule E-cad (Jung *et al.* 2005). In other systems, the intercellular mechanical tension caused by E-Cad binding across junctions has been shown to lead to reorganization of integrin molecules and the deposition of ECM components (Weber *et al.* 2011). During normal lymph gland development, progenitors maintain their close contact until maturation and dispersal. Loss of E-cad in the progenitors induces differentiation while overexpression promotes enhanced progenitor maintenance (Jung *et al.* 2005; Gao *et al.* 2009, 2013). The CZ expresses high levels of the collagen-binding protein SPARC (secreted protein acidic cysteine-rich) (Irving *et al.* 2005). This is also true of mature embryonic hemocytes, in which SPARC expression is essential for the proper deposition of collagen during the formation of the basal lamina (Martinek *et al.* 2008). A thin layer of collagen encapsulates the outer boundary of the lymph gland lobes, separating the cells from the hemolymph (Lanot *et al.* 2001; Krzemien *et al.* 2010). In addition, the Collagen IV subunits Viking (Vkg) and α1 (Cg25C) are detected in an interwoven pattern throughout the lymph gland, although transcriptional reporters for α1 are detected only in the CZ (Jung *et al.* 2005; Sorrentino *et al.* 2007; Benmimoun *et al.* 2012; Grigorian *et al.* 2013). This may indicate that Collagen IV is secreted by the mature hemocytes then distributed throughout the entire lymph gland. The *Drosophila* homolog of the heparin sulfate proteoglycan Perlecan (encoded by *terribly reduced optic lobes*; Trol) also forms a thin layer that coats the periphery of the lymph gland. Within the lymph gland proper, Trol is highly concentrated between MZ progenitors but also surrounds either individual or groups of cells within the CZ (Grigorian *et al.* 2013). Loss of *trol* function throughout the entire animal leads to a loss of circulating blood cells and a smaller lymph gland (Lindner *et al.* 2007; Grigorian *et al.* 2013). Specific knockdown of *trol* within progenitors, but not CZ cells, leads to premature differentiation and loss of progenitor maintenance (Grigorian *et al.* 2013). Whether additional sources of ECM proteins, such as from the fat body or circulating hemocytes, contribute to the lymph gland is not yet clear. Together, the appropriate distribution of ECM proteins among the zones provides the proper local environment and cell-to-cell interactions for hemocytes to become functionally active while they are still confined within the hematopoietic organ.

#### Hh signaling:

Hh binds to its receptor Patched (Ptc) and consequently activates the downstream transcription factor Cubitus interruptus (Ci^ACT^). In the lymph gland, Hh is expressed in the cells of the PSC in the second and third instar (Figure 5B). In *hh^TS^* mutants or when *hh-RNAi* is driven in the PSC, the specification and maintenance of the PSC are unaffected, as indicated by normal Antp expression. However, both genotypes cause a significant increase in the differentiation of mature hemocytes (Mandal *et al.* 2007; Baldeosingh *et al.* 2018). Expression of Ptc and Ci^ACT^ is elevated within progenitors. Loss of Ci function in the progenitors phenocopies Hh loss-of-function in an otherwise normal PSC and the resulting lymph gland lacks a proper MZ (Mandal *et al.* 2007; Baldeosingh *et al.* 2018). Projections visualized using a membrane-bound form of GFP are seen emanating from the PSC cells and help to extend Hh distribution within the MZ (Krzemień *et al.* 2007; Mandal *et al.* 2007).

#### Wg/Wnt signaling:

Wg/Wnt pathway activity plays a complex role in lymph gland development (Sinenko *et al.* 2009; Zhang *et al.* 2014). An antibody raised against Wg (although it might cross-react with other Wnts; Doumpas *et al.* 2013) detects Wg throughout the lymph gland at early larval stages. This staining, as well as that of one of its receptors, Fz2, is less prominent in the cells of the CZ (Sinenko *et al.* 2009). Differentiating cells that express reporters for *Hml* strongly attenuate Wg staining, and when dominant negative versions of both Fz and Fz2 are simultaneously driven in the MZ, the boundary between the MZ and CZ becomes disorganized. The cell adhesion molecule E-Cad, which glues the MZ cells together, is downregulated when Wg signaling is decreased and this may possibly contribute to the disruption of zones. No obvious change in differentiation markers is evident except for an increase in the number of cells of the IZ displaying both MZ and CZ markers (Sinenko *et al.* 2009). While this could suggest that, in the absence of Wg signaling, cells are unable to escape a transition state, this conclusion is likely an oversimplification since unpublished work from our laboratory suggests that multiple Wnts, from different sources within the lymph gland and following multiple modes of downstream function, influence progenitor maintenance. On the other hand, hyperactivation of the pathway by either overexpression of Wg or a constitutively active form of β-Catenin [Armadillo (Arm)] has a dramatic phenotype in which the MZ expands to fill the entire lymph gland at the cost of differentiated cells, resulting in a virtual loss of the CZ (Sinenko *et al.* 2009). The ECM protein Tig is repressed in the progenitors by Wg signaling in a TCF/Pan- and Arm-dependent manner. Tig can be readily detected within CZ cells and this expression is repressed upon overactivation of the Wg pathway. Lymph glands that lack Tig are overall smaller than wild-type and without Tig, plasmatocyte differentiation occurs early within the primary and posterior lobes (Zhang *et al.* 2014). Overexpression of Tig in Hml^+^ cells does not alter crystal cell and lamellocyte formation, but it does inhibit P1 and Eater expression, and thus plasmatocyte maturation (Zhang and Cadigan 2017).

#### Calcium signaling:

GABA_B_R, a metabotropic receptor for γ-aminobutyric acid (GABA), is expressed in both the PSC and MZ. In the PSC, it plays a role in maintaining lymph gland size without affecting hemocyte differentiation. This regulation occurs early in lymph gland development, before midsecond instar, and is independent of the nonautonomous function of the PSC in progenitor maintenance. Disruption of the calcium signal within the cells of the PSC phenocopies the small lymph gland defect, indicating that the calcium/calmodulin pathway promotes early lymph gland development in a role that is distinct from its function in progenitor maintenance. Within the MZ, calcium signaling is required for the normal maintenance of progenitor cells. Decreased calcium signaling induced by blockage of ER-dependent release of Ca^2+^, reduction of stored ER calcium, or reduction of downstream calcium signaling components all lead to an increase in differentiated cells at the expense of the progenitor population. The opposite effect is seen when the calcium signal is increased relative to wild-type. In these genetic backgrounds, the MZ is larger and the lymph gland contains many fewer mature hemocytes than seen in wild-type (Shim *et al.* 2013).

#### JAK/STAT pathway:

The JAK/STAT pathway, also active within the MZ, helps maintain progenitor identity and prevents differentiation (Jung *et al.* 2005; Krzemień *et al.* 2007; Ferguson and Martinez-Agosto 2017). The receptor Dome, which is highly expressed in progenitors, is able to bind the cytokines Unpaired 1–3 (Upd1–3) to activate downstream gene transcription (Krzemień *et al.* 2007; Makki *et al.* 2010). STAT92e activity is high in the CZ but low in the MZ, as determined by the expression of its downstream target Chinmo in scattered cells within the CZ (Flaherty *et al.* 2010). Loss of STAT activity using a temperature-sensitive mutant indicates that STAT function is required to maintain the MZ population (Krzemień *et al.* 2007). However, loss of Dome, Hop (the *Drosophila* JAK), or STAT specifically within the MZ shows no phenotypic effect on lymph gland differentiation (Minakhina *et al.* 2011; Mondal *et al.* 2011). While basal expression of JAK/STAT pathway components within the progenitors may not be essential for normal development, the pathway plays an important role during the differentiation of progenitors into lamellocytes in response to immune stress. Latran (encoded by *eye transformer*), also expressed within the progenitors, can heterodimerize with Dome and inhibit its function (Makki *et al.* 2010). Although a loss of Latran does not lead to a differentiation phenotype, it does have an effect on lamellocyte formation upon wasp parasitization (Makki *et al.* 2010).

Downstream of JAK/STAT, Ush promotes E-cad and Ptc expression within the MZ to impede differentiation (Gao *et al.* 2009, 2013). Prior to hemocyte differentiation, Ush is expressed in all immature cells and is subsequently downregulated in the maturing cells of the CZ. A single-copy loss of *ush* increases numbers of plasmatocytes and crystal cells, and lowers levels of E-cad and Ptc within the MZ (Gao *et al.* 2009). Biochemical analysis suggests that Ush physically binds Srp to overcome Srp-dependent inhibition of E-cad expression (Gao *et al.* 2013).

Another downstream factor required for inhibition of differentiation in the MZ is the endosomal trafficking protein Asrij (Arj) (Kulkarni *et al.* 2011; Sinha *et al.* 2013). Arj, the *Drosophila* homolog of Ociad1 (Ovarian carcinoma immunoreactive antigen domain-containing 1), a hematopoietic stem cell marker in humans, is present within all hemocytes during embryonic, larval, and adult stages (Phillips *et al.* 2000; Inamdar 2002; Mukhopadhyay *et al.* 2003; Kulkarni *et al.* 2011). Arj is expressed throughout the lymph gland and binds STAT to promote its phosphorylation and activity (Kulkarni *et al.* 2011; Sinha *et al.* 2013). A loss of this protein causes a reduction in the number of MZ and PSC cells, elevated numbers of plasmatocytes and crystal cells in the CZ, and a corresponding decrease in E-cad within the MZ. Loss of Arj also raises the number of larval circulating cells. Interestingly, *arj* mutants also show abnormally high proliferation and crystal cell differentiation in the posterior lobes of the lymph gland, giving rise to a large increase in their size (Kulkarni *et al.* 2011; Sinha *et al.* 2013).

The mammalian homolog of Arj is involved in endosomal trafficking and recycling (Mukhopadhyay *et al.* 2003). Consistent with the possibility that this might be an evolutionarily conserved function, *Drosophila* Arj interacts in significant ways with the Ras family GTPase Arf79F. Loss of Arf79F phenocopies the effects of loss of Arj function, including a smaller PSC size, increased differentiation, loss of progenitors, and large posterior lobes. Both *Arf79F* and *arj* mutant animals display mislocalized Notch^ICD^ protein and a decrease in the ability of hemocytes to uptake fluorescent probes indicative of decreased endocytic function. This aberrant Notch trafficking leads to increased crystal cell differentiation in *arj* mutants (Kulkarni *et al.* 2011; Sinha *et al.* 2013; Khadilkar *et al.* 2014).

#### ROS:

Physiologically generated ROS are seen in a gradient pattern in the lymph gland, with high levels in the MZ gradually fading into lower levels in the differentiated cells of the CZ (Owusu-Ansah and Banerjee 2009). At first glance, it appears counterintuitive that a progenitor population in homeostasis should have higher ROS levels than its differentiated descendants. However, this relatively high ROS is physiologically generated in wild-type flies and is clearly important for hematopoietic development (Owusu-Ansah and Banerjee 2009; Gao *et al.* 2014). This high progenitor ROS production is conserved in mammals where the common myeloid precursor shows three orders of magnitude higher ROS levels than the HSC precursor (Tothova *et al.* 2007). In *Drosophila* hematopoiesis, ROS function as signaling molecules, a phenomenon that has multiple well-established counterparts in other systems [reviewed in Rovira and Finkel (2008) and Schieber and Chandel (2014)]. Removal of the basal level of ROS from the cells of the MZ by expressing antioxidant enzymes adversely affects the formation of mature hemocytes. Similarly, artificial induction of excess ROS in the progenitors upon the attenuation of oxidative phosphorylation causes a huge increase in the number of all three mature hemocyte types, and also elevates activation of the JNK pathway. The hyperdifferentiation effect seen in the raised ROS background is not only suppressed by scavenging free radicals, but is also rescued when the JNK signal is blocked (Owusu-Ansah and Banerjee 2009). Overexpression of the JNK target effector FOXO alone causes premature and excessive differentiation of plasmatocytes and crystal cells, but does not induce lamellocytes (Owusu-Ansah and Banerjee 2009; Gao *et al.* 2014). However, simultaneous activation of FOXO and the removal of polycomb group repressors is able to induce lamellocytes. Increased ROS also result in a reduction in E-cad expression, partly due to JNK activity but also through a JNK-independent, but Srp-dependent, pathway (Gao *et al.* 2014). These data establish that a moderately high but physiologically controlled level of ROS is essential for progenitor differentiation, but further elevation of ROS within the progenitors is sensed as an oxidative stress signal that promotes premature differentiation.

#### FGF pathway:

FGF ligands Thisbe (Ths) and Pyramus (Pyr), as well as the receptor Htl, have been implicated in the maturation of the progenitor cell population within the MZ. Htl protein and *ths* mRNA expression overlap with high E-cad in the progenitors, and only rarely colocalize with Pxn-expressing cells. The ECM protein Trol seems to be important in binding the ligands and limiting the signals to a local area. Inhibition of the FGF pathway throughout the MZ causes an expansion of the progenitor population with fewer mature hemocytes. Overactivation of the FGF pathway within the MZ forces progenitors to excessively differentiate into all three mature blood cell types. Overactivation of the Ras pathway phenocopies overactivation of the FGF pathway, except that lamellocytes do not increase. Downstream of Ras, the transcription factors Pointed (Pnt) and Ush promote progenitor differentiation. Taken together, these data indicate that the FGF signal—through Htl, Ras/MAPK, Pnt, and Ush—promotes the differentiation of progenitors within the lymph gland (Dragojlovic-Munther and Martinez-Agosto 2013). Given the importance of receptor tyrosine kinase (RTK) signaling in all major developmental events, it will be important to further investigate the role of FGF in this system.

#### Collier:

The Collier protein is the first identified marker of the small group of cells that were later termed the PSC (Crozatier *et al.* 2004; Mandal *et al.* 2007). Antibody staining for Collier shows that the protein is expressed at a high level within the PSC but also at a much lower, but physiologically relevant, level within the MZ. Knockdown of *collier* in the progenitors leads to increased differentiation of both plasmatocytes and crystal cells, and a loss of MZ markers such as mRNA for *latran* and *tep4* (Benmimoun *et al.* 2015; Oyallon *et al.* 2016). Reciprocally, genetic elimination of the PSC or alterations of its size does not affect levels of Collier in the MZ, further reinforcing the idea that Collier expression in the MZ functions without contribution from the PSC. The low level of Collier in the MZ is downregulated upon wasp infection, and this reduction allows for a robust production of lamellocytes and premature dispersal of the lymph gland. Overexpression of Collier prevents this lamellocyte formation (Oyallon *et al.* 2016). Collier overexpression in progenitors leads to a larger progenitor population and elevated levels of the heparan sulfate proteoglycan-binding protein Dally-like (Dlp) (Benmimoun *et al.* 2015; Oyallon *et al.* 2016). Whether the low level of Collier expression in the MZ is dependent upon nonautonomous signaling is unclear, but a known autonomous inhibitor is Jumu. This role of Jumu is in addition to its nonautonomous function in the control of dMyc in the PSC, which affects the number and spatial arrangement of cells in this zone (Hao and Jin 2017).

#### Equilibrium signal:

The PSC cells not only send a direct Hh-derived niche signal to the progenitors, they also secrete another ligand that is involved in Pvr signaling. This ligand is interpreted by the CZ cells, which in turn send out a backward signal to the MZ that is termed the equilibrium signal (Mondal *et al.* 2011, 2014) (Figure 5B). Pvf1, one of the three ligands for *Drosophila* Pvr, has high levels of staining within the PSC and is, in addition, seen in punctate spots throughout the lymph gland (Mondal *et al.* 2011). Reduction of Pvf1 within the PSC does not alter levels of Hh expression, nor does it affect the shape or number of cells within the PSC. Instead, decreasing Pvf1 in PSC cells causes a loss of progenitor fate and excessive differentiation (Mondal *et al.* 2011). Pvr is expressed at low levels in cells of the MZ and at very high levels in the CZ. Expression of Pvr in the CZ depends upon the proteins Bip1, Nup98-96, Rps8, and Scalloped. Depletion of Pvr itself, or any of these regulatory proteins in the CZ, gives a phenotype that mimics the loss of progenitors seen when Pvf1 is removed from the PSC (Mondal *et al.* 2011, 2014; Ferguson and Martinez-Agosto 2017). The long-distance transmission of Pvf1 from the PSC to the CZ involves transport vesicles, binding of Pvf1 to Pvr at the surface of MZ cells, and transcytosis of the complex through repeated rounds of vesicular exchanges between cells. The interaction between Pvf1 and the very high Pvr content of the CZ initiates a STAT-dependent but JAK-independent signal within the CZ cells that induces expression of the secreted protein Adenosine deaminase growth factor-A (Adgf-A). This enzyme catalyzes the deamination of adenosine, converting the extracellular signaling molecule adenosine into inert inosine. Although the *Adgf-A* transcript is widely expressed in the lymph gland (Zurovec *et al.* 2002), the function of the enzyme is required in the CZ to transmit the equilibrium signal. The attenuation of adenosine by Adgf-A secreted from the CZ dampens the G protein-coupled adenosine receptor (AdoR) signal in the MZ, resulting in reduced PKA (cAMP-dependent protein kinase A) activity and the consequent maintenance of active Ci. Interestingly, PKA function is also inhibited when Hh binds its receptor, Ptc, activating Ci. Thus, the Hh-dependent signal from the PSC and the adenosine signal controlled by factors within the CZ synergize to inhibit PKA activity and stabilize the activated form of Ci, leading to the maintenance of progenitors within the MZ (Mandal *et al.* 2007; Mondal *et al.* 2011). This two-way communication between the zones reinforces a balance between the progenitor and the mature hemocyte populations.

### The PSC

#### Summary:

The PSC sends out a plethora of signaling ligands and is relatively sparse in its expression of the corresponding receptors, which are largely confined to the MZ. This fact is at the core of the designation of the PSC as a hematopoietic niche. Loss of these ligands leads to disruption of progenitor maintenance in the MZ. In addition, mechanisms of autonomous regulation of PSC cell-fate specification, proliferation, maintenance, and growth have also been explored.

In a pioneering set of experiments, the PSC cells were genetically ablated by artificially inducing the proapoptotic protein Reaper in these cells from the early first instar of larval development. Surprisingly, this manipulation does not alter Collier expression in the MZ and does not appreciably increase hemocyte differentiation, as would be expected upon loss of a Hh-driven signaling center under normal conditions (Benmimoun *et al.* 2015). Others have repeated this manipulation and, at least qualitatively, reported the same results (Oyallon *et al.* 2016; Baldeosingh *et al.* 2018). This finding seems to be at odds with the concept of the PSC cells constituting a hematopoietic niche (Benmimoun *et al.* 2015), and yet multiple laboratories utilizing a variety of genetic backgrounds have demonstrated that when present, the PSC influences the fate of the MZ cells in significant ways (Crozatier *et al.* 2004; Krzemień *et al.* 2007; Mandal *et al.* 2007; Mondal *et al.* 2011; Benmimoun *et al.* 2012; Pennetier *et al.* 2012; Tokusumi *et al.* 2012; Khadilkar *et al.* 2014, 2017; Dey *et al.* 2016; Baldeosingh *et al.* 2018). Several alternative explanations could be put forth to explain these differences. One reasonable starting point in resolving the inconsistency is provided by the Fossett laboratory, which showed that the MZ cells are not all identical as they are composed of a mixture of Hh-sensitive (Odd^+^ Col^−^) and Hh-insensitive (Odd^+^ Col^+^) progenitors (Baldeosingh *et al.* 2018). This is consistent with earlier data that Col^+^ cells constitute a subset of the Dome^+^ progenitors (Oyallon *et al.* 2016), but the critical observation in the current context is the demonstration of their heterogeneity in responding to the niche signal (Baldeosingh *et al.* 2018). For instance, when an Antp driver is used to express *hh-RNAi* in the PSC, the Odd^+^ Col^−^ cells are lost while the Odd^+^ Col^+^ population remains (Baldeosingh *et al.* 2018). Based on these studies, ablation of the PSC would still retain some progenitors as a back-up system, perhaps to be used in the event of stress or infection. Given their location and developmental history, it is tempting to speculate that these Col^+^ cells are direct derivatives of the preprogenitor population that is induced and maintained by signals related to the dorsal vessel (Dey *et al.* 2016; Ferguson and Martinez-Agosto 2017), but further experiments will test the validity of this conjecture.

#### Specifics:

Antp and Collier are used as primary markers for the PSC and, although *Serrate-lacZ* can also be used as a later marker for a subset of these cells, Serrate protein has no known function in the PSC (Lebestky *et al.* 2003; Crozatier *et al.* 2004; Mandal *et al.* 2007). Both Antp and Collier are initially expressed in 3–5 cells of the embryonic lymph gland at stage 16. The PSC cells are the first to proliferate during early larval development and generate the cluster of 30–40 cells seen in the third instar. Antp and Collier expression is maintained in PSC cells throughout all larval instars. Antp is an autonomous determinant of PSC cells, while Collier functions downstream but is required for the maintenance of Antp expression in these cells. Overexpression of Antp causes an expansion in the number of PSC cells, suggesting that Antp specifies both the fate and size of the PSC. This manipulation indirectly increases progenitor maintenance and further reduces progenitor proliferation (Lebestky *et al.* 2000; Crozatier *et al.* 2004; Mandal *et al.* 2007). Dorothy (Dot), a UDP glycosyltransferase, is expressed in all cells of the early lymph gland but retracts to the PSC during later stages (Zhou *et al.* 2001; Kimbrell *et al.* 2002; Jung *et al.* 2005). However, the role of Dot in the PSC is not entirely clear.

The Dpp and Wg signaling pathways regulate the size of the PSC in an opposing fashion (Sinenko *et al.* 2009; Pennetier *et al.* 2012). A GFP reporter for a downstream target of Dpp signaling, *Daughters against dpp* (*Ddad*), and pMad (a downstream transcription factor) are expressed in the PSC cells. Genetic manipulations that inhibit Dpp pathway activity increase the number of these cells and this is attributed to an increased frequency of proliferation in the absence of the signal (Pennetier *et al.* 2012). Dpp pathway activity is modulated by the heparan sulfate proteoglycan-binding protein Dally-like (Dlp or Dly), which is highly expressed within the PSC (Belenkaya *et al.* 2004; Sinenko *et al.* 2011; Pennetier *et al.* 2012). A *dlp* mutant fails to activate pMad in the PSC and leads to an increase in PSC size (Sinenko *et al.* 2011; Pennetier *et al.* 2012). In summary, these data provide evidence that the Dpp pathway plays an inhibitory role in PSC proliferation.

The glycoprotein Slit (Labrosse *et al.* 2005a) is expressed in, and secreted by, the dorsal vessel, while its receptors, Roundabout 1 and 2 (Robo 1–2), are highly expressed in the PSC. Binding of secreted Slit to Robo promotes PSC proliferation and clustering (Morin-Poulard *et al.* 2016). Robo loss increases PSC cell number and disrupts their tight clustering, causing them to scatter to more distal and anterior areas. Additionally, a decrease in plasmatocyte and crystal cell formation is observed, suggesting that the size and morphology of the PSC plays a role in the regulation of hemocyte differentiation. Reduction of Slit in the dorsal vessel, but not the PSC, phenocopies the loss of Robo in the PSC (Morin-Poulard *et al.* 2016). Robo signaling activates Dpp, which initiates a signaling cascade that inhibits dMyc expression, thereby limiting the size of the PSC (Pennetier *et al.* 2012; Morin-Poulard *et al.* 2016). The scattered PSC morphology in *robo* mutants is controlled in part by E-cad, which is lost from the PSC in this mutant background (Morin-Poulard *et al.* 2016).

In contrast to the inhibitory role of Dpp in the determination of PSC size, Wg signaling positively regulates the number of PSC cells (Sinenko *et al.* 2009; Pennetier *et al.* 2012). The Wg signaling components, Fz2, β-catenin/Arm, and Disheveled (Dsh) are all expressed within the PSC. Loss of Wg signaling within this zone causes a reduction in cell number, while overexpression of Wg increases the PSC population, phenocopying the inhibition of Dpp signaling. Simultaneously blocking both pathways rescues the PSC to wild-type size. The regulation of PSC cell number by Wg is dependent on dMyc, since simultaneous overexpression of Wg and knock down of dMyc leads to normal PSC cell number (Pennetier *et al.* 2012). Jumu, a forkhead family transcription factor, is expressed throughout the entire lymph gland but is enriched within the PSC. Gain- and loss-of-function experiments suggest that Jumu regulates dMyc to control PSC cell proliferation throughout development (Hao and Jin 2017).

Septate junctions (similar to vertebrate tight junctions) between PSC cells function upstream to regulate Dpp and Wg signaling pathways, and affect hematopoiesis under normal and stress conditions (Khadilkar *et al.* 2017). The septate junctions within the PSC are found to be disrupted in response to bacterial infection, or when Toll or Imd pathways are activated in the PSC during the larval stage. During normal development, the PSC is impermeable to large molecular dyes, but this barrier is disrupted upon loss of Coracle (Cora) or Neurexin IV (NrxIV) specifically within the PSC. Increased permeability in these mutant backgrounds is associated with an increased number of PSC cells, mature plasmatocytes, and crystal cells. Disruption of the permeability barrier in this way causes a smaller MZ, but the overall size of the lymph gland is, in fact, larger. Loss of septate junctions alters both Wg and Dpp signaling pathways, perhaps by affecting ligand distribution and reception (Khadilkar *et al.* 2017).

The insulin pathway also regulates the proliferation and growth of PSC cells (Benmimoun *et al.* 2012; Dragojlovic-Munther and Martinez-Agosto 2012; Tokusumi *et al.* 2012). Insulin-like peptides are endogenously expressed in neurons, glia, and the fat body, but not the lymph gland [reviewed in Nässel *et al.* (2015)]. Therefore, this is an example of a systemic signal controlling the PSC. Overactivation of insulin signaling within the PSC cells increases their proliferation. Inhibition of insulin signaling by manipulation of the insulin receptor (InR), overexpression of the pathway inhibitor PTEN, or downregulation of positive components of the pathway, Akt1, Pdk1, or phosphoinositide-3 kinase (PI3K), reduces the number of PSC cells. Downstream of InR, the Target of Rapamycin (TOR) pathway functions to regulate growth of the Antp^+^ cells. TORC1 and TORC2 are both involved in this process, as is Tsc1/2, S6K, and Thor (Benmimoun *et al.* 2012; Dragojlovic-Munther and Martinez-Agosto 2012; Tokusumi *et al.* 2012).

### The Posterior lobes

The secondary, tertiary, and quaternary lobes (collectively, the posterior lobes) become prominent during the second and third larval instars (Figure 3B). The cells within these lobes remain undifferentiated except under stress conditions. Morphologically, the posterior lobes look similar to the MZ of the primary lobe, and contain tightly packed progenitors with high E-cad, Dome, and Collier expression (Jung *et al.* 2005; Benmimoun *et al.* 2015). These lobes lack expression of maturing hemocyte markers such as Collagen IV, Hml, Pxn, and P1 in a manner similar to that seen in the primary lobes prior to the initiation of differentiation (Jung *et al.* 2005). During the prepupal stages, the posterior lobes begin to express mature hemocyte markers, with a corresponding downregulation of Dome and E-cad indicative of a switch from progenitor to differentiated state (Grigorian *et al.* 2011b). A fraction of these posterior lobe cells contribute to the hematopoietic hubs of the adult fly (Ghosh *et al.* 2015).

In certain genetic backgrounds in which the primary lobes overdifferentiate and/or lose progenitors, the posterior lobes also react with increased differentiation rates, increased numbers of lobes, or increased size compared with wild-type flies (Sorrentino *et al.* 2002; Stofanko *et al.* 2008; Owusu-Ansah and Banerjee 2009; Kulkarni *et al.* 2011; Mondal *et al.* 2011; Khadilkar *et al.* 2017). Several of these genes that increase the size of the posterior lobes were identified in an overexpression screen utilizing *Pxn-GAL4* (Stofanko *et al.* 2008). Enlarged secondary lobes, usually associated with overdifferentiation, are seen upon elevation of ROS, overexpression of the FGF ligands Thisbe and Pyramus, loss of Jumu, or loss of Arj (Owusu-Ansah and Banerjee 2009; Kulkarni *et al.* 2011; Dragojlovic-Munther and Martinez-Agosto 2013). In addition to these phenotypes, extranumerary posterior lobes are seen in *asrij* mutant animals and ectopic PSC cells are present in the secondary lobes of *jumu* mutants (Kulkarni *et al.* 2011; Khadilkar *et al.* 2017). Premature differentiation is the most prominent defect observed in the posterior lobes in animals mutant for *Tiggrin* or upon wasp parasitization (Sorrentino *et al.* 2002; Zhang and Cadigan 2017). These phenotypes indicate that the molecular mechanisms that regulate hemocyte differentiation within the primary and posterior lobes might be similar, although they are employed at distinct stages of development.

## Pupal and Adult Blood Cells

### Summary

At the onset of pupariation, the lymph gland undergoes a dramatic change. The progenitor population differentiates leaving very few, if any, cells that are Dome^+^ (Grigorian *et al.* 2011b). The lymph gland disintegrates, dispersing the blood cells into the hemolymph (Lanot *et al.* 2001; Grigorian *et al.* 2011b). The blood cells released in circulation engulf the large number of apoptotic cells generated during tissue remodeling associated with pupal development and remove bacteria released during the remodeling of the gut. This process of dispersion also allows a mixing of the blood cells from the two major hematopoietic waves that together populate the circulating and sessile pools of blood cells in the adult fly (Lanot *et al.* 2001; Grigorian *et al.* 2011b). While most of the dispersed lymph gland cells, together with those in larval circulation, go on to become adult circulating blood cells (Holz *et al.* 2003), some of them populate hubs along the body wall of the adult abdomen (Ghosh *et al.* 2015). These hubs are proposed to serve as active sites of adult hematopoiesis, an important finding that is worthy of further investigation.

Pulses of the fly steroid hormone 20-hydroxyecdysterone (commonly referred to as ecdysone for simplicity) that initiate pupariation [reviewed in Thummel (2001)] also trigger the final burst of differentiation and disintegration of the lymph gland. In fact, lymph glands are severely hypertrophic and fail to disperse in temperature-sensitive *ecdysoneless* mutants (Sorrentino *et al.* 2002). Hemocytes in circulation also display ecdysone-related alterations to their morphology and function. When ecdysone is injected into midthird instar larvae, plasmatocytes become more flattened, adherent, and more phagocytic, similar to those seen in the white prepupal stage (Lanot *et al.* 2001; Regan *et al.* 2013). Expression of a dominant negative version of the ecdysone receptor (*EcRB1^DN^*) in blood cells also prevents their dispersal from sessile clusters on the body wall (Regan *et al.* 2013). Thus, ecdysone signaling plays diverse roles in directing hematopoiesis during pupal development that together cause the dissociation, dispersal, and activation of blood cells, fulfilling the demands of metamorphosis and eclosion.

### Specifics

Under normal culture conditions, the lymph gland does not contribute to the blood cells in circulation and remains intact throughout all larval stages, with cells encased in ECM components (Lanot *et al.* 2001; Grigorian *et al.* 2011b). During the pupal stage, lymph gland cells break away from these matrix components as they exit the lymph gland and enter circulation (Grigorian *et al.* 2011b). Upon their release, the plasmatocytes phagocytose larval muscle cells, the ECM surrounding lymph gland cells, and even other plasmatocytes (Lanot *et al.* 2001). Markers such as Pxn and P1 are detected throughout the lymph gland at 4 and 8 hr after puparium formation, respectively, indicating that progenitors differentiate prior to dispersal. Trol protein is usually more concentrated around progenitors of the MZ than the differentiated cells in the CZ. However, during pupation, this distinction in density is lost as Trol becomes uniformly distributed throughout the lymph gland. The Antp^+^ PSC cells are also affected, as they no longer remain confined in a small cluster in the posterior region of the lymph gland, and begin to spread throughout the primary lobes by 8–12 hr after puparium formation (Grigorian *et al.* 2011b). The significance of this observation is as yet unclear.

As the primary lobes differentiate and disperse during pupation, the secondary and tertiary lobes proliferate, enlarge, initiate differentiation, and express Pxn, but not the late marker P1 (Grigorian *et al.* 2011b). The cells within these lobes then disperse and enter into circulation beginning at 10 hr after puparium formation (Grigorian *et al.* 2011b). Lineage trace analysis suggests that progenitors from the tertiary and quaternary lobes of the lymph gland do not fully differentiate, and instead migrate to hubs on the body wall in the adult, which may serve as an adult hematopoietic niche (Ghosh *et al.* 2015).

Early studies established that adult hemocytes do not proliferate during homeostasis (Lanot *et al.* 2001). However, upon immune challenge, several clusters of hemocytes located in hubs along the dorsal abdomen incorporate BrdU (Ghosh *et al.* 2015). Despite lacking proliferative capacity during normal development, these hubs increase in size for up to 5 days posteclosion, presumably due to localized homing of circulating cells to these sites. All adult hemocytes in the hubs express Srp and are dependent on ECM components in the neighboring microenvironment for their localization. Lineage tracing shows that the plasmatocytes and crystal cells in these hubs are derived from hemocytes of embryonic and larval origin. Interestingly, this study also detects hemocyte progenitors among the hub cells that are Srp^+^ but lack Hml or Hnt, suggesting that the hubs could serve as a site of hematopoiesis (Ghosh *et al.* 2015). This is a foundational study on adult hematopoiesis in *Drosophila* and, given the interest in, and the importance of, adult stem and progenitor populations across species, these intriguing results warrant further investigation.

## Nutritional and Sensory Control of Hematopoiesis

**Figure 6:**
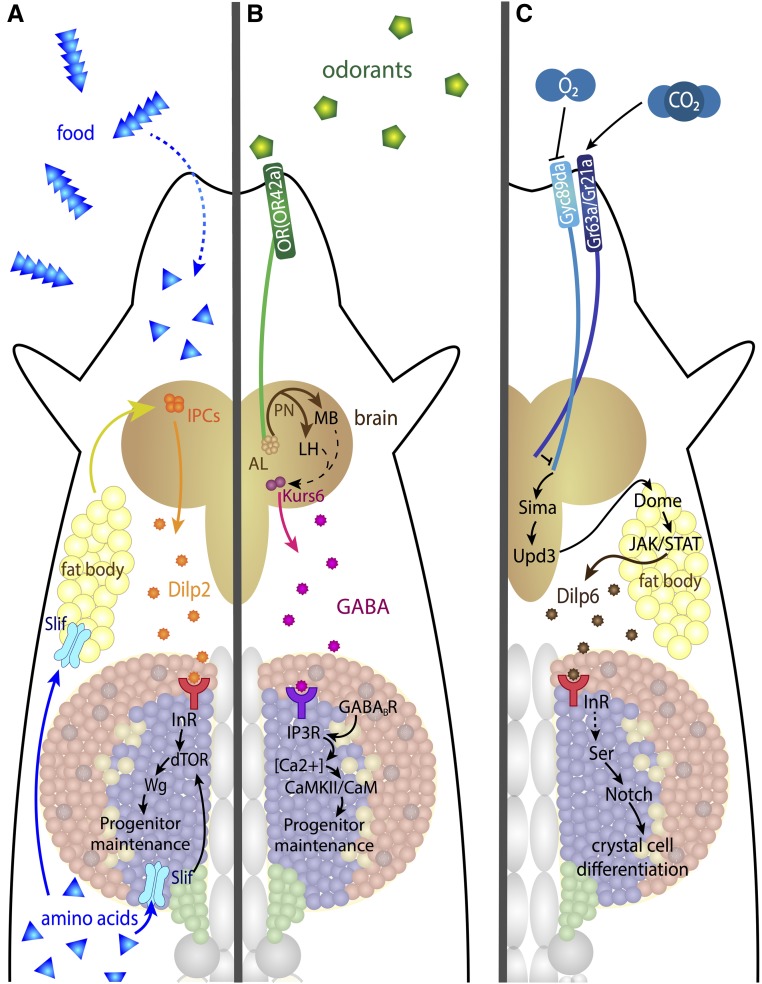
Control of larval hematopoiesis by nutrition, olfaction, and hypoxia. (A) Ingested proteins are broken down into individual amino acids and absorbed into the hemolymph. The cells of the fat body and the medullary zone (MZ) express the amino acid transporter Slimfast (Slif), which allows amino acids to enter these organs. A systemic signal from the fat body then activates Dilp2-expressing insulin-producing cells (IPCs) within the brain. Secreted Dilp2 binds the Insulin receptor (InR) expressed in the MZ. Target of Rapamycin (TOR) activation downstream of InR is aided by amino acid-related signals. Wingless (Wg) functions downstream of TOR to promote progenitor maintenance. (B) Environmental odors are sensed by the OR42a^+^ neuron of the terminal organ, which subsequently activates a neuronal circuit that involves the antennal lobes (AL), projection neurons (PN), mushroom bodies (MB), and lateral horns (LH). This process activates Kurs6^+^ neurosecretory cells that secrete γ-aminobutyric acid (GABA) into the hemolymph, leading to GABA_B_R activation in the MZ progenitors. The downstream calcium signal enables progenitor maintenance. (C) The heterodimeric receptor complex made up of Gr63a and Gr21a is activated upon CO_2_ binding. Normal environmental CO_2_ levels keep the CO_2_-sensing neuron active. Oxygen inhibits the atypical guanylyl cyclase, Gyc89da, and the Gyc89da^+^ neurons become active under hypoxic conditions. The hypoxia- and CO_2_-sensing neurons both project from the terminal organ to the subesophageal ganglion of the brain, where they form inhibitory synapses such that an active CO_2_-sensing neuron further inhibits the hypoxia-sensing neurons under normoxia. Under hypoxic conditions, activation of the hypoxia-sensing neuron leads to stabilization of Sima in a specific set of ventral nerve cord neurons, a process that ultimately results in a brain-initiated cytokine signal, Upd3, which activates Domeless (Dome) in the fat body. Downstream of this signal, Dilp6 is generated by the fat body cells, which then binds InR in lymph gland blood precursors to increase Serrate levels and therefore Notch signaling. The net result of this multiorgan signaling cascade is that, under hypoxic conditions or loss of CO_2_ reception, the excess Notch activation causes an increase in crystal cell number.

*Drosophila* blood cells respond to a lack of nutritional content (Benmimoun *et al.* 2012; Dragojlovic-Munther and Martinez-Agosto 2012; Shim *et al.* 2012), lack of favorable odors (Shim *et al.* 2013), or the depletion of environmental CO_2_ or O_2_ (Cho *et al.* 2018) (Figure 6). The response to a lack of nutrition involves insulin production from the brain and its reception by lymph gland progenitors. The other two cases involve the detection of small odorants or gaseous ligands by external sensory neurons in the terminal organ. These signals are both transduced through a multiorgan hormone-dependent signaling cascade to the cells of the lymph gland. However, when these two pathways are disrupted by loss of olfaction or hypoxia, they give rise to distinct hematopoietic defects.

### Nutritional control

#### Summary:

Nutritional status is prominent among systemic signals that regulate lymph gland development (Figure 6A). Insulin and its receptor (InR), PI3K, Akt, and TOR participate in controlling progenitor maintenance in response to amino acid levels within the hemolymph (Dragojlovic-Munther and Martinez-Agosto 2012; Kroeger *et al.* 2012; Shim *et al.* 2012). Systemic cytokine signals trigger secretion of the *Drosophila* insulin like peptide 2 (Dilp2) from the insulin-producing neurosecretory cells of the brain (Shim *et al.* 2012). Expression of InR is limited to the cells of the MZ, and they therefore normally receive a steady level of this RTK signal as long as the larvae are well fed. In parallel, the cells of the MZ also directly detect amino acids through the function of a transporter, Slimfast (Slif), expressed in them. The transported amino acids help activate the TOR protein as an additional nutritional signal [reviewed in Nässel *et al.* (2015)]. The combined InR/PI3K/Akt/TOR activation feeds into the Wnt pathway in the MZ to promote progenitor maintenance (Shim *et al.* 2012). Starvation reduces amino acid levels and relieves progenitor maintenance, increasing differentiation and the potential for innate immune response. Furthermore, the starvation phenotype seen in the lymph gland can be rescued when Wg is overexpressed in progenitors (Shim *et al.* 2012). Insulin-related signaling involves multiple tissue types. For example, muscle cells respond to specific Dilps to influence lamellocyte encapsulation in response to infection during starvation (Yang and Hultmark 2017). The larvae respond to a lack of nutrients by activating the Toll pathway within hemocytes in circulation and in the lymph gland, in a manner reminiscent of the stress response induced when a larva is challenged with wasp parasitization (Shim *et al.* 2012; Louradour *et al.* 2017). A high-sugar diet in larvae induces lamellocytes via JNK, and also decreases phagocytosis of large particles such as fungal spores and latex beads (Yu *et al.* 2018). In adult flies, a high-fat diet causes global activation of the JAK/STAT pathway and the adult circulating cells secrete Upd3, which triggers a systemic immune response (Woodcock *et al.* 2015).

#### Specifics:

Larvae raised under starvation conditions have excessive differentiation at the expense of lymph gland progenitors. There is an obvious increase in the number of circulating plasmatocytes and lamellocytes. Plasmatocytes are also often seen interspersed between fat body cells in a starved larva (Shim *et al.* 2012). In response to starvation, crystal cells prematurely differentiate in the second instar and subsequently rupture during the third instar (Benmimoun *et al.* 2012; Shim *et al.* 2012). Furthermore, larvae respond to starvation by activating the Toll pathway, which leads to accumulation of the transcription factor Dorsal in the nucleus in circulating hemocytes and in lymph gland cells (Shim *et al.* 2012).

There are eight insulin-like peptides in *Drosophila* (Dilp1–8) that act as ligands for the InR [reviewed in Nässel *et al.* (2015)]. Dilp2, in particular, is involved in regulating hematopoiesis during normal development and upon starvation, and a decrease in Dilp2 is the major cause of blood cell overdifferentiation. Consistent with this explanation, elimination of insulin-producing cells (IPCs) in the brain causes a loss of progenitors similar to that seen upon starvation, and constitutive activation of the IPC neurons induces increased progenitor maintenance. Similarly, loss of InR or its downstream effector Chico, or Akt from the hemocyte progenitors, increases differentiation of the progenitor population within the lymph gland (Shim *et al.* 2012). TOR kinase functions further downstream in the InR pathway, but it also responds to amino acid availability (Oldham *et al.* 2000a,b; Zhang *et al.* 2000; Kim *et al.* 2008; Teleman *et al.* 2008; Oldham 2011). TOR integrates these two inputs through a pathway that includes the translation initiation factor 4E-binding protein (4EBP, also known as Thor) and ribosomal protein S6 kinase (S6K) (Rodriguez *et al.* 1996; Montagne *et al.* 1999). Upstream effectors of TOR, Rheb, and Tsc2, as well as the downstream component p-4EBP, are expressed in high amounts in the progenitors and this is supportive of active TOR signaling. Gain- and loss-of-function experiments involving TSC1/2, Rheb, and PTEN suggest a relationship between the entire TOR pathway and ROS, and this regulation is likely linked to the starvation-induced lymph gland phenotype (Dragojlovic-Munther and Martinez-Agosto 2012; Shim *et al.* 2012).

The fat body also detects amino acid levels within the hemolymph via an amino acid transporter called Slif, and this results in a paracrine signal to the brain that activates Dilp2 secretion. In addition to the Dilp2/InR response to nutrition, blood progenitors also respond directly to essential amino acids imported into the cell by Slif (Shim *et al.* 2012). Thus, amino acid levels are detected both directly by the lymph gland progenitors and also indirectly via the fat body to maintain the pool of hemocyte progenitors (Benmimoun *et al.* 2012; Shim *et al.* 2012). Similar to the lack of nutrition, a lack of amino acid detection leads to a loss of the progenitor population and an increase in the number of mature blood cells.

Increasing the amount of fat in the diet of adult flies leads to changes in hemocyte gene expression (Woodcock *et al.* 2015). Under these conditions, adult hemocytes in circulation have higher expression of Upd3. This result suggests that plasmatocytes are able to sense lipid levels in the hemolymph. Genetic dissection shows that this nutrient sensing phenomenon requires the Crq receptor, which presumably allows for the uptake of lipids from the hemolymph into the plasmatocytes. Reduction of *crq* within hemocytes causes decreases in both Upd3 secretion and Socs36e transcription within hemocytes. Excess lipid consumption does not activate the Toll or Imd pathways, and also does not alter the number of plasmatocytes in circulation. Rather, the affected plasmatocytes increase their lipid stores and display an altered morphology. The resulting increase in Upd3 expression in hemocytes is either directly or indirectly involved in decreasing the adult life span when flies are fed a high-fat diet (Woodcock *et al.* 2015).

### Sensory control

Environmental odors and gases sensed by “gustatory” receptors also trigger a cascade of signaling events involving external sensory organs, the brain, and the developing lymph gland to regulate the blood progenitor population. GABA_B_R, a metabotropic receptor for GABA, is expressed in both the PSC and MZ. Within the PSC, GABA_B_R plays an early role, before midsecond instar, in promoting lymph gland growth. Importantly, the MZ-expressed GABA_B_R is responsible for progenitor maintenance in response to sensory cues. Environmental odiferous ligands are sensed through the larval terminal organ and detected by a neuron expressing the receptor OR42a that senses fruity odors. This neuron projects into the antennal lobe of the brain and interacts via a network of interneurons that ultimately activate a small number of neurosecretory cells to secrete GABA into the hemolymph. The binding of GABA to GABA_B_R expressed in the MZ is essential for progenitor maintenance that is dependent on downstream Ca^2+^ signaling. Loss of olfaction is sensed as a stress and the progenitors respond by losing their quiescence and multipotency (Shim *et al.* 2013) (Figure 6B). Environmental gases are also detected and interpreted in a similar manner utilizing external receptors in the terminal organ. This leads to secretion of a cytokine across the blood–brain barrier and, with the fat body as an intermediary organ, production of an insulin-like peptide that ultimately influences crystal cell development within the lymph gland through the regulation of Notch signaling (Figure 6C).

#### Olfactory control:

Olfaction is relayed by multiple organs using signaling peptides to exert an effect on hematopoiesis. Lymph glands of larvae raised on food lacking odorants have a decreased pool of blood progenitors and increased number of differentiated cells, despite the PSC being unaffected. Amazingly, the mutant lymph gland phenotype caused when larvae are grown on food lacking odorants can be rescued when a small dialysis bag containing odorant food is placed within the odor-free growth medium. In this experiment, larvae are able to detect the food smell, but are unable to ingest the odorant food due to the barrier presented by the dialysis membrane (Shim *et al.* 2013).

Odorants are detected by external sensory neurons, which then transmit a neuronal signal to the subesophageal ganglion of the brain. This information is further relayed to downstream projection neurons that interact with *Kurs6-GAL4^+^* neurosecretory cells to activate GABA secretion into the hemolymph. A high level of GABA is detected within *Kurs6^+^* neurons and also at the surface of lymph gland progenitors. A loss of GABA synthesis or inhibition of its proper vesicular packaging within the *Kurs6^+^* neurons causes a reduction of GABA in circulation, decreased detection of GABA on the MZ cells, and increased hemocyte differentiation with a loss of progenitors. Genetic ablation, inhibition of neuronal activity, or depletion of olfactory coreceptor Orco within all olfactory neurons decreases GABA staining in the *Kurs6^+^* neurons, as well as in circulation and on the MZ cells. This ultimately results in a loss of lymph gland progenitors, mimicking the loss-of-odorant phenotype. These data suggest that *Kurs6^+^* GABA-producing cells function downstream of olfactory perception to translate the sensory signal to one that controls blood cell development (Shim *et al.* 2013).

Reception of GABA secreted from the *Kurs6^+^* neurons by progenitors in the MZ activates cytosolic calcium signaling, which promotes progenitor maintenance. Calcium signaling activity, indicated by a GCaMP sensor, decreases in progenitors expressing RNAi against either subunit of the GABA receptor (GABA_B_R1 or GABA_B_R2). This suggests that the ultimate step in olfactory regulation downstream of Or42a in larval hematopoiesis is the modulation of calcium within the progenitors to control levels of hemocyte differentiation. Olfactory perception is independent of insulin signaling and does not affect food intake. Unlike starvation, olfactory deprivation does not affect levels of Dilp2 secretion. Furthermore, Dilp2 expression within the IPCs is unaltered when GABA production is decreased in the *Kurs6^+^* neurons (Shim *et al.* 2013).

#### Control by environmental gases:

Although classically named as gustatory receptors, the heterodimeric partners Gr63a and Gr21a have been shown to be activated by low levels of CO_2_ in adults and in larvae (Jones *et al.* 2007; Kwon *et al.* 2007). Hypoxia and low levels of ROS molecules have been known to activate an atypical guanylyl cyclase, Gyc89da (Morton 2004; Evgenov *et al.* 2006; Morton *et al.* 2008; Vermehren-Schmaedick *et al.* 2010). Disruption of any of these three molecules (Gr63a, Gr21a, or Gyc89da) in their respective neurons leads to an increase in lymph gland production of crystal cells (Cho *et al.* 2018).

The CO_2_- and hypoxia-sensing neurons interact in the brain to convert the external sensation of gases into a signal that can cross the blood–brain barrier to have a systemic effect on the animal. Dendritic labeling reveals that the CO_2_-sensing neuron and the hypoxia-sensing neurons are located near each other in the terminal organ of the larval head, and then overlap within the subesophageal ganglion of the brain. It is at this juxtaposition that these two neurons utilize GABA signaling to form synaptic connections with each other, which can be visualized using the molecular proximity detector GRASP (Feinberg *et al.* 2008; Macpherson *et al.* 2015). Larvae grown in a normoxic environment have regular Sima accumulation in particular neurons of the ventral nerve cord (VNC). However, when CO_2_ or oxygen levels are decreased during development, the Sima protein is stabilized in Gyc89da^+^ cells in the VNC. This elevated Sima leads to increased production of the Upd3 cytokine in hypoxia-sensing neurons. The inhibition of neuronal expression of either Sima or Upd3 can suppress the increased crystal cell phenotype seen under low CO_2_ conditions (Cho *et al.* 2018).

After secretion into the hemolymph, the Upd3 released by the brain is received by the Dome receptor expressed in the fat body. This binding triggers the JAK/STAT signaling pathway in this organ, which can be detected by higher levels of the STAT transcriptional target Socs36E. Also dependent upon increased JAK/STAT activity in the fat body is the augmented level of Dilp6. Upon inhibition of CO_2_ reception, this Dilp6 is required for the downstream lymph gland production of crystal cells. Finally, the high amounts of Dilp6 are received by the InR expressed in the lymph gland to increase the production of crystal cells. Decreasing the amount of CO_2_ detection leads to high levels of pAKT and p4EBP/Thor throughout the lymph gland. Upregulation of Serrate in the second-instar mutant lymph gland promotes increased crystal cell differentiation (Cho *et al.* 2018). It is through this multisignal and multiorgan relay system that both external gaseous and odiferous ligands are able to affect hematopoietic development to respond to the environment.

## Stress Response and the Hematopoietic System

It can be argued that all cells are programmed to respond to stresses such as changes in redox levels, unfolded proteins, DNA damage, infection, injury, or metabolic dysfunction. Internal circuitry relieves such stresses by activating mechanisms ranging from antioxidant pathways, the unfolded protein response, autophagy, cell cycle arrest, repair, and a plethora of cell death pathways. However, myeloid precursors and the cells derived from them are specialists at mitigating many forms of stresses that disrupt homeostasis. This becomes readily apparent in *Drosophila* (and invertebrates in general) as their entire blood cell repertoire is similar to the myeloid cells in vertebrates [reviewed in Evans *et al.* (2003)]. Even within the context of innate immunity, the strongest responses are systemic, arising outside the hematopoietic tissue [reviewed in Lemaitre and Hoffmann (2007)]. Also, oxygen distribution is not an essential cellular function in *Drosophila*, although gaseous sensing is certainly important (Mondal *et al.* 2011; Cho *et al.* 2018). The myeloid-like blood cells are sentinels of stress sensation and mitigation, often responding faster than system-wide immunity afforded by the fat body (Kocks *et al.* 2005; Bidla *et al.* 2007; Babcock *et al.* 2008; Cuttell *et al.* 2008). In this context, we broadly define stress as any event or condition that threatens to disrupt homeostasis. These stresses can be intrinsic, arising from abnormal internal processes such as genetic mutations, aberrant tissue characteristics, and growth, or they might initiate from abnormal external processes such as infection, parasitization, a lack of essential nutrients, or injury caused by mechanical, chemical, or heat damage. Interestingly, although these events have very different inducers and follow distinct transduction processes, the blood cell response is often quite similar. Thus, multiple independent stress signals might feed into a few key blood cell response pathways. This is particularly true for the hematopoietic system since stress responses frequently coopt the same pathways that direct homeostatic development, except in an exaggerated form suitable for the specific kind of stress.

### Cellular response to intrinsic stress

#### Summary:

Internal stresses often result from genetic mutations that induce tissue abnormalities. For example, ECM components keep healthy tissues isolated from the hemolymph and their loss can cause blood cell responses that have been likened to an “auto-immune response.” These responses include: the attachment of plasmatocytes onto the affected surface and their invasion into the tissue, activation of plasmatocyte signaling, and encapsulation of the tissue by lamellocytes to form melanotic masses (Pastor-Pareja *et al.* 2008; Cordero *et al.* 2010; Kim and Choe 2014; Fogarty *et al.* 2016). Depletion of basement membrane components, particularly in combination with loss of appropriate cell polarity markers and decreased cell-to-cell contacts, leads to the formation of melanotic masses around self-tissue (Kim and Choe 2014). Similarly, in a metastatic tumor model in the eye/antennal disc, loss of basement membrane components causes the enhanced recruitment and proliferation of plasmatocytes that help limit tumor growth (Pastor-Pareja *et al.* 2008).

Similar phenotypes are seen when mutations directly affect blood cell differentiation, proliferation, or signaling. In this case, hyperactive blood cells respond inappropriately to normal tissues. For example, this occurs in fly models of leukemia, in which oncogenes are introduced into the blood tissue and this results in an increase in circulating blood cells, lamellocyte differentiation, and the formation of melanotic masses around normal tissue (Osman *et al.* 2009; Sinenko *et al.* 2010; Reitman *et al.* 2015). Signs of immune activation including lamellocyte differentiation, lymph gland hypertrophy, increased proliferation, and encapsulation of healthy tissues are also seen when blood differentiation pathways such as Toll, JAK/STAT, and adenosine signaling are overactivated through mutations in pathway components (Gerttula *et al.* 1988; Hanratty and Dearolf 1993; Harrison *et al.* 1995; Lemaitre *et al.* 1995; Luo *et al.* 1995; Qiu *et al.* 1998; Dolezal *et al.* 2005). Interestingly, the fat body and hemocytes respond differently to knockdown of Toll pathway components (Schmid *et al.* 2014). Ultimately, screening for factors that cause these types of overactive blood cell phenotypes has provided insights into pathways involved in their development and stress responses (Watson *et al.* 1991; Zettervall *et al.* 2004; Minakhina and Steward 2006; Avet-Rochex *et al.* 2010).

#### Specifics:

Blood cells respond to abnormal or damaged tissues in several ways. For example, > 100 genetic mutations have been identified that cause encapsulation of self-tissue by lamellocytes and subsequent melanization, forming melanotic masses in the bodies of the fly sometimes called melanotic capsules, melanotic nodules, melanotic tumors, or melanotic pseudotumors (Watson *et al.* 1991; Minakhina and Steward 2006; Avet-Rochex *et al.* 2010; Gramates *et al.* 2017). Melanotic masses often resemble one another, but they have been categorized into two broad classes based on differences in the underlying mechanisms of their formation. Class I melanotic mass mutants have apparently normal cellular immune systems that respond to an abnormal self-tissue. For example, mutations in *kurtz*, encoding an arrestin protein that is involved in the internalization of G protein-coupled receptors, causes fat body cells to dissociate and melanotic masses to form around these dissociating fat body cells (Roman *et al.* 2000). Blood cells identify normal tissue through the detection of an intact basement membrane (Rizki and Rizki 1974). Knockdown of Collagen IV subunits Vkg and α1, or Laminin subunits in fat body cells, disrupts their basement membrane and leads to encapsulation by lamellocytes and the formation of melanotic masses (Kim and Choe 2014). Similarly, knockdown of a receptor for the basement membrane, the integrin-β subunit Myospheroid, also causes a similar phenotype. Reliable encapsulation occurs when basement membrane disruption is combined with a loss of cell integrity, cell-to-cell contacts, and improper localization of polarity proteins such as Cora and Discs large (Kim and Choe 2014).

Class II melanotic masses have been defined as those arising in the context of an overactive immune system that causes ectopic activation of hemocytes and an inappropriate response to normal tissue. Often, class II mutants also exhibit a large increase in the number of blood cells and aberrant lamellocyte differentiation. Pathways such as Toll, JAK/STAT, and adenosine signaling, known to be involved in blood cell homeostasis, give rise to class II phenotypes if they are made overactive (Gerttula *et al.* 1988; Hanratty and Dearolf 1993; Harrison *et al.* 1995; Lemaitre *et al.* 1995; Luo *et al.* 1995; Qiu *et al.* 1998; Dolezal *et al.* 2005; Matova and Anderson 2006; Krzemień *et al.* 2007; Minakhina *et al.* 2011; Zhang *et al.* 2016). A melanotic tumor phenotype similar to class II is induced when human oncogenic aberrations, such as translocations giving rise to the fusion protein *AML1-ETO* or gain-of-function mutations in isocitrate dehydrogenase (IDH1 and 2), are expressed in *Drosophila* hemocytes to simulate models for blood-related disorders (Osman *et al.* 2009; Sinenko *et al.* 2010; Reitman *et al.* 2015). Melanotic tumors are terminal phenotypes that are easy to identify in a genetic screen. Their origins are often linked to defects in proliferation, differentiation, or autoimmunity. Genetic screens are valuable for identifying new factors that contribute to normal and defective hematopoiesis, and also to the cellular stress response.

*Drosophila* has emerged as a powerful system in which to study the complex role that macrophages play in disease progression, such as in a metastatic cancer model induced by the expression of overactive Ras along with a loss-of-function mutation in the *scribbled* (*scrib*) gene that is important for the maintenance of epithelial polarity. These genetic backgrounds cause massive tumors that can become metastatic (Pagliarini and Xu 2003; Pastor-Pareja *et al.* 2008). Importantly for this discussion, mature plasmatocytes migrate to the tumor sites and adhere to them, preferentially binding to areas where the basement membrane is disrupted. Surprisingly, melanotic masses are not observed around *Ras^V12^*; *scrib^−/−^* tumors or in a *Ras^V12^*-driven salivary gland tumor model. In the latter case, plasmatocytes, crystal cells, and lamellocytes migrate to the tumorous tissue, yet no melanotic masses are seen (Hauling *et al.* 2014). Tissue damage induced by a tumor, an aseptic wound, or UV irradiation causes an increase in proliferation and the number of blood cells in circulation that is dependent on JAK/STAT signaling in the blood cells (Pastor-Pareja *et al.* 2008; Karpac *et al.* 2011; Kelsey *et al.* 2012). While modulation of JAK/STAT in hemocytes represses this excessive proliferation, it does not have any effect on the attachment of the existing hemocytes to the tumor surface (Pastor-Pareja *et al.* 2008).

Overall, it appears that stress-induced plasmatocyte migration, proliferation, and signaling aids the restriction of tumor growth and tissue maintenance (Pastor-Pareja *et al.* 2008; Kelsey *et al.* 2012). This is evidenced by the fact that the size of *scrib^−/−^* tumors in the imaginal epithelium increases if blood cells are unable to receive a JAK/STAT signal (Pastor-Pareja *et al.* 2008). Similarly, blood cells also restrict tumor growth induced in the wing disc in a *discs large* mutant background, and in these mutant animals, plasmatocytes express Eiger (Egr), the *Drosophila* homolog of tumor necrosis factor (TNF). Egr expression activates JNK signaling in tumor cells, which induces apoptosis, thus decreasing tumor size (Parisi *et al.* 2014).

Although plasmatocytes normally function to mitigate infection, injury, and apoptotic decay, while fighting artificially induced disease, their attempt to minimize the damage could, in fact, lead to enhanced mortality or dysfunction at the cellular and systems level. For example, in the aforementioned tumor model, if, in addition to the loss of polarity genes, the tumor also expresses *Ras^V12^*, this second mutation leads to a block in apoptosis (Bergmann *et al.* 1998; Pérez *et al.* 2017). In this case, the expression of Egr by plasmatocytes causes very high levels of JNK signaling in the tumor without causing apoptosis, which results in increased metastatic growth (Cordero *et al.* 2010; Pérez *et al.* 2017). In addition, high levels of ROS in the *Ras^V12^*; *scrib^−/−^* tumors recruit plasmatocytes, which consequently amplify JNK signaling and metastatic growth (Pérez *et al.* 2017). Reduction of Egr or the inhibition of hemocyte recruitment through expression of ROS-scavenging enzymes attenuates such metastatic events (Cordero *et al.* 2010; Pérez *et al.* 2017). In a different but analogous scenario, “undead” cells created by simultaneously activating the proapoptotic protein Hid and the antiapoptotic caspase inhibitor p35 cause a characteristic overgrowth known as apoptosis-induced proliferation. Hemocytes expressing Egr, detected in the vicinity of the undead tissue, in fact aid in apoptosis-induced proliferation by activating JNK signaling, and this can be reduced in the epidermal tissues if hemocyte differentiation is prevented using a *serpent* mutant background (Fogarty *et al.* 2016). In a fly model of Alzheimer’s, neurodegeneration is observed when human Aβ42 is overexpressed in the brain. This defect is enhanced following bacterial infection due to the activation of JNK signaling in the brain, in part by Egr-expressing plasmatocytes that bind to the brain (Wu *et al.* 2017). The above results demonstrate the constantly evolving conflict between a growing tumor and the cellular immune response. To defend against the growing tumor, macrophages proliferate and induce apoptosis, but if an additional mutation arises within the tumor to block apoptosis, the same mechanism that the plasmatocyte successfully employed before ends up causing metastasis of the tumor.

### Cellular response to sterile injury

#### Summary:

A protective response against a breach of the epidermis by sterile injury is a necessity for all multicellular organisms, and is likely to have evolved even earlier than the defense mechanisms against infections by foreign particles. However, for an insect larva in the wild, a breach of epidermis is likely to be accompanied by an infection, unlike the sterile needle injuries carried out in laboratory settings. The response to an aseptic injury is therefore a combination of wound healing and immune challenge anticipation. The reaction to injury bears remarkable resemblance to the mammalian “inflammatory response,” although tissue types such as the vascular system that are critical in the mammalian context are missing in arthropods.

As the mechanistic details of injury response in *Drosophila* are deciphered, it has become increasingly clear that hemocytes play an important role in this process (Rizki and Rizki 1992; Rämet *et al.* 2002; Goto *et al.* 2003; Márkus *et al.* 2005; Bidla *et al.* 2007; Ayres and Schneider 2008; Babcock *et al.* 2008; Lindgren *et al.* 2008; Pastor-Pareja *et al.* 2008; Defaye *et al.* 2009; Nam *et al.* 2012; Chakrabarti *et al.* 2016). Some of the seminal work on this topic comes from injury studies in the embryo (Wood *et al.* 2002, 2006; Mace *et al.* 2005; Stramer *et al.* 2005). The first molecular response to wounding is the propagation of a pulse of calcium at the wound site immediately following injury (Razzell *et al.* 2013). This activates the NADPH oxidase enzyme Dual oxidase (DUOX) to produce hydrogen peroxide (H_2_O_2_) (Moreira *et al.* 2010; Razzell *et al.* 2013). Circulating hemocytes respond to this H_2_O_2_ signal by migrating to the wound site (Wood *et al.* 2006; Moreira *et al.* 2010; Razzell *et al.* 2013). Once there, plasmatocytes phagocytose damaged cells (Stramer *et al.* 2005).

A similar scenario is observed for larval injuries, where the emphasis thus far is largely on the homing of hemocytes and their role in resolving the wound (Goto *et al.* 2003; Babcock *et al.* 2008; Shibata *et al.* 2017). Here, plasmatocytes are attracted to the wound site, where they phagocytose damaged cells and secrete factors that help form a clot to prevent leakage of the hemolymph (Goto *et al.* 2003; Babcock *et al.* 2008; Shibata *et al.* 2017). Crystal cells rupture and release prophenoloxidase enzymes and proteases such as Mp2/Sp7/PAE1, which also contribute to the formation of melanin (Castillejo-López and Häcker 2005; Bidla *et al.* 2007; Binggeli *et al.* 2014; Dudzic *et al.* 2015). This aids in protein cross-linking and in the formation of a hardened clot that aids the reepithelialization of the wound (Galko and Krasnow 2004).

#### Specifics:

JNK signaling is required for crystal cell rupture after injury (Bidla *et al.* 2007). The melanization reaction generates ROS as a by-product (Nappi *et al.* 1995) that functions as a systemic wound signal to activate protective pathways such as JNK signaling in the brain (Nam *et al.* 2012). As a late response to wounding, blood cells in circulation differentiate into lamellocytes (Márkus *et al.* 2005). While no function has been attributed to these lamellocytes in wound healing, puncturing the cuticle with a sterile needle has obvious resemblance to a breach of the epidermis by the wasp ovipositor, and could have evolved in anticipation of events that follow in the wild. Sterile injury also activates JAK/STAT signaling in the circulating blood cells, fat body, and wound site, and this activation depends on JNK signaling (Pastor-Pareja *et al.* 2008). The Toll pathway is activated in the fat body after injury, but this activation does not require blood cells (Kenmoku *et al.* 2017).

The role of blood cells in the inflammatory response has been studied by causing injuries using a variety of techniques, including puncture of the epidermis with a sterile needle, pinching, cutting, laser-wounding, the injection of foreign substances, and by feeding damage-inducing chemicals (Rizki and Rizki 1992; Rämet *et al.* 2002; Wood *et al.* 2002, 2006; Goto *et al.* 2003; Galko and Krasnow 2004; Mace *et al.* 2005; Márkus *et al.* 2005; Stramer *et al.* 2005; Bidla *et al.* 2007; Ayres and Schneider 2008; Babcock *et al.* 2008; Lindgren *et al.* 2008; Pastor-Pareja *et al.* 2008; Amcheslavsky *et al.* 2009; Chatterjee and Ip 2009; Defaye *et al.* 2009; Jiang and Edgar 2009; Nam *et al.* 2012; Chakrabarti *et al.* 2016; Kenmoku *et al.* 2017). Hemocytes migrate to a wound site within minutes and continue to accumulate up to 3 hr postinjury (Stramer *et al.* 2005; Wood *et al.* 2006; Babcock *et al.* 2008). Following migration, the hemocytes experience a refractory period lasting a few hours, during which time they cannot migrate to the site of a subsequent wound (Weavers *et al.* 2016b). In the embryo, directed migration toward the wound site is essential, but larval hemocytes are freely circulating and can arrive at the wound site by direct capture from the hemolymph (Babcock *et al.* 2008).

It was first noted in zebrafish that macrophages are attracted to a wound site by a H_2_O_2_ signal and that this chemotaxis requires the Src family kinase Lyn (Niethammer *et al.* 2009; Yoo *et al.* 2011). This process is remarkably conserved as chemotaxis of hemocytes in *Drosophila* also involves a H_2_O_2_ signal and Src42a (Stramer *et al.* 2005; Moreira *et al.* 2010; Razzell *et al.* 2013; Evans *et al.* 2015). In embryos, hemocyte migration to the wound requires Rac- and Rho-dependent formation of lamellipodial protrusions, but the Rho-family protein Cdc42 is dispensable (Stramer *et al.* 2005). Embryonic hemocytes also require PI3K to migrate toward wounds (Wood *et al.* 2006).

In larvae and adults, the hemolymph near the wound site coagulates into a plug or clot shortly after initial bleeding from an injury, which prevents further bleeding. This clot is composed of proteins secreted by both plasmatocytes and the fat body (Goto *et al.* 2003; Scherfer *et al.* 2004; Lindgren *et al.* 2008; Wang *et al.* 2010; Shibata *et al.* 2017). For example, Hml is an abundant clot protein in *Drosophila* that contains domains similar to those found in the human clotting protein von Willebrand factor (Goto *et al.* 2003; Scherfer *et al.* 2004). Blood cells also secrete transglutaminase, the fly homolog of the human coagulation factor VIII, which cross-links clot proteins and can entrap microbes introduced during injury (Lindgren *et al.* 2008; Wang *et al.* 2010; Shibata *et al.* 2017).

Once formed, the clot proteins are then melanized to form a dark, stabilized clot that is necessary for proper wound healing (Rämet *et al.* 2002; Galko and Krasnow 2004; Bidla *et al.* 2005, 2007; Nam *et al.* 2012). This process depends on the rupture of crystal cells and dissolution of their crystalline inclusions, which releases ProPOs that are activated by proteolytic cleavage, ultimately leading to deposition of melanin at the wound site (Bidla *et al.* 2005, 2007). Crystal cells rupture in response to the apoptosis of hemocytes and the melanization cascade requires the activation of the JNK pathway through Egr (Bidla *et al.* 2007). Mutants that disrupt the melanization cascade fail to survive after severe wounding as the wounded epidermis fails to close properly, and this demonstrates the beneficial function of prophenoloxidase activity in wound healing (Braun *et al.* 1998; Rämet *et al.* 2002; Galko and Krasnow 2004; Bidla *et al.* 2005; Ayres and Schneider 2008; Nam *et al.* 2012).

As mentioned above, the NADPH enzyme DUOX generates ROS at the wound site (Razzell *et al.* 2013). The PO-induced melanization reaction that occurs after injury produces additional ROS as a byproduct (Nam *et al.* 2012). Overexpression of ROS scavengers (Jafrac1 or Irc) or feeding of an antioxidant chemical (N-acetylcysteine) decreases survival after needle puncture, suggesting a positive role for ROS in injury response. Consistent with this notion, the injury-induced lethality in melanization mutants (*Bc* or *Hayan^1^)* is rescued by knockdown of *Jafrac1*, exposure to the prooxidant chemical paraquat, or the overexpression of PO (Nam *et al.* 2012).

It is well known that JNK activation at the injury site is key for wound healing (Rämet *et al.* 2002; Galko and Krasnow 2004). In addition, epidermal injury causes a systemic effect that induces JNK in the brain. The neural activation of JNK is dependent on ROS and the function of the melanization-promoting Hayan protein (Nam *et al.* 2012). Taken together, these data suggest a model in which ROS, generated as a byproduct of the melanization process mediated by Hayan, lead to JNK activation in the brain, which ultimately improves survival following epidermal injury.

The JAK/STAT pathway is also involved in epidermal injury response. Sterile injury causes plasmatocytes to secrete Upd ligands that activate the Dome receptor and the JAK/STAT pathway in the fat body. As a consequence, this organ expresses the downstream target protein Turandot A, which is a humoral factor that plays a role in stress tolerance (Chakrabarti *et al.* 2016). When Upd cytokine production is knocked down in plasmatocytes or when plasmatocytes are depleted by expression of a proapoptotic gene, flies become more susceptible to death from injury (Defaye *et al.* 2009; Chakrabarti *et al.* 2016). Thus, it is apparent that *Drosophila* blood cells respond to injury in multiple ways and that a hemocyte response is critical for the degree of postinjury survival.

### Cellular response to wasp parasitization

#### Summary:

**Figure 7:**
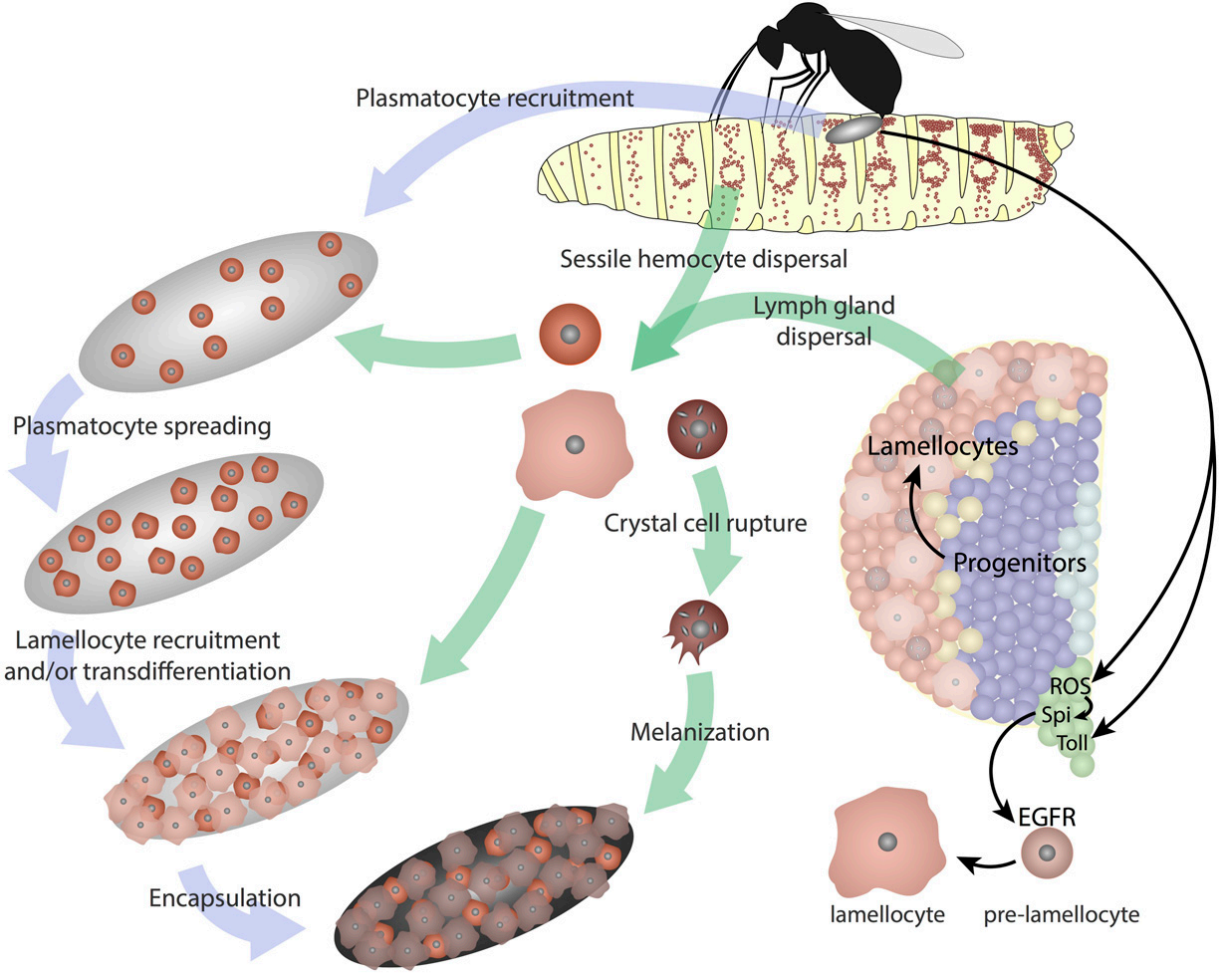
Larval hemocyte response to wasp infestation. Egg deposition beneath the larval cuticle by a parasitic wasp (top right) initiates a systemic signal that increases ROS levels in the cells of the PSC that causes Rhomboid and Spitz (Spi) activation. Secreted Spitz activates EGFR and promotes lamellocyte formation. The lymph gland disperses prematurely, and sessile hemocytes are dislodged from the cuticle and increase the number of mature hemocytes in circulation. Plasmatocytes adhere and spread along the outer shell of the wasp egg. Lamellocytes encapsulate and sequester the plasmatocyte-coated egg. Finally, crystal cell rupture aids in the melanization of the lamellocyte-encapsulated egg. This succession of events allows for the successful neutralization of the parasitic eggs in a fraction of infestation attempts. PSC, posterior signaling center; ROS, reactive oxygen species.

Historically, the role of hemocytes in the immune response was studied most extensively in the context of infestation by wasp eggs deposited beneath the larval cuticle. Some 50 or so hymenopteran species are reported to parasitize *Drosophila* (Carton *et al.* 1986). The best characterized is *Leptopilina boulardi*, which is a specialist parasite for *Drosophila melanogaster* (Carton and Nappi 1991). During wasp infestation, female wasps use an ovipositor to insert an egg into a first- or second-instar *Drosophila* larva (Rizki and Rizki 1991; Lanot *et al.* 2001). In the absence of a properly mounted host defense, these eggs proceed through their developmental stages, using the fly as their nutritional source. The ovipositor deposits chemical agents to restrict the immune response in some cases, but the process of egg deposition itself initiates a cascade of events by which the larva attempts to neutralize the wasp egg through encapsulation before the fly reaches the pupal stage (Labrosse *et al.* 2005a) (Figure 7). Fly hemocytes are crucial for the resulting immune response [reviewed in Letourneau *et al.* (2016)]. In fact, the fly involves its entire hematopoietic repertoire in its attempt to counter wasp parasitization (Russo *et al.* 1996). The breach of the epidermis by the ovipositor induces local changes that are systemically sensed by the PSC of the lymph gland as oxidative stress. Circulating lamellocytes that aid in the encapsulation of the parasitic wasp egg are induced by the PSC cells through a mechanism that involves ROS and the *Drosophila* Epidermal Growth Factor-like protein Spitz (Sinenko *et al.* 2011; Louradour *et al.* 2017).

Progenitors of the lymph gland differentiate, the circulating hemocyte numbers increase, and much of the lymph gland disintegrates earlier than it would without parasitization [reviewed in Letourneau *et al.* (2016)]. Plasmatocytes are the initial responders to foreign invasion, as they are the first to recognize and bind the wasp egg. Then, lamellocytes in circulation encapsulate the plasmatocyte-coated egg and form septate junctions between themselves (Russo *et al.* 1996; Anderl *et al.* 2016). If they are successful, the final step of the parasite-neutralization process involves the melanization reaction triggered by prophenoloxidase enzymes from crystal cells and lamellocytes (Irving *et al.* 2005; Dudzic *et al.* 2015).

#### Specifics:

One of the most severe naturally occurring immune challenges a fly larva faces is infestation by parasitoid wasps, which lay eggs in larvae and cause death in the absence of an effective cellular immune response [reviewed in Letourneau *et al.* (2016)] (Figure 7). As the first line of immune supervision, plasmatocytes respond to the wasp egg by elevating intracellular calcium levels. This process activates the plasmatocytes and promotes their attachment to the chorion of the egg 2–10 hr postwasp attack (Russo *et al.* 1996; Mortimer *et al.* 2013; Bajgar *et al.* 2015; Anderl *et al.* 2016). Later, a strong cellular reaction is seen with enhanced hemocyte proliferation ∼6 hr after egg deposition. Differentiation of lamellocytes within the circulating hemolymph and the larval lymph gland begins ∼10 hr after attack (Rizki and Rizki 1979, 1983, 1991; Lanot *et al.* 2001; Sorrentino *et al.* 2002; Crozatier *et al.* 2004; Makki *et al.* 2010; Sinenko *et al.* 2011). The lamellocytes, in particular, have the ability to encapsulate the foreign egg and prevent it from hatching. The lymph glands of parasitized animals prematurely disperse during the third instar (24–48 hr postwasp attack) to release lamellocytes that aid in this mode of defense (Lanot *et al.* 2001; Sorrentino *et al.* 2002; Louradour *et al.* 2017). Hemocytes in sessile clusters are also released into circulation beginning 27–29 hr after infestation (Zettervall *et al.* 2004; Markus *et al.* 2009; Vanha-Aho *et al.* 2015).

During encapsulation, the plasmatocyte-coated egg is surrounded by successive layers of lamellocytes that rapidly develop septate junctions between themselves, sequestering the wasp egg from the surrounding hemocoel (Russo *et al.* 1996; Lanot *et al.* 2001). The accumulation of lamellocytes around the egg is accompanied by flattening of the different blood cell layers, which are then melanized to further isolate, immobilize, and kill the parasite (Russo *et al.* 1996). Crystal cells and lamellocytes play a role in this process of melanization, both contributing PO activity encoded by the genes *PPO2* and *PPO3*, respectively (Irving *et al.* 2005; Binggeli *et al.* 2014; Dudzic *et al.* 2015).

Parasitized larvae begin expressing genes associated with molting and pupariation later than nonparasitized controls, and, accordingly, pupal development is delayed by ∼7 hr (Wertheim *et al.* 2005; Bajgar *et al.* 2015). Metabolic labeling experiments with ^14^C-glucose reveal that only one-tenth of the ^14^C is distributed into blood cells under normal growth conditions, but this increases dramatically to almost one-third of the glucose utilized following wasp parasitization. This is suggestive of a significant bioenergetic requirement within the hemocytes for the cellular immune response. After wasp infestation, blood cells in circulation and in the lymph gland upregulate the expression of glucose and trehalose transporters, and glycolytic genes. In contrast, the expression of these genes is partially repressed in the fat body. This metabolic shift of the blood cells at the expense of the other larval organs requires reception of an adenosine signal secreted by blood cells. A loss-of-function mutation in *AdoR* reduces this shift, decreases lamellocyte formation, and rescues the delay in pupariation (Bajgar *et al.* 2015). This metabolic shift in hemocytes is also observed after bacterial infection with *Streptococcus pneumoniae* and *Listeria monocytogenes* (Bajgar and Dolezal 2018).

Interestingly, the increase in lamellocyte differentiation in circulation is orchestrated by the PSC of the lymph gland. Loss of PSC cells in a *collier* mutant background or by expression of the proapoptotic protein Reaper prevents differentiation of lamellocytes following wasp infestation (Crozatier *et al.* 2004; Benmimoun *et al.* 2015). Genetic dissection of the pathway that facilitates the response to wasp infestation suggests that elevation of ROS within the PSC is a key triggering event (Sinenko *et al.* 2011). During normal development, PSC cells have undetectable levels of ROS. However, upon wasp parasitization, ROS levels rise dramatically, and scavenging ROS in the PSC using Superoxide dismutase 2 suppresses lamellocyte formation within the lymph gland and in circulation. Artificially increasing ROS in the PSC cells by altering metabolic activity increases the number of lamellocytes in circulation and the lymph gland in the absence of wasp infestation (Sinenko *et al.* 2011). High ROS activates the Toll pathway in PSC cells, which contributes to lamellocyte induction in response to wasp parasitization (Louradour *et al.* 2017). Toll activity reporters demonstrate that this pathway is active in both the PSC and the rest of the primary lobe after wasp infestation (Gueguen *et al.* 2013; Louradour *et al.* 2017). Loss of Toll signaling in *Dif* and *pelle* mutants impairs the formation of lamellocytes and the encapsulation of parasitic wasp eggs (Louradour *et al.* 2017). In parallel, Spitz/EGFR signaling is also an essential component downstream of ROS within the PSC that mediates lamellocyte differentiation. The proteins Star and Rhomboid, necessary for trafficking and cleavage of the EGFR ligand Spitz, are essential for lamellocyte induction. EGFR activity is elevated within circulating cells, including lamellocytes, following parasitization, and a dominant negative form of EGFR expressed in the hemocytes suppresses lamellocyte formation (Sinenko *et al.* 2011).

Genes that affect the maintenance and differentiation of prohemocytes also affect lamellocyte differentiation during wasp infestation. For example, Collier expression is required within lymph gland progenitors for their maintenance (Benmimoun *et al.* 2015), and downregulation of Collier in progenitors is essential for lamellocyte differentiation and lymph gland dispersal following wasp parasitization (Oyallon *et al.* 2016). Similarly, JAK/STAT signaling in prohemocytes affects progenitor maintenance and the response to wasp infestation (Krzemień *et al.* 2007). Latran binds the Domeless receptor to form a heterodimeric complex and prevents signaling through the JAK/STAT pathway (Kallio *et al.* 2010). When larvae are parasitized by wasps, the expression of Latran increases, which inhibits JAK/STAT signaling and promotes the ability of progenitors to form lamellocytes. In *latran* mutant larvae, JAK/STAT signaling remains active in progenitors and lamellocytes are less frequently induced upon parasitization (Makki *et al.* 2010). In an interesting twist, insulin and JAK/STAT signaling are also both required in muscles for the formation of lamellocytes and the encapsulation response upon wasp parasitization. This immune response is nutritionally controlled by carbohydrate availability and involves the muscle as a target organ, but not the fat body or hemocytes (Yang and Hultmark 2017)

In addition to hematopoietic factors that affect lamellocyte differentiation, a number of external signals affect the cellular immune response to wasp parasitization. For example, ecdysone plays a role in the lymph gland response to wasp parasitization, and induces premature lymph gland dispersal and lamellocyte differentiation (Sorrentino *et al.* 2002). Both lymph gland dispersal and lamellocyte formation are inhibited in the *ecdysoneless* (*ecd*) mutant. Furthermore, both the *ecd* mutant and a mutant of the Broad Complex, a downstream regulator of ecdysone signaling, have decreased encapsulation ability (Sorrentino *et al.* 2002). Secretion of Edin (Elevated during infection) from the fat body promotes wasp egg encapsulation, not by altering the number of circulating lamellocytes, but by affecting the encapsulation process itself (Vanha-Aho *et al.* 2015). Knockout of the complement-like thioester-containing proteins (Tep1–4) suppresses the effectiveness of the immune response to wasp parasitization (Dostálová *et al.* 2017). Additionally, several factors are injected by the wasp that suppress the cellular immune response. For example, a factor released during *L. heterotoma* infestation causes lysis of lamellocytes and thus prevents encapsulation (Rizki and Rizki 1984). Similarly, *L. boulardi* and other species utilize RhoGAP proteins to interfere with the cytoskeleton of lamellocytes through inhibition of the Rho GTPases Rac1 and Rac2. This changes lamellocyte morphology and adhesive properties, which also inhibits effective encapsulation (Reineke *et al.* 2002; Labrosse *et al.* 2005a,b; Colinet *et al.* 2007, 2013). *L. boulardi* venom also contains two inhibitors of PO activity, a serpin and an extracellular superoxide dismutase enzyme that scavenges ROS (Colinet *et al.* 2009, 2011, 2013). Effective wasp egg encapsulation also requires an elevated level of intracellular calcium in plasmatocytes. *Ganaspis sp.1* blocks this process by utilizing a protein component in its venom that is homologous to SERCA (sarco/ER calcium ATPase) (Mortimer *et al.* 2013). Several wasp species including *Culicoides sonorensis* and *Microplitis demolitor* inject viral ankyrin proteins similar to *Drosophila* IκB (Cactus), which can bind to NF-κB proteins and suppress Toll activation, thus preventing lamellocyte differentiation and encapsulation of wasp eggs (Bitra *et al.* 2012; Gueguen *et al.* 2013).

### Cellular response to pathogens and septic injury

#### Summary:

In addition to wasp parasitization, *Drosophila* is infected by many pathogens including bacteria and fungi. *Drosophila* innate immunity is best characterized as a humoral response in adult flies that is initiated by the fat body, the arthropod equivalent of the mammalian liver and adipose tissue. The extensive body of literature devoted to these studies is addressed elsewhere [reviewed in Hoffmann and Reichhart (2002), Brennan and Anderson (2004), Wasserman (2004), Senger *et al.* (2006), Lemaitre and Hoffmann (2007), and Ganesan *et al.* (2011)]. These studies in *Drosophila* were critical to our understanding of the innate immune response in any organism, including humans. In brief, infection by gram-positive bacteria or fungi activates Toll signaling in the fat body, while gram-negative infections activate the Immune deficiency (Imd) pathway in this organ. These signals act through different transcription factors of the NF-κB family to induce the expression of downstream immune response genes, especially several AMPs that are secreted into the hemolymph and allow flies to resist infections. Therefore, *Toll* and *Imd* pathway mutants are very susceptible to infection with many different microbes. Blood cells also secrete AMPs, but the humoral response is clearly dominant in the context of infection. Nevertheless, it is becoming increasingly clear that blood cells do play additional key roles as sentinels of antimicrobial immunity. In this section, we only focus on the less-studied, hemocyte-related cellular components of the immune response to bacterial and fungal infections.

The early literature on innate immunity is virtually silent on any role that hemocytes might play in this process, largely based on studies that involve mutants lacking a majority (but not all) of larval blood cells, such as *lethal (3) hematopoietic organs missing* (*l(3)hem*) and *domino* (Gateff 1994; Braun *et al.* 1997). These mutations show pleiotropic defects in multiple organs, including impaired development of imaginal discs and neuroblasts, and are lethal during pupal stages (Gateff 1994; Braun *et al.* 1997). However, the larvae have normal fat body immune response to fungi, and gram-positive and -negative bacteria (Braun *et al.* 1998). This observation led to the suggestion that hemocytes play an insignificant, if any, role in the innate immune response. On the other hand, double-mutant larvae lacking both *domino* function, and either Toll or Imd activity, show enhanced susceptibility to bacterial and fungal infections relative to either mutant alone, suggesting a synergy between blood cells and the systemic immune response (Braun *et al.* 1998). Furthermore, blood cells contribute specialized functions, which generally act on faster timescales than the humoral response (Kocks *et al.* 2005; Bidla *et al.* 2007; Babcock *et al.* 2008; Yano *et al.* 2008). Mutational studies of *domino* and *l(3)hem* mutants are complicated by the fact that all vestiges of the hematopoietic repertoire are not fully eliminated, and the remaining mature hemocytes can divide to increase their population in response to stress. Many of these possibilities are being addressed in newer studies and this subject is likely to remain a field of active research in the near future.

Targeted ablation of plasmatocytes by driving the expression of proapoptotic genes depletes the vast majority of hemocytes in the animal with limited side effects on other organs (Charroux and Royet 2009; Defaye *et al.* 2009; Shia *et al.* 2009). This manipulation results in higher bacterial load and decreased survival following septic infection with both gram-positive and -negative bacteria (Charroux and Royet 2009; Defaye *et al.* 2009; Shia *et al.* 2009). Decreased survival after infection is largely dependent upon phagocytosis, since knockdown of phagocytosis receptors in plasmatocytes causes a similar phenotype (Charroux and Royet 2009; Defaye *et al.* 2009; Shia *et al.* 2009; Guillou *et al.* 2016). The phagocytosis-defective mutants represent convincing evidence for the participation of plasmatocytes in the *Drosophila* immune response.

In addition, analysis of crystal cell function during infection provides compelling evidence for hemocyte-specific immunity. Loss-of-function mutations in enzymes involved in the melanization cascade initiated by crystal cells lead to increased susceptibility to septic infection with multiple species of fungi, and gram-positive and -negative bacteria (De Gregorio *et al.* 2002; Ayres and Schneider 2008; Binggeli *et al.* 2014; Dudzic *et al.* 2015). Melanization enzyme activity helps limit the postinfection bacterial load (Ayres and Schneider 2008; Binggeli *et al.* 2014). The perturbations used in these experiments are specific to a single cell type and involve mechanisms different from those used during the humoral response.

#### Specifics:

Many different bacterial and fungal species previously used in the context of humoral innate immunity in *Drosophila* are now being used to study cellular immune responses. With limited information on natural *Drosophila* pathogens, most past studies resorted to septic injury models, largely in adults involving strains such as the gram-negative bacterium *E. coli*, the gram-positive bacteria *Micrococcus luteus* or *Bacillus subtilis*, or the fungus *Candida albicans* that do not naturally infect *Drosophila*, but do so upon septic injury. Occasionally, plant pathogens such as *Agrobacterium tumefaciens* and *Erwinia carotovora* (both gram-negative) are used in septic injury experiments (Foley and O’Farrell 2003; Leclerc *et al.* 2006). Another common practice is to use human pathogens such as the gram-positive *Listeria monocytogenes*, *Mycobacterium marinum*, *Staphylococcus saprophyticus*, *Enterococcus faecalis*, *Staphylococcus aureus*, *Streptococcus pneumoniae*; the gram-negative species *Salmonella typhimurium*, *Enterobacter cloacae*, *Pseudomonas aeruginosa*; or the fungus *Aspergillus fumigatus* for septic injury (Lemaitre *et al.* 1997; D’Argenio *et al.* 2001; Dionne *et al.* 2003; Mansfield *et al.* 2003; Brandt *et al.* 2004; Needham *et al.* 2004; Pham *et al.* 2007). Infection by these human pathogens can lead to the death of wild-type flies despite activation of both the cellular and humoral responses.

Septic injury caused by a puncture wound continues to be a prevalent method used to induce an immune response in a laboratory setting, and this process does mimic the effects of infections in the wild that are followed by entry of pathogens into the hemolymph. However, increasingly, such studies have involved exposure to natural pathogens that invade through ingestion or the external cuticle. This allows the study of microbial infections in the absence of a laboratory-induced cooccurring injury response. When ingested, gram-negative *Serratia marcescens* (especially strain *Db11*) and *Pseudomonas entomophila* naturally infect fly larvae and adults, and usually kill the fly in spite of an evoked immune response (Basset *et al.* 2000; Vodovar *et al.* 2005; Nehme *et al.* 2007). On the other hand, *E. carotovora* strain 15 (*Ecc15*), which is also an ingested pathogen, persists in the gut without killing the flies, even though they mount an immune response (Basset *et al.* 2000).

Perhaps the most straightforward example of how hemocytes participate in innate immunity is obvious from studies involving crystal cells and lamellocytes, since unlike the plasmatocyte population, these cells do not have overlapping functions with the fat body. *Bc* mutant larvae and adults, which lack crystal cell-mediated melanization, display increased susceptibility to septic injury with the fungus *Beauveria bassiana* (Braun *et al.* 1998; Leclerc *et al.* 2006). *Bc* mutant animals also have increased susceptibility to the gram-negative bacteria *A. tumefaciens* and *E. coli*, and the gram-positive bacteria *E. faecalis* and *M. luteus* (Braun *et al.* 1998; Leclerc *et al.* 2006). Furthermore, a double knockout of *PPO1* and *PPO2* is more susceptible to septic injury with several species of fungi (*C. albicans* and *A. fumigatus*), gram-positive bacteria (*S. aureus*, *Li. monocytogenes*, *S. saprophyticus*, *E. faecalis*, and *B. subtilis*), and the gram-negative bacterium *E. carotovora*, but has only minor effects on survival after septic injury with several other gram-negative bacterial species (*S. typhimurium* and *E. cloacae*). These PPO1/PPO2 double mutants are also more susceptible to natural infection with fungal species such as *B. bassiana* and *Metarhizium anisopliae*, which invade fly larvae through the cuticle. These mutants also carry a higher bacterial load after *S. aureus* septic injury, implying that PO activity helps keep bacterial infection in check (Binggeli *et al.* 2014). This crystal cell-mediated immune response to fungal and bacterial infection is not related to AMP production, which remains unchanged in mutants incapable of melanization. Instead, PO-mediated ROS formation is the likely mechanism for this antimicrobial defense system (Tang *et al.* 2006; Nam *et al.* 2012; Binggeli *et al.* 2014).

Mutations in proteases that activate PO activity have also been used to study the role of melanization in defense against microbes. For example, Hayan is the protease that activates PPO1 and PPO2 through cleavage of these zymogens (Nam *et al.* 2012). Microbes introduced during septic injury enhance *Hayan* transcription in a Toll- and Imd-dependent manner (Nam *et al.* 2012). Two other proteases that play a role in PPO1 and PPO2 activation are Melanization protease 1 (MP1) and Melanization protease 2 (MP2, also known as PAE1 and Sp7). MP2 is specifically expressed in crystal cells and is upregulated in response to infection (Castillejo-López and Häcker 2005). Knockdown of *MP2* enhances lethality following fungal infection (Tang *et al.* 2006). Likewise, *MP2*-deficient animals are very susceptible to *S. typhimurium* (gram-negative) and *L. monocytogenes* (gram-positive) bacteria (Ayres and Schneider 2008). *MP1*- or *MP2*-deficient flies become even more susceptible to infections when combined with mutations in the Toll or Imd pathways, suggesting that the systemic response from the fat body synergizes with the hemocyte-derived cellular response to provide full immunity against microbes.

Excessive melanization in flies deficient in Serpin27A (Spn27A), a negative regulator of PO function, results in poorer survival outcomes than for wild-type flies when these flies encounter septic infection by *B. bassiana*, *E. coli*, *M. luteus*, or *A. fumigatus* (De Gregorio *et al.* 2002). PO activity is strictly regulated in homeostasis by Spn27A to prevent spontaneous melanization (De Gregorio *et al.* 2002; Ligoxygakis *et al.* 2002). When the need for active defense against pathogen infection arises, the activated Toll pathway downregulates Spn27A, and this facilitates the PO-mediated melanization response (Ligoxygakis *et al.* 2002). These results further demonstrate the interplay between the humoral and cellular response systems as an essential element of innate immunity.

Another unique property of the cellular immune response is exemplified by plasmatocyte function in combating opportunistic infection by bacteria or fungi present under normal culture conditions that do not negatively affect survival unless the immune system is compromised. Depletion of blood cells reduces the animal’s ability to survive such invasions and numerous bacteria are seen in the hemolymph (Braun *et al.* 1998; Matova and Anderson 2006). Similarly, specific depletion of plasmatocytes through expression of proapoptotic genes or mutation of the phagocytic receptor *croquemort* (*crq*) causes a decrease in survival to adulthood under normal culture conditions (Charroux and Royet 2009; Defaye *et al.* 2009; Shia *et al.* 2009; Guillou *et al.* 2016). This phenotype is reversed either by growing flies in axenic (germ-free) conditions or by including antibiotics in the culture medium. This emphasizes the importance of the phagocytic response to bacteria that are frequently ingested in the typical culture environment. This mechanism is especially important for infections by bacteria such as the gram-negative *S. marcescens*, which does not elicit a strong fat body immune response, and therefore phagocytosis plays a dominant role in combating this pathogen (Nehme *et al.* 2007).

Depletion of plasmatocytes from either larvae or adults increases susceptibility to septic infection with pathogens such as *S. aureus* and *E. faecalis* (gram-positive), *S. typhimurium* and *P. entomophila* (gram-negative), and *C. albicans* (fungus) (Jin *et al.* 2008; Charroux and Royet 2009; Defaye *et al.* 2009). When phagocytosis is blocked, either by injection of latex beads into the hemocoel or by mutations in *eater*, *nimrod*, *crq*, or *Rac2*, a similar decrease in survival is seen after infection with *S. aureus*, *M. luteus*, *E. faecalis*, *P. aeruginosa*, and *S. marascens* (Kocks *et al.* 2005; Avet-Rochex *et al.* 2007; Nehme *et al.* 2007; Schneider *et al.* 2007; Jin *et al.* 2008; Charroux and Royet 2009; Defaye *et al.* 2009; Nehme *et al.* 2011; Guillou *et al.* 2016; Bajgar and Dolezal 2018). Since blocking phagocytosis does not affect AMP induction in the fat body, this emphasizes the importance of cellular immunity in resisting infection (Elrod-Erickson *et al.* 2000; Kocks *et al.* 2005; Charroux and Royet 2009). Indeed, *P. aeruginosa* directly inhibits phagocytosis through injection of the virulence factor ExoS toxin, a GAP domain-containing enzyme, which binds and inhibits Rac2 GTPase resulting in decreased survival (Avet-Rochex *et al.* 2005, 2007). When conditions that lead to a loss of phagocytosis are combined with mutations in the Toll or Imd pathways, the resulting larvae and adults display even greater susceptibility to septic injury relative to either defect alone (Braun *et al.* 1998; Elrod-Erickson *et al.* 2000; Nehme *et al.* 2007, 2011; Charroux and Royet 2009; Defaye *et al.* 2009). Additionally, chemically synthesized Cecropin A, an AMP normally secreted by the fat body after immune challenge, also enhances Eater binding to live bacteria (Chung and Kocks 2011). These observations point to a synergy between the humoral and cellular immune responses that provides optimal resistance to infection.

During oral infection of larvae with *Ecc15*, hemocytes are necessary to induce the AMP Diptericin (Dpt) in the fat body downstream of the Imd pathway (Basset *et al.* 2000; Foley and O’Farrell 2003; Dijkers and O’Farrell 2007; Charroux and Royet 2009; Shia *et al.* 2009). In this case, Calcineurin A1 is required in hemocytes to respond to an NO signal from the gut, which ultimately causes activation of the Imd pathway in the fat body and the transcription of downstream AMPs (Foley and O’Farrell 2003; Dijkers and O’Farrell 2007).

Both hemocytes and the fat body express Spatzle (Spz), a Toll ligand that is proteolytically cleaved to allow for Toll pathway activation; however, upon bacterial infection, hemocytes, but not the fat body, upregulate Spz (Irving *et al.* 2005). Hemocytes also upregulate PGRP-SA, a circulating pattern recognition receptor upstream of the Toll pathway in response to septic infection (Irving *et al.* 2005). These results suggest that hemocytes play a role in promoting Toll pathway activation. However, several studies have been conducted that have combined septic injury with depletion of hemocytes. The results using bacterial and fungal species have obtained different results. Most suggest no effect of hemocytes on AMP production (Braun *et al.* 1998; Basset *et al.* 2000; Matova and Anderson 2006; Charroux and Royet 2009; Defaye *et al.* 2009). However, one study using several different strategies for hemocyte depletion as well as hemocyte-specific depletion of Spz concluded that blood cells are indeed required for the induction of Dpt and Drosomycin (Shia *et al.* 2009). Furthermore, blood cells have also been shown to express Psidin (Phagocyte signaling impaired), which is specifically required for the induction of Defensin in the fat body following septic injury with *E. coli* (Brennan *et al.* 2007).

Unexpectedly, the lymph gland proper also plays a role in immunity against microbial infection. Bacterial challenge (such as by *E. coli* or *B. subtilis*) causes loss of proper septate junctions between the cells of the PSC (Khadilkar *et al.* 2017). This causes increased differentiation of plasmatocytes and crystal cells, and leads to premature dispersal of the lymph gland cells. Thus, disruption of PSC septate junctions during bacterial infection ultimately increases the number of circulating hemocytes in the larva that then continue into the adult. This improves the survival of adult flies subjected to *E. coli* and *B. subtilis* infection. While infection leads to increased plasmatocytes, overexpression of Cora or NrxIV in the PSC reforms the septate junctions, and thereby inhibits the increase in differentiation. Interestingly, Toll or Imd activation in the PSC also causes the perturbation of septate junctions between these cells in the absence of infection, and artificial activation of either the Toll or Imd pathways in the PSC leads to the breakdown of septate junctions within the PSC and increased plasmatocyte differentiation. Disrupting septate junctions in larvae increases the number of circulating hemocytes in adults. This improves the survival of adult flies after *E. coli* infection (Khadilkar *et al.* 2017).

As described for aseptic injury and wasp infestation, Upd3 is also activated in hemocytes in response to septic injury (for example with the gram-negative bacterium *Ecc15*) and consequently leads to the activation of the Dome/JAK/STAT pathway in multiple tissues, including the fat body, muscle, and gut (Agaisse *et al.* 2003; Chakrabarti *et al.* 2016; Yang and Hultmark 2017). As expected, knockdown of *upd3* in plasmatocytes causes increased susceptibility to septic injury (Chakrabarti *et al.* 2016). In the gut, activation of this pathway upon infection causes increased proliferation of intestinal stem cells, while in the fat body, active STAT cooperates with the Imd pathway leading to expression of the stress-response protein *Turandot A* (*TotA*) and the C3 complement-like protein Tep1 (Lagueux *et al.* 2000; Agaisse *et al.* 2003; Chakrabarti *et al.* 2016). Tep1–4 represent a set of thioester-containing proteins that act as opsonins and promote phagocytosis. Their loss renders flies more susceptible to infection (Stroschein-Stevenson *et al.* 2006; Dostálová *et al.* 2017). This process is akin to the human acute-phase response to infection in which blood cell cytokine signaling causes the liver to secrete stress-response proteins, such as the opsonin C-reactive protein that promotes the phagocytosis of microbes [reviewed in Cray *et al.* (2009)]. During oral infection with either *Ecc15* or *P. entomophila* (both gram-negative), plasmatocytes migrate to the gut and secrete Dpp, which is required for intestinal stem cell proliferation in response to infection (Ayyaz *et al.* 2015). These results indicate that hemocytes communicate with multiple tissues to restore homeostasis and mitigate stress caused by infection.

## Development and the Stress Response: Two Sides of the Same Coin

As a core principle of homeostasis, any tissue that experiences stress is expected to react in a local or systemic fashion to bring its function back to normalcy. The brain, for example, will secrete hormones to mitigate the effects of oxidative stress. However, signals responsible for the development of a specific tissue are typically distinct from the ones that are sent out to indicate distress. In contrast, independent studies over the years suggest that myeloid progenitors, at least in *Drosophila*, often make dual use of the same pathways during stress and homeostasis. It is not surprising that differentiated myeloid cells are particularly sensitive to environmental and systemic cues, given their innate immune function as proverbial watchmen that surveil the body for signs of trouble and respond to situations that threaten homeostasis such as injury, tissue damage, infection, and disease. What is interesting and novel is the realization that it is the quiescent multipotent progenitors, largely confined within the hematopoietic organ, that directly sense and respond to stress. This response involves a loss of quiescence as progenitors proliferate and differentiate, giving rise to hemocytes capable of stress mitigation. The circulating cells also respond to stress, but it is less clear whether this is due to the rapid amplification of existing blood cells or a *de novo* hematopoietic response.

Dual use of the same pathway for development and the stress response is commonplace within the myeloid system. The Toll and JAK/STAT pathways, critically responsible for innate immunity against bacteria, wasp eggs, aseptic injury, and epithelial tumors, are also important for blood cell differentiation and survival (Harrison *et al.* 1995; Qiu *et al.* 1998; Sorrentino *et al.* 2002, 2004; Zettervall *et al.* 2004; Matova and Anderson 2006; Senger *et al.* 2006; Krzemień *et al.* 2007; Makki *et al.* 2010; Minakhina *et al.* 2011; Yang *et al.* 2015; Chakrabarti *et al.* 2016; Louradour *et al.* 2017). In the lymph gland, a balance between progenitors and differentiated cells is maintained during larval homeostasis. The pathways employed for maintenance of the progenitor population are often adapted to alter this balance during stress. In addition to the use of well-characterized developmental pathways, more esoteric and evolutionarily ancient signaling molecules such as ROS, NO, adenosine, odorants, atmospheric gases, steroids, GABA, and amino acids are used as developmental signals, and reutilized as stress sensors.

We highlight some of the above strategies in this section. As mentioned earlier, the progenitor population physiologically generates higher levels of ROS than are seen in most other tissues during development, and fails to differentiate if this ROS is entirely scavenged. Thus, ROS are used as a trigger for differentiation during normal *Drosophila* hematopoiesis. This is convenient since during oxidative stress, ROS levels rise and the progenitor population can rapidly differentiate to expand the mature hemocyte pool to mitigate the effects of the oxidative stress (Owusu-Ansah and Banerjee 2009; Gao *et al.* 2013). As another example, ecdysone signaling initiates many of the transitions in the controlled growth of the larva, including that of the lymph gland, and is also essential for the dispersal and activation of lymph gland cells during pupariation (Sorrentino *et al.* 2002; Regan *et al.* 2013). In addition, this same pathway is then used to minimize the effects of wasp parasitization (Sorrentino *et al.* 2002).

An interesting aspect of dual use is seen for the function of the InR pathway, which is critical for progenitor maintenance during development. Loss of insulin signaling causes loss of the progenitor population through the action of the PI3K/Akt/TOR pathways (Benmimoun *et al.* 2012; Dragojlovic-Munther and Martinez-Agosto 2012; Shim *et al.* 2012; Tokusumi *et al.* 2012). The same pathway combination senses a loss of nutrients, largely amino acids, as a stress signal during starvation, and reacts in multiple ways to mount an immune response and also to utilize fat cells for survival (Dragojlovic-Munther and Martinez-Agosto 2012; Shim *et al.* 2012).

An unusual form of Notch signaling in the context of hypoxia involves the formation of a Notch/Hif-α (Sima in *Drosophila*) complex that activates specialized target response elements (Mukherjee *et al.* 2011). This mechanism is utilized to maintain the integrity of crystal cells under normoxic conditions. This is achieved by stabilizing Hif-α using the normally high NO levels in crystal cells when the O_2_ levels are normal (Mukherjee *et al.* 2011). If, in addition, the animal encounters hypoxia, or an imbalance between CO_2_ and O_2_, additional amounts of Hif-α are stabilized and this causes an increase in the crystal cell population (Mukherjee *et al.* 2011; Cho *et al.* 2018). Large variations in O_2_ and CO_2_ concentrations are encountered as the larva digs deep into food, particularly when attracted by yeast. By mechanisms not yet clear, the increased numbers of crystal cells provide protection during hypoxia or other gaseous imbalances.

The *Drosophila* hematopoietic system receives sensory information from the outside world and also reacts to it using an elegant strategy of dual control (Dragojlovic-Munther and Martinez-Agosto 2012; Shim *et al.* 2012, 2013; Cho *et al.* 2018). The progenitor population of the MZ is maintained in its relatively quiescent state by a G protein-coupled GABA-B receptor (GABA_B_R) system that creates a proper balance between the levels of cytosolic and ER-stored Ca^2+^ ions. During homeostasis, proper calcium levels are maintained by binding of the neurotransmitter GABA to its receptor expressed in the cells of the MZ. Interestingly, the hemolymph of the fly contains significant levels of secreted GABA, similar to the blood serum of humans. The secretion of GABA into the hemolymph is under the control of an olfactory receptor that detects fruity odors. Thus, during homeostasis, appropriate levels of odorant ligands in the immediate vicinity of the larva maintain appropriate GABA levels in its circulating blood to signal favorable conditions for progenitor maintenance in the MZ during development. Strikingly, a genetically altered larva unable to smell, or a normal larva suffering odor deprivation, has a very strong immune response due to a decrease in cytosolic calcium in the MZ of the lymph gland, which causes a loss of progenitor quiescence and increased differentiation (Shim *et al.* 2013). Thus, the same Ca^2+^-dependent mechanism used during development to maintain progenitors is reused to overcome sensory stress by rapidly expanding the hematopoietic repertoire.

It is known that intrinsic, oxidative, infectious, systemic, and environmental stresses elicit strong reactions by the human myeloid system [reviewed in Muralidharan and Mandrekar (2013)]. However, due to the complexity of the system, the lack of appropriate genetic tools, and the involvement of multiple organs in the process, it is extremely difficult to establish the mechanistic steps that underlie the causal link between a stress and the resulting myeloid response. The *Drosophila* studies suggest that conserved developmental and stress-related pathways may be more interrelated than previously appreciated. We hope that such studies will help model the molecular mechanisms underlying the human myeloid system during normal development and stress.

## Final Words

The classical biological literature on invertebrate blood cells has created a solid foundation on which current studies on molecular genetic mechanisms rest. The Dipteran lymph gland was first identified and named as such by Weismann in the early 1860s [described in Weismann (1864)], but its function remained controversial over a century later [discussed in Shrestha and Gateff (1982)]. The blood cell types in *Drosophila*, and aspects of their development and function, including immune defense against wasp parasitization, were all very carefully cataloged by the venerable team work of Tahir M. Rizki and Rose M. Rizki. T. Rizki received his early education in Afghan-India and, during his career spanning well over three decades, mostly at the University of Michigan, he published 129 seminal papers, more than one-half of which are coauthored with Rose Rizki. It is fairly clear that Rizki and Rizki are the founders of this field, as is Elisabeth Gateff, a long-time former Director of the Max Planck Institute in Mainz, Germany, who analyzed the ultrastructure of blood cells and isolated a large collection of mutants that affect the hematopoietic system, ushering the field into the modern era. In this context, it is noteworthy to mention Istvan Ando at the Hungarian Academy of Sciences in Szeged and Dan Hultmark at Umea, Sweden, who during the early days of research in this field, collaboratively developed antibody markers and identified the corresponding genetic loci respectively, and facilitated all future mechanistic genetic analysis within the field. By no means is this meant to be a comprehensive list, even from a historical perspective. The combined effort of many laboratories has now led to a reasonably detailed picture of the hematopoietic process at different developmental stages. The authors of this chapter have attempted to include as much information as possible within the constraints of a publication. We apologize in advance to all those whose important research was not included in this chapter.

The virtues of using *Drosophila* as a model system to provide hints about human diseases have been eloquently stated elsewhere. This review has a narrower goal: to present a compilation of results that explore the full potential of the *Drosophila* model system in providing a *bona fide* tool kit that will help understand human blood cell development and function at a molecular level. Many new insights, concepts, and pathways have already emerged from molecular genetic analyses of *Drosophila* hematopoiesis. As with most other studies that have successfully exploited genetic dissection as a strategy to understand development, the analogy and similarity of *Drosophila* blood to the mammalian hematopoietic system is expected to be at the level of fundamental principles. The logic and algorithm that controls the system, as well as the conserved molecular networks that support the developmental strategies, are likely to be more important than the exact matches or lack thereof in cell types that have been selected during evolution due to their specific adaptive functions. For example, it is no doubt convenient and useful to point to the similarities of plasmatocytes to macrophages; of crystal cells to platelets and granulocytes; or of lamellocytes to giant cells. However, the more useful analogy is in the conservation of specific transcription factors as well as signaling pathways involved in the construction of a hematopoietic system. In this review alone, we have explored dozens of such conserved signaling components and specific transcription factors between flies and humans. Also important is the conservation of function, such as phagocytosis, innate immunity, wound healing, gas sensing, and engulfment of large particles, that is shared with the mammalian blood system. A full understanding of the *Drosophila* hematopoietic system is still in its infancy, but given the rapid progress in recent years, we expect this system to facilitate breakthroughs in our understanding of myeloid cell development and function in humans.

## References

[bib1] Abel T. Michelson A. M. Maniatis T., 1993 A Drosophila GATA family member that binds to Adh regulatory sequences is expressed in the developing fat body. Development 119: 623–633. 8187633 10.1242/dev.119.3.623

[bib2] Agaisse H. Petersen U. M. Boutros M. Mathey-Prevot B. Perrimon N., 2003 Signaling role of hemocytes in Drosophila JAK/STAT-dependent response to septic injury. Dev. Cell 5: 441–450. 10.1016/S1534-5807(03)00244-2 12967563

[bib3] Alfonso T. B. Jones B. W., 2002 gcm2 promotes glial cell differentiation and is required with glial cells missing for macrophage development in Drosophila. Dev. Biol. 248: 369–383. 10.1006/dbio.2002.0740 12167411

[bib4] Amcheslavsky A. Jiang J. Ip Y. T., 2009 Tissue damage-induced intestinal stem cell division in Drosophila. Cell Stem Cell 4: 49–61. 10.1016/j.stem.2008.10.016 19128792 PMC2659574

[bib5] Anderl I. Vesala L. Ihalainen T. O. Vanha-Aho L. M. Ando I., 2016 Transdifferentiation and proliferation in two distinct hemocyte lineages in Drosophila melanogaster larvae after wasp infection. PLoS Pathog. 12: e1005746 10.1371/journal.ppat.1005746 27414410 PMC4945071

[bib6] Arendt D. Tessmar-Raible K. Snyman H. Dorresteijn A. W. Wittbrodt J., 2004 Ciliary photoreceptors with a vertebrate-type opsin in an invertebrate brain. Science 306: 869–871. 10.1126/science.1099955 15514158

[bib7] Asha H. Nagy I. Kovacs G. Stetson D. Ando I., 2003 Analysis of Ras-induced overproliferation in Drosophila hemocytes. Genetics 163: 203–215. 12586708 10.1093/genetics/163.1.203 PMC1462399

[bib8] Avet-Rochex A. Bergeret E. Attree I. Meister M. Fauvarque M. O., 2005 Suppression of Drosophila cellular immunity by directed expression of the ExoS toxin GAP domain of Pseudomonas aeruginosa. Cell. Microbiol. 7: 799–810. 10.1111/j.1462-5822.2005.00512.x 15888083

[bib9] Avet-Rochex A. Perrin J. Bergeret E. Fauvarque M. O., 2007 Rac2 is a major actor of Drosophila resistance to Pseudomonas aeruginosa acting in phagocytic cells. Genes Cells 12: 1193–1204. 10.1111/j.1365-2443.2007.01121.x 17903178

[bib10] Avet-Rochex A. Boyer K. Polesello C. Gobert V. Osman D., 2010 An in vivo RNA interference screen identifies gene networks controlling Drosophila melanogaster blood cell homeostasis. BMC Dev. Biol. 10: 65 10.1186/1471-213X-10-65 20540764 PMC2891661

[bib11] Ayres J. S. Schneider D. S., 2008 A signaling protease required for melanization in Drosophila affects resistance and tolerance of infections. PLoS Biol. 6: 2764–2773. 10.1371/journal.pbio.0060305 19071960 PMC2596860

[bib12] Ayyaz A. Li H. Jasper H., 2015 Haemocytes control stem cell activity in the Drosophila intestine. Nat. Cell Biol. 17: 736–748. 10.1038/ncb3174 26005834 PMC4449816

[bib13] Azpiazu N. Lawrence P. A. Vincent J. P. Frasch M., 1996 Segmentation and specification of the Drosophila mesoderm. Genes Dev. 10: 3183–3194. 10.1101/gad.10.24.3183 8985186

[bib14] Babcock D. T. Brock A. R. Fish G. S. Wang Y. Perrin L., 2008 Circulating blood cells function as a surveillance system for damaged tissue in Drosophila larvae. Proc. Natl. Acad. Sci. USA 105: 10017–10022. 10.1073/pnas.0709951105 18632567 PMC2474562

[bib15] Bajgar A. Dolezal T., 2018 Extracellular adenosine modulates host-pathogen interactions through regulation of systemic metabolism during immune response in Drosophila. PLoS Pathog. 14: e1007022 10.1371/journal.ppat.1007022 29702691 PMC5942856

[bib16] Bajgar A. Kucerova K. Jonatova L. Tomcala A. Schneedorferova I., 2015 Extracellular adenosine mediates a systemic metabolic switch during immune response. PLoS Biol. 13: e1002135 10.1371/journal.pbio.1002135 25915062 PMC4411001

[bib17] Baldeosingh R. Gao H. Wu X. Fossett N., 2018 Hedgehog signaling from the posterior signaling center maintains U-shaped expression and a prohemocyte population in Drosophila. Dev. Biol. 441: 132–145. 10.1016/j.ydbio.2018.06.020 29966604 PMC6064674

[bib18] Basset A. Khush R. S. Braun A. Gardan L. Boccard F., 2000 The phytopathogenic bacteria Erwinia carotovora infects Drosophila and activates an immune response. Proc. Natl. Acad. Sci. USA 97: 3376–3381. 10.1073/pnas.97.7.3376 10725405 PMC16247

[bib19] Bataille L. Auge B. Ferjoux G. Haenlin M. Waltzer L., 2005 Resolving embryonic blood cell fate choice in Drosophila: interplay of GCM and RUNX factors. Development 132: 4635–4644. 10.1242/dev.02034 16176949

[bib20] Bate M. Rushton E., 1993 Myogenesis and muscle patterning in Drosophila. C. R. Acad. Sci. III 316: 1047–1061. 8076205

[bib21] Beiman M. Shilo B. Z. Volk T., 1996 Heartless, a Drosophila FGF receptor homolog, is essential for cell migration and establishment of several mesodermal lineages. Genes Dev. 10: 2993–3002. 10.1101/gad.10.23.2993 8957000

[bib22] Belenkaya T. Y. Han C. Yan D. Opoka R. J. Khodoun M., 2004 Drosophila Dpp morphogen movement is independent of dynamin-mediated endocytosis but regulated by the glypican members of heparan sulfate proteoglycans. Cell 119: 231–244. 10.1016/j.cell.2004.09.031 15479640

[bib23] Benmimoun B. Polesello C. Waltzer L. Haenlin M., 2012 Dual role for Insulin/TOR signaling in the control of hematopoietic progenitor maintenance in Drosophila. Development 139: 1713–1717. 10.1242/dev.080259 22510984

[bib24] Benmimoun B. Polesello C. Haenlin M. Waltzer L., 2015 The EBF transcription factor Collier directly promotes Drosophila blood cell progenitor maintenance independently of the niche. Proc. Natl. Acad. Sci. USA 112: 9052–9057. 10.1073/pnas.1423967112 26150488 PMC4517242

[bib25] Bergmann A. Agapite J. McCall K. Steller H., 1998 The Drosophila gene hid is a direct molecular target of Ras-dependent survival signaling. Cell 95: 331–341. 10.1016/S0092-8674(00)81765-1 9814704

[bib26] Bernardoni R. Vivancos V. Giangrande A., 1997 Glide/gcm is expressed and required in the scavenger cell lineage. Dev. Biol. 191: 118–130. 10.1006/dbio.1997.8702 9356176

[bib27] Bidla G. Lindgren M. Theopold U. Dushay M. S., 2005 Hemolymph coagulation and phenoloxidase in Drosophila larvae. Dev. Comp. Immunol. 29: 669–679. 10.1016/j.dci.2004.11.007 15854679

[bib28] Bidla G. Dushay M. S. Theopold U., 2007 Crystal cell rupture after injury in Drosophila requires the JNK pathway, small GTPases and the TNF homolog Eiger. J. Cell Sci. 120: 1209–1215. 10.1242/jcs.03420 17356067

[bib29] Binggeli O. Neyen C. Poidevin M. Lemaitre B., 2014 Prophenoloxidase activation is required for survival to microbial infections in Drosophila. PLoS Pathog. 10: e1004067 10.1371/journal.ppat.1004067 24788090 PMC4006879

[bib30] Bitra K. Suderman R. J. Strand M. R., 2012 Polydnavirus Ank proteins bind NF-kappaB homodimers and inhibit processing of Relish. PLoS Pathog. 8: e1002722 10.1371/journal.ppat.1002722 22654665 PMC3359993

[bib31] Bodmer R., 1993 The gene tinman is required for specification of the heart and visceral muscles in Drosophila. Development 118: 719–729. 7915669 10.1242/dev.118.3.719

[bib32] Bodmer R. Jan L. Y. Jan Y. N., 1990 A new homeobox-containing gene, msh-2, is transiently expressed early during mesoderm formation of Drosophila. Development 110: 661–669. 1982429 10.1242/dev.110.3.661

[bib33] Borkowski O. M. Brown N. H. Bate M., 1995 Anterior-posterior subdivision and the diversification of the mesoderm in Drosophila. Development 121: 4183–4193. 8575318 10.1242/dev.121.12.4183

[bib34] Brandt S. M. Dionne M. S. Khush R. S. Pham L. N. Vigdal T. J., 2004 Secreted bacterial effectors and host-produced Eiger/TNF drive death in a Salmonella-infected fruit fly. PLoS Biol. 2: e418 10.1371/journal.pbio.0020418 15562316 PMC532388

[bib35] Braun A. Lemaitre B. Lanot R. Zachary D. Meister M., 1997 Drosophila immunity: analysis of larval hemocytes by P-element-mediated enhancer trap. Genetics 147: 623–634. 9335599 10.1093/genetics/147.2.623 PMC1208184

[bib36] Braun A. Hoffmann J. A. Meister M., 1998 Analysis of the Drosophila host defense in domino mutant larvae, which are devoid of hemocytes. Proc. Natl. Acad. Sci. USA 95: 14337–14342. 10.1073/pnas.95.24.14337 9826701 PMC24374

[bib37] Brennan C. A. Anderson K. V., 2004 Drosophila: the genetics of innate immune recognition and response. Annu. Rev. Immunol. 22: 457–483. 10.1146/annurev.immunol.22.012703.104626 15032585

[bib38] Brennan C. A. Delaney J. R. Schneider D. S. Anderson K. V., 2007 Psidin is required in Drosophila blood cells for both phagocytic degradation and immune activation of the fat body. Curr. Biol. 17: 67–72. 10.1016/j.cub.2006.11.026 17208189

[bib39] Brennan J. J. Messerschmidt J. L. Williams L. M. Matthews B. J. Reynoso M., 2017 Sea anemone model has a single Toll-like receptor that can function in pathogen detection, NF-κB signal transduction, and development. Proc. Natl. Acad. Sci. USA 114: E10122–E10131. 10.1073/pnas.1711530114 29109290 PMC5703304

[bib40] Bretscher A. J. Honti V. Binggeli O. Burri O. Poidevin M., 2015 The Nimrod transmembrane receptor Eater is required for hemocyte attachment to the sessile compartment in Drosophila melanogaster. Biol. Open 4: 355–363. 10.1242/bio.201410595 25681394 PMC4359741

[bib41] Brückner K. Kockel L. Duchek P. Luque C. M. Rorth P., 2004 The PDGF/VEGF receptor controls blood cell survival in Drosophila. Dev. Cell 7: 73–84. 10.1016/j.devcel.2004.06.007 15239955

[bib42] Bunt S. Hooley C. Hu N. Scahill C. Weavers H., 2010 Hemocyte-secreted type IV collagen enhances BMP signaling to guide renal tubule morphogenesis in Drosophila. Dev. Cell 19: 296–306. 10.1016/j.devcel.2010.07.019 20708591 PMC2941037

[bib43] Cantor A. B. Orkin S. H., 2005 Coregulation of GATA factors by the Friend of GATA (FOG) family of multitype zinc finger proteins. Semin. Cell Dev. Biol. 16: 117–128. 10.1016/j.semcdb.2004.10.006 15659346

[bib44] Carton Y. Nappi A., 1991 The Drosophila immune-reaction and the parasitoid capacity to evade it - genetic and coevolutionary aspects. Acta Oecologica-Int. J. Ecol. 12: 89–104.

[bib45] Carton, Y., M. Bouletreau, J. Alphen, and J. van Lenteren, 1986 The *Drosophila* parasitic wasps, pp. 348–394 in *The Genetics and Biology of Drosophila*, Vol. III, edited by M. Ashburner, H. L. Carson, and J. N. Thompson. Academic Press, London.

[bib46] Castillejo-López C. Häcker U., 2005 The serine protease Sp7 is expressed in blood cells and regulates the melanization reaction in Drosophila. Biochem. Biophys. Res. Commun. 338: 1075–1082. 10.1016/j.bbrc.2005.10.042 16256951

[bib47] Cattenoz P. B. Popkova A. Southall T. D. Aiello G. Brand A. H., 2016 Functional conservation of the Glide/Gcm regulatory network controlling glia, hemocyte, and tendon cell differentiation in Drosophila. Genetics 202: 191–219. 10.1534/genetics.115.182154 26567182 PMC4701085

[bib48] Cerenius L. Lee B. L. Soderhall K., 2008 The proPO-system: pros and cons for its role in invertebrate immunity. Trends Immunol. 29: 263–271. 10.1016/j.it.2008.02.009 18457993

[bib49] Chakrabarti S. Dudzic J. P. Li X. Collas E. J. Boquete J. P., 2016 Remote control of intestinal stem cell activity by haemocytes in Drosophila. PLoS Genet. 12: e1006089 10.1371/journal.pgen.1006089 27231872 PMC4883764

[bib50] Charroux B. Royet J., 2009 Elimination of plasmatocytes by targeted apoptosis reveals their role in multiple aspects of the Drosophila immune response. Proc. Natl. Acad. Sci. USA 106: 9797–9802. 10.1073/pnas.0903971106 19482944 PMC2700997

[bib51] Chatterjee M. Ip Y. T., 2009 Pathogenic stimulation of intestinal stem cell response in Drosophila. J. Cell. Physiol. 220: 664–671. 10.1002/jcp.21808 19452446 PMC4003914

[bib52] Chen M. J. Yokomizo T. Zeigler B. M. Dzierzak E. Speck N. A., 2009 Runx1 is required for the endothelial to haematopoietic cell transition but not thereafter. Nature 457: 887–891. 10.1038/nature07619 19129762 PMC2744041

[bib53] Cho B. Spratford C. M. Yoon S. Cha N. Banerjee U., 2018 Systemic control of immune cell development by integrated carbon dioxide and hypoxia chemosensation in Drosophila. Nat. Commun. 9: 2679 10.1038/s41467-018-04990-3 29992947 PMC6041325

[bib54] Cho N. K. Keyes L. Johnson E. Heller J. Ryner L., 2002 Developmental control of blood cell migration by the Drosophila VEGF pathway. Cell 108: 865–876. 10.1016/S0092-8674(02)00676-1 11955438

[bib55] Chung Y. S. Kocks C., 2011 Recognition of pathogenic microbes by the Drosophila phagocytic pattern recognition receptor Eater. J. Biol. Chem. 286: 26524–26532. 10.1074/jbc.M110.214007 21613218 PMC3143617

[bib56] Colinet D. Schmitz A. Depoix D. Crochard D. Poirie M., 2007 Convergent use of RhoGAP toxins by eukaryotic parasites and bacterial pathogens. PLoS Pathog. 3: e203 10.1371/journal.ppat.0030203 18166080 PMC2156102

[bib57] Colinet D. Dubuffet A. Cazes D. Moreau S. Drezen J. M., 2009 A serpin from the parasitoid wasp Leptopilina boulardi targets the Drosophila phenoloxidase cascade. Dev. Comp. Immunol. 33: 681–689. 10.1016/j.dci.2008.11.013 19109990

[bib58] Colinet D. Cazes D. Belghazi M. Gatti J. L. Poirie M., 2011 Extracellular superoxide dismutase in insects: characterization, function, and interspecific variation in parasitoid wasp venom. J. Biol. Chem. 286: 40110–40121. 10.1074/jbc.M111.288845 21937434 PMC3220525

[bib59] Colinet D. Deleury E. Anselme C. Cazes D. Poulain J., 2013 Extensive inter- and intraspecific venom variation in closely related parasites targeting the same host: the case of Leptopilina parasitoids of Drosophila. Insect Biochem. Mol. Biol. 43: 601–611. 10.1016/j.ibmb.2013.03.010 23557852

[bib60] Cooper E. L., 1976 Evolution of blood cells. Ann. Immunol. (Paris) 127: 817–825. 1008528

[bib61] Cordero J. B. Macagno J. P. Stefanatos R. K. Strathdee K. E. Cagan R. L., 2010 Oncogenic Ras diverts a host TNF tumor suppressor activity into tumor promoter. Dev. Cell 18: 999–1011. 10.1016/j.devcel.2010.05.014 20627081 PMC3175220

[bib62] Cray C. Zaias J. Altman N. H., 2009 Acute phase response in animals: a review. Comp. Med. 59: 517–526. 20034426 PMC2798837

[bib63] Cripps R. M. Black B. L. Zhao B. Lien C. L. Schulz R. A., 1998 The myogenic regulatory gene Mef2 is a direct target for transcriptional activation by Twist during Drosophila myogenesis. Genes Dev. 12: 422–434. 10.1101/gad.12.3.422 9450935 PMC316486

[bib64] Crozatier M. Ubeda J. M. Vincent A. Meister M., 2004 Cellular immune response to parasitization in Drosophila requires the EBF orthologue collier. PLoS Biol. 2: E196 10.1371/journal.pbio.0020196 15314643 PMC509289

[bib65] Cuttell L. Vaughan A. Silva E. Escaron C. J. Lavine M., 2008 Undertaker, a Drosophila Junctophilin, links Draper-mediated phagocytosis and calcium homeostasis. Cell 135: 524–534. 10.1016/j.cell.2008.08.033 18984163

[bib66] D’Argenio D. A. Gallagher L. A. Berg C. A. Manoil C., 2001 Drosophila as a model host for Pseudomonas aeruginosa infection. J. Bacteriol. 183: 1466–1471. 10.1128/JB.183.4.1466-1471.2001 11157963 PMC95024

[bib67] Defaye A. Evans I. Crozatier M. Wood W. Lemaitre B., 2009 Genetic ablation of Drosophila phagocytes reveals their contribution to both development and resistance to bacterial infection. J. Innate Immun. 1: 322–334. 10.1159/000210264 20375589

[bib68] De Gregorio E. Han S. J. Lee W. J. Baek M. J. Osaki T., 2002 An immune-responsive Serpin regulates the melanization cascade in Drosophila. Dev. Cell 3: 581–592. 10.1016/S1534-5807(02)00267-8 12408809

[bib69] De Sutter D. Buscema M., 1977 Isolation of a highly pure archeocyte fraction from the fresh-water sponge Ephydatia fluviatilis. Wilehm Roux Arch Dev Biol 183: 149–153. 10.1007/BF00848784 28304902

[bib70] De Sutter D. Van de Vyver G., 1977 Aggregative properties of different cell types of the fresh-water sponge Ephydatia fluviatilis isolated on ficoll gradients. Wilehm Roux Arch Dev Biol 181: 151–161. 10.1007/BF00848439 28304912

[bib71] Dey N. S. Ramesh P. Chugh M. Mandal S. Mandal L., 2016 Dpp dependent Hematopoietic stem cells give rise to Hh dependent blood progenitors in larval lymph gland of Drosophila. Elife 5: e18295 10.7554/eLife.18295 27782877 PMC5120881

[bib72] Dijkers P. F. O’Farrell P. H., 2007 Drosophila calcineurin promotes induction of innate immune responses. Curr. Biol. 17: 2087–2093. 10.1016/j.cub.2007.11.001 18060786 PMC2180389

[bib73] Dionne M. S. Ghori N. Schneider D. S., 2003 Drosophila melanogaster is a genetically tractable model host for Mycobacterium marinum. Infect. Immun. 71: 3540–3550. 10.1128/IAI.71.6.3540-3550.2003 12761139 PMC155752

[bib74] Dolezal T. Dolezelova E. Zurovec M. Bryant P. J., 2005 A role for adenosine deaminase in Drosophila larval development. PLoS Biol. 3: e201 10.1371/journal.pbio.0030201 15907156 PMC1135298

[bib75] Dostálová A. Rommelaere S. Poidevin M. Lemaitre B., 2017 Thioester-containing proteins regulate the Toll pathway and play a role in Drosophila defence against microbial pathogens and parasitoid wasps. BMC Biol. 15: 79 10.1186/s12915-017-0408-0 28874153 PMC5584532

[bib76] Doumpas N. Jekely G. Teleman A. A., 2013 Wnt6 is required for maxillary palp formation in Drosophila. BMC Biol. 11: 104 [corrigends: BMC Biol. 13: 100 (2015)]. 10.1186/1741-7007-11-104 24090348 PMC3854539

[bib77] Dragojlovic-Munther M. Martinez-Agosto J. A., 2012 Multifaceted roles of PTEN and TSC orchestrate growth and differentiation of Drosophila blood progenitors. Development 139: 3752–3763. 10.1242/dev.074203 22951642 PMC3445307

[bib78] Dragojlovic-Munther M. Martinez-Agosto J. A., 2013 Extracellular matrix-modulated heartless signaling in Drosophila blood progenitors regulates their differentiation via a Ras/ETS/FOG pathway and target of rapamycin function. Dev. Biol. 384: 313–330. 10.1016/j.ydbio.2013.04.004 23603494 PMC4256155

[bib79] Dudzic J. P. Kondo S. Ueda R. Bergman C. M. Lemaitre B., 2015 Drosophila innate immunity: regional and functional specialization of prophenoloxidases. BMC Biol. 13: 81 10.1186/s12915-015-0193-6 26437768 PMC4595066

[bib80] Duvic B. Hoffmann J. A. Meister M. Royet J., 2002 Notch signaling controls lineage specification during Drosophila larval hematopoiesis. Curr. Biol. 12: 1923–1927. 10.1016/S0960-9822(02)01297-6 12445385

[bib81] Dzierzak E. Speck N. A., 2008 Of lineage and legacy: the development of mammalian hematopoietic stem cells. Nat. Immunol. 9: 129–136. 10.1038/ni1560 18204427 PMC2696344

[bib82] Eastham L. E. S., 1930 The formation of germ layers in insects. Biol. Rev. Camb. Philos. Soc. 5: 1–29. 10.1111/j.1469-185X.1930.tb00891.x

[bib83] Elrod-Erickson M. Mishra S. Schneider D., 2000 Interactions between the cellular and humoral immune responses in Drosophila. Curr. Biol. 10: 781–784. 10.1016/S0960-9822(00)00569-8 10898983

[bib84] Ema M. Rossant J., 2003 Cell fate decisions in early blood vessel formation. Trends Cardiovasc. Med. 13: 254–259. 10.1016/S1050-1738(03)00105-1 12922023

[bib85] Ernst O. P. Lodowski D. T. Elstner M. Hegemann P. Brown L. S., 2014 Microbial and animal rhodopsins: structures, functions, and molecular mechanisms. Chem. Rev. 114: 126–163. 10.1021/cr4003769 24364740 PMC3979449

[bib86] Evans C. J. Hartenstein V. Banerjee U., 2003 Thicker than blood: conserved mechanisms in Drosophila and vertebrate hematopoiesis. Dev. Cell 5: 673–690. 10.1016/S1534-5807(03)00335-6 14602069

[bib87] Evans C. J. Olson J. M. Ngo K. T. Kim E. Lee N. E., 2009 G-TRACE: rapid Gal4-based cell lineage analysis in Drosophila. Nat. Methods 6: 603–605. 10.1038/nmeth.1356 19633663 PMC2754220

[bib88] Evans C. J. Liu T. Banerjee U., 2014 Drosophila hematopoiesis: markers and methods for molecular genetic analysis. Methods 68: 242–251. 10.1016/j.ymeth.2014.02.038 24613936 PMC4051208

[bib89] Evans I. R. Rodrigues F. S. Armitage E. L. Wood W., 2015 Draper/CED-1 mediates an ancient damage response to control inflammatory blood cell migration in vivo. Curr. Biol. 25: 1606–1612. 10.1016/j.cub.2015.04.037 26028435 PMC4503800

[bib90] Evgenov O. V. Pacher P. Schmidt P. M. Hasko G. Schmidt H. H., 2006 NO-independent stimulators and activators of soluble guanylate cyclase: discovery and therapeutic potential. Nat. Rev. Drug Discov. 5: 755–768. 10.1038/nrd2038 16955067 PMC2225477

[bib91] Feinberg E. H. Vanhoven M. K. Bendesky A. Wang G. Fetter R. D., 2008 GFP reconstitution across synaptic partners (GRASP) defines cell contacts and synapses in living nervous systems. Neuron 57: 353–363. 10.1016/j.neuron.2007.11.030 18255029

[bib92] Ferguson G. B. Martinez-Agosto J. A., 2014 Yorkie and Scalloped signaling regulates Notch-dependent lineage specification during Drosophila hematopoiesis. Curr. Biol. 24: 2665–2672. 10.1016/j.cub.2014.09.081 25454586 PMC4256154

[bib93] Ferguson G. B. Martinez-Agosto J. A., 2017 The TEAD family transcription factor Scalloped regulates blood progenitor maintenance and proliferation in Drosophila through PDGF/VEGFR receptor (Pvr) signaling. Dev. Biol. 425: 21–32. 10.1016/j.ydbio.2017.03.016 28322737

[bib94] Ferjoux G. Auge B. Boyer K. Haenlin M. Waltzer L., 2007 A GATA/RUNX cis-regulatory module couples Drosophila blood cell commitment and differentiation into crystal cells. Dev. Biol. 305: 726–734. 10.1016/j.ydbio.2007.03.010 17418114

[bib95] Fessler J. H. Fessler L. I., 1989 Drosophila extracellular matrix. Annu. Rev. Cell Biol. 5: 309–339. 10.1146/annurev.cb.05.110189.001521 2557060

[bib96] Flaherty M. S. Salis P. Evans C. J. Ekas L. A. Marouf A., 2010 chinmo is a functional effector of the JAK/STAT pathway that regulates eye development, tumor formation, and stem cell self-renewal in Drosophila. Dev. Cell 18: 556–568. 10.1016/j.devcel.2010.02.006 20412771 PMC2859208

[bib97] Fogarty C. E. Diwanji N. Lindblad J. L. Tare M. Amcheslavsky A., 2016 Extracellular reactive oxygen species drive apoptosis-induced proliferation via Drosophila macrophages. Curr. Biol. 26: 575–584. 10.1016/j.cub.2015.12.064 26898463 PMC4765900

[bib98] Fogerty F. J. Fessler L. I. Bunch T. A. Yaron Y. Parker C. G., 1994 Tiggrin, a novel Drosophila extracellular matrix protein that functions as a ligand for Drosophila alpha PS2 beta PS integrins. Development 120: 1747–1758. 7924982 10.1242/dev.120.7.1747

[bib99] Foley E. O’Farrell P. H., 2003 Nitric oxide contributes to induction of innate immune responses to gram-negative bacteria in Drosophila. Genes Dev. 17: 115–125. 10.1101/gad.1018503 12514104 PMC195964

[bib100] Fossett N. Tevosian S. G. Gajewski K. Zhang Q. Orkin S. H., 2001 The Friend of GATA proteins U-shaped, FOG-1, and FOG-2 function as negative regulators of blood, heart, and eye development in Drosophila. Proc. Natl. Acad. Sci. USA 98: 7342–7347. 10.1073/pnas.131215798 11404479 PMC34670

[bib101] Fossett N. Hyman K. Gajewski K. Orkin S. H. Schulz R. A., 2003 Combinatorial interactions of serpent, lozenge, and U-shaped regulate crystal cell lineage commitment during Drosophila hematopoiesis. Proc. Natl. Acad. Sci. USA 100: 11451–11456. 10.1073/pnas.1635050100 14504400 PMC208778

[bib102] Franc N. C. Dimarcq J. L. Lagueux M. Hoffmann J. Ezekowitz R. A., 1996 Croquemort, a novel Drosophila hemocyte/macrophage receptor that recognizes apoptotic cells. Immunity 4: 431–443. 10.1016/S1074-7613(00)80410-0 8630729

[bib103] Frasch M., 1995 Induction of visceral and cardiac mesoderm by ectodermal Dpp in the early Drosophila embryo. Nature 374: 464–467. 10.1038/374464a0 7700357

[bib104] Freeman M. R. Delrow J. Kim J. Johnson E. Doe C. Q., 2003 Unwrapping glial biology: Gcm target genes regulating glial development, diversification, and function. Neuron 38: 567–580. 10.1016/S0896-6273(03)00289-7 12765609

[bib105] Fujiwara Y. Chang A. N. Williams A. M. Orkin S. H., 2004 Functional overlap of GATA-1 and GATA-2 in primitive hematopoietic development. Blood 103: 583–585. 10.1182/blood-2003-08-2870 14504093

[bib106] Fuller M. T. Spradling A. C., 2007 Male and female Drosophila germline stem cells: two versions of immortality. Science 316: 402–404. 10.1126/science.1140861 17446390

[bib107] Gajewski K. M. Sorrentino R. P. Lee J. H. Zhang Q. Russell M., 2007 Identification of a crystal cell-specific enhancer of the black cells prophenoloxidase gene in Drosophila. Genesis 45: 200–207. 10.1002/dvg.20285 17417793

[bib108] Galko M. J. Krasnow M. A., 2004 Cellular and genetic analysis of wound healing in Drosophila larvae. PLoS Biol. 2: E239 10.1371/journal.pbio.0020239 15269788 PMC479041

[bib109] Ganesan S. Aggarwal K. Paquette N. Silverman N., 2011 NF-kappaB/Rel proteins and the humoral immune responses of Drosophila melanogaster. Curr. Top. Microbiol. Immunol. 349: 25–60. 10.1007/82_2010_107 20852987 PMC3083852

[bib110] Gao H. Wu X. Fossett N., 2009 Upregulation of the Drosophila friend of GATA gene U-shaped by JAK/STAT signaling maintains lymph gland prohemocyte potency. Mol. Cell. Biol. 29: 6086–6096. 10.1128/MCB.00244-09 19737914 PMC2772570

[bib111] Gao H. Wu X. Fossett N., 2013 Drosophila E-cadherin functions in hematopoietic progenitors to maintain multipotency and block differentiation. PLoS One 8: e74684 10.1371/journal.pone.0074684 24040319 PMC3764055

[bib112] Gao H. Wu X. Simon L. Fossett N., 2014 Antioxidants maintain E-cadherin levels to limit Drosophila prohemocyte differentiation. PLoS One 9: e107768 10.1371/journal.pone.0107768 25226030 PMC4167200

[bib113] Gateff E., 1994 Tumor-suppressor and overgrowth suppressor genes of Drosophila-Melanogaster - developmental aspects. Int. J. Dev. Biol. 38: 565–590. 7779680

[bib114] Gehring W. J., 1996 The master control gene for morphogenesis and evolution of the eye. Genes Cells 1: 11–15. 10.1046/j.1365-2443.1996.11011.x 9078363

[bib115] Gerttula S. Jin Y. S. Anderson K. V., 1988 Zygotic expression and activity of the Drosophila Toll gene, a gene required maternally for embryonic dorsal-ventral pattern formation. Genetics 119: 123–133. 2456252 10.1093/genetics/119.1.123 PMC1203330

[bib116] Ghosh S. Singh A. Mandal S. Mandal L., 2015 Active hematopoietic hubs in Drosophila adults generate hemocytes and contribute to immune response. Dev. Cell 33: 478–488. 10.1016/j.devcel.2015.03.014 25959225 PMC4448147

[bib379] Gold K. S. Brückner K., 2015 Macrophages and cellular immunity in Drosophila melanogaster. Semin. Immunol 6: 357–368. doi.org/10.1016/j.smim.2016.03.010 PMC5012540 27117654

[bib117] Goto A. Kadowaki T. Kitagawa Y., 2003 Drosophila hemolectin gene is expressed in embryonic and larval hemocytes and its knock down causes bleeding defects. Dev. Biol. 264: 582–591. 10.1016/j.ydbio.2003.06.001 14651939

[bib118] Gramates L. S. Marygold S. J. Santos G. D. Urbano J. M. Antonazzo G., 2017 FlyBase at 25: looking to the future. Nucleic Acids Res. 45: D663–D671. 10.1093/nar/gkw1016 27799470 PMC5210523

[bib119] Grigorian M. Hartenstein V., 2013 Hematopoiesis and hematopoietic organs in arthropods. Dev. Genes Evol. 223: 103–115. 10.1007/s00427-012-0428-2 23319182 PMC3873168

[bib120] Grigorian M. Mandal L. Hakimi M. Ortiz I. Hartenstein V., 2011a The convergence of Notch and MAPK signaling specifies the blood progenitor fate in the Drosophila mesoderm. Dev. Biol. 353: 105–118. 10.1016/j.ydbio.2011.02.024 21382367 PMC3312814

[bib121] Grigorian M. Mandal L. Hartenstein V., 2011b Hematopoiesis at the onset of metamorphosis: terminal differentiation and dissociation of the Drosophila lymph gland. Dev. Genes Evol. 221: 121–131. 10.1007/s00427-011-0364-6 21509534 PMC4278756

[bib122] Grigorian M. Liu T. Banerjee U. Hartenstein V., 2013 The proteoglycan Trol controls the architecture of the extracellular matrix and balances proliferation and differentiation of blood progenitors in the Drosophila lymph gland. Dev. Biol. 384: 301–312. 10.1016/j.ydbio.2013.03.007 23510717 PMC4278754

[bib123] Gueguen G. Kalamarz M. E. Ramroop J. Uribe J. Govind S., 2013 Polydnaviral ankyrin proteins aid parasitic wasp survival by coordinate and selective inhibition of hematopoietic and immune NF-kappa B signaling in insect hosts. PLoS Pathog. 9: e1003580 10.1371/journal.ppat.1003580 24009508 PMC3757122

[bib124] Guillou A. Troha K. Wang H. Franc N. C. Buchon N., 2016 The Drosophila CD36 homologue croquemort is required to maintain immune and Gut homeostasis during development and aging. PLoS Pathog. 12: e1005961 10.1371/journal.ppat.1005961 27780230 PMC5079587

[bib125] Han Z. Olson E. N., 2005 Hand is a direct target of Tinman and GATA factors during Drosophila cardiogenesis and hematopoiesis. Development 132: 3525–3536. 10.1242/dev.01899 15975941

[bib126] Han Z. Yi P. Li X. Olson E. N., 2006 Hand, an evolutionarily conserved bHLH transcription factor required for Drosophila cardiogenesis and hematopoiesis. Development 133: 1175–1182. 10.1242/dev.02285 16467358

[bib127] Hanoun M. Maryanovich M. Arnal-Estape A. Frenette P. S., 2015 Neural regulation of hematopoiesis, inflammation, and cancer. Neuron 86: 360–373. 10.1016/j.neuron.2015.01.026 25905810 PMC4416657

[bib128] Hanratty W. P. Dearolf C. R., 1993 The Drosophila tumorous-lethal hematopoietic oncogene is a dominant mutation in the hopscotch locus. Mol. Gen. Genet. 238: 33–37. 8479437 10.1007/BF00279527

[bib129] Hao Y. Jin L. H., 2017 Dual role for Jumu in the control of hematopoietic progenitors in the Drosophila lymph gland. Elife 6: e25094 10.7554/eLife.25094 28350299 PMC5391210

[bib130] Harrison D. A. Binari R. Nahreini T. S. Gilman M. Perrimon N., 1995 Activation of a Drosophila Janus kinase (JAK) causes hematopoietic neoplasia and developmental defects. EMBO J. 14: 2857–2865. 10.1002/j.1460-2075.1995.tb07285.x 7796812 PMC398404

[bib131] Hartenstein V., 2006 Blood cells and blood cell development in the animal kingdom. Annu. Rev. Cell Dev. Biol. 22: 677–712. 10.1146/annurev.cellbio.22.010605.093317 16824014

[bib132] Hartenstein V. Chipman A. D., 2015 Hexapoda: a Drosophila’s view of development, pp. 1–91 in Evolutionary Developmental Biology of Invertebrates 5: Ecdysozoa III: Hexapoda, edited by Wanninger. A. Springer Verlag, Vienna 10.1007/978-3-7091-1868-9_1

[bib133] Hartenstein V. Mandal L., 2006 The blood/vascular system in a phylogenetic perspective. BioEssays 28: 1203–1210. 10.1002/bies.20497 17120194

[bib134] Hauling T. Krautz R. Markus R. Volkenhoff A. Kucerova L., 2014 A Drosophila immune response against Ras-induced overgrowth. Biol. Open 3: 250–260. 10.1242/bio.20146494 24659248 PMC3988794

[bib135] Heino T. I. Karpanen T. Wahlstrom G. Pulkkinen M. Eriksson U., 2001 The Drosophila VEGF receptor homolog is expressed in hemocytes. Mech. Dev. 109: 69–77. 10.1016/S0925-4773(01)00510-X 11677054

[bib136] Hoffmann J. A. Reichhart J. M., 2002 Drosophila innate immunity: an evolutionary perspective. Nat. Immunol. 3: 121–126. 10.1038/ni0202-121 11812988

[bib137] Holz A. Bossinger B. Strasser T. Janning W. Klapper R., 2003 The two origins of hemocytes in Drosophila. Development 130: 4955–4962. 10.1242/dev.00702 12930778

[bib138] Hombría J. C. Brown S. Hader S. Zeidler M. P., 2005 Characterisation of Upd2, a Drosophila JAK/STAT pathway ligand. Dev. Biol. 288: 420–433. 10.1016/j.ydbio.2005.09.040 16277982

[bib139] Honti V. Kurucz E. Csordas G. Laurinyecz B. Markus R., 2009 In vivo detection of lamellocytes in Drosophila melanogaster. Immunol. Lett. 126: 83–84. 10.1016/j.imlet.2009.08.004 19695290

[bib140] Honti V. Csordas G. Markus R. Kurucz E. Jankovics F., 2010 Cell lineage tracing reveals the plasticity of the hemocyte lineages and of the hematopoietic compartments in Drosophila melanogaster. Mol. Immunol. 47: 1997–2004. 10.1016/j.molimm.2010.04.017 20483458

[bib141] Honti V. Cinege G. Csordas G. Kurucz E. Zsamboki J., 2013 Variation of NimC1 expression in Drosophila stocks and transgenic strains. Fly (Austin) 7: 263–266. 10.4161/fly.25654 23899817 PMC3896499

[bib142] Honti V. Csordas G. Kurucz E. Markus R. Ando I., 2014 The cell-mediated immunity of Drosophila melanogaster: hemocyte lineages, immune compartments, microanatomy and regulation. Dev. Comp. Immunol. 42: 47–56. 10.1016/j.dci.2013.06.005 23800719

[bib143] Inamdar M. S., 2002 Stem cell identity: life is plastic, it’s fantastic! J. Biosci. 27: 93–95. 10.1007/BF02703764 11937678

[bib144] Irving P. Ubeda J. M. Doucet D. Troxler L. Lagueux M., 2005 New insights into Drosophila larval haemocyte functions through genome-wide analysis. Cell. Microbiol. 7: 335–350. 10.1111/j.1462-5822.2004.00462.x 15679837

[bib145] Jiang H. Edgar B. A., 2009 EGFR signaling regulates the proliferation of Drosophila adult midgut progenitors. Development 136: 483–493. 10.1242/dev.026955 19141677 PMC2687592

[bib146] Jin L. H. Shim J. Yoon J. S. Kim B. Kim J., 2008 Identification and functional analysis of antifungal immune response genes in Drosophila. PLoS Pathog. 4: e1000168 10.1371/journal.ppat.1000168 18833296 PMC2542415

[bib147] Jones J. C., 1970 Hemocytopoiesis in insects, pp. 7–65 in Regulation of Hematopoiesis, Vol. 1, edited by Gordon A. S. Appleton-Century-Crofts, New York.

[bib148] Jones W. D. Cayirlioglu P. Kadow I. G. Vosshall L. B., 2007 Two chemosensory receptors together mediate carbon dioxide detection in Drosophila. Nature 445: 86–90. 10.1038/nature05466 17167414

[bib149] Jung S. H. Evans C. J. Uemura C. Banerjee U., 2005 The Drosophila lymph gland as a developmental model of hematopoiesis. Development 132: 2521–2533. 10.1242/dev.01837 15857916

[bib150] Kaaya G. P. Ratcliffe N. A., 1982 Comparative study of hemocytes and associated cells of some medically important dipterans. J. Morphol. 173: 351–365. 10.1002/jmor.1051730310 6764649

[bib151] Kalamarz M. E. Paddibhatla I. Nadar C. Govind S., 2012 Sumoylation is tumor-suppressive and confers proliferative quiescence to hematopoietic progenitors in Drosophila melanogaster larvae. Biol. Open 1: 161–172. 10.1242/bio.2011043 23213407 PMC3507282

[bib152] Kallio J. Myllymaki H. Gronholm J. Armstrong M. Vanha-aho L. M., 2010 Eye transformer is a negative regulator of Drosophila JAK/STAT signaling. FASEB J. 24: 4467–4479. 10.1096/fj.10-162784 20624926

[bib153] Karpac J. Younger A. Jasper H., 2011 Dynamic coordination of innate immune signaling and insulin signaling regulates systemic responses to localized DNA damage. Dev. Cell 20: 841–854. 10.1016/j.devcel.2011.05.011 21664581 PMC3151532

[bib154] Kelsey E. M. Luo X. Bruckner K. Jasper H., 2012 Schnurri regulates hemocyte function to promote tissue recovery after DNA damage. J. Cell Sci. 125: 1393–1400. 10.1242/jcs.095323 22275438 PMC3336376

[bib155] Kenmoku H. Hori A. Kuraishi T. Kurata S., 2017 A novel mode of induction of the humoral innate immune response in Drosophila larvae. Dis. Model. Mech. 10: 271–281. 10.1242/dmm.027102 28250052 PMC5374318

[bib156] Khadilkar R. J. Rodrigues D. Mote R. D. Sinha A. R. Kulkarni V., 2014 ARF1-GTP regulates Asrij to provide endocytic control of Drosophila blood cell homeostasis. Proc. Natl. Acad. Sci. USA 111: 4898–4903. 10.1073/pnas.1303559111 24707047 PMC3977295

[bib157] Khadilkar R. J. Vogl W. Goodwin K. Tanentzapf G., 2017 Modulation of occluding junctions alters the hematopoietic niche to trigger immune activation. Elife 6: e28081 10.7554/eLife.28081 28841136 PMC5597334

[bib158] Kim E. Goraksha-Hicks P. Li L. Neufeld T. P. Guan K. L., 2008 Regulation of TORC1 by Rag GTPases in nutrient response. Nat. Cell Biol. 10: 935–945. 10.1038/ncb1753 18604198 PMC2711503

[bib159] Kim M. J. Choe K. M., 2014 Basement membrane and cell integrity of self-tissues in maintaining Drosophila immunological tolerance. PLoS Genet. 10: e1004683 10.1371/journal.pgen.1004683 25329560 PMC4199487

[bib160] Kimbrell D. A. Hice C. Bolduc C. Kleinhesselink K. Beckingham K., 2002 The Dorothy enhancer has Tinman binding sites and drives hopscotch-induced tumor formation. Genesis 34: 23–28. 10.1002/gene.10134 12324942

[bib161] Klinedinst S. L. Bodmer R., 2003 Gata factor Pannier is required to establish competence for heart progenitor formation. Development 130: 3027–3038. 10.1242/dev.00517 12756184

[bib162] Kocks C. Cho J. H. Nehme N. Ulvila J. Pearson A. M., 2005 Eater, a transmembrane protein mediating phagocytosis of bacterial pathogens in Drosophila. Cell 123: 335–346. 10.1016/j.cell.2005.08.034 16239149

[bib163] Kroeger P. T. Jr Tokusumi T. Schulz R. A., 2012 Transcriptional regulation of eater gene expression in Drosophila blood cells. Genesis 50: 41–49. 10.1002/dvg.20787 21809435

[bib164] Krzemień J. Dubois L. Makki R. Meister M. Vincent A., 2007 Control of blood cell homeostasis in Drosophila larvae by the posterior signalling centre. Nature 446: 325–328. 10.1038/nature05650 17361184

[bib165] Krzemien J. Oyallon J. Crozatier M. Vincent A., 2010 Hematopoietic progenitors and hemocyte lineages in the Drosophila lymph gland. Dev. Biol. 346: 310–319. 10.1016/j.ydbio.2010.08.003 20707995

[bib166] Kulkarni V. Khadilkar R. J. Magadi S. S. Inamdar M. S., 2011 Asrij maintains the stem cell niche and controls differentiation during Drosophila lymph gland hematopoiesis. PLoS One 6: e27667 [corrigenda: PLoS One 7 (2012)]. 10.1371/journal.pone.0027667 22110713 PMC3215734

[bib167] Kurant E. Axelrod S. Leaman D. Gaul U., 2008 Six-microns-under acts upstream of Draper in the glial phagocytosis of apoptotic neurons. Cell 133: 498–509. 10.1016/j.cell.2008.02.052 18455990 PMC2730188

[bib168] Kurucz E. Zettervall C. J. Sinka R. Vilmos P. Pivarcsi A., 2003 Hemese, a hemocyte-specific transmembrane protein, affects the cellular immune response in Drosophila. Proc. Natl. Acad. Sci. USA 100: 2622–2627. 10.1073/pnas.0436940100 12598653 PMC151390

[bib169] Kurucz E. Markus R. Zsamboki J. Folkl-Medzihradszky K. Darula Z., 2007a Nimrod, a putative phagocytosis receptor with EGF repeats in Drosophila plasmatocytes. Curr. Biol. 17: 649–654. 10.1016/j.cub.2007.02.041 17363253

[bib170] Kurucz E. Vaczi B. Markus R. Laurinyecz B. Vilmos P., 2007b Definition of Drosophila hemocyte subsets by cell-type specific antigens. Acta Biol. Hung. 58: 95–111. 10.1556/ABiol.58.2007.Suppl.8 18297797

[bib171] Kusche-Gullberg M. Garrison K. MacKrell A. J. Fessler L. I. Fessler J. H., 1992 Laminin A chain: expression during Drosophila development and genomic sequence. EMBO J. 11: 4519–4527. 10.1002/j.1460-2075.1992.tb05553.x 1425586 PMC557027

[bib172] Kwon J. Y. Dahanukar A. Weiss L. A. Carlson J. R., 2007 The molecular basis of CO2 reception in Drosophila. Proc. Natl. Acad. Sci. USA 104: 3574–3578. 10.1073/pnas.0700079104 17360684 PMC1805529

[bib173] Labrosse C. Eslin P. Doury G. Drezen J. M. Poirie M., 2005a Haemocyte changes in D-Melanogaster in response to long gland components of the parasitoid wasp Leptopilina boulardi: a Rho-GAP protein as an important factor. J. Insect Physiol. 51: 161–170. 10.1016/j.jinsphys.2004.10.004 15749101

[bib174] Labrosse C. Staslak K. Lesobre J. Grangeia A. Huguet E., 2005b A RhoGAP protein as a main immune suppressive factor in the Leptopilina boulardi (Hymenoptera, Figitidae) - Drosophila melanogaster interaction. Insect Biochem. Mol. Biol. 35: 93–103. 10.1016/j.ibmb.2004.10.004 15681220

[bib175] Lagueux M. Perrodou E. Levashina E. A. Capovilla M. Hoffmann J. A., 2000 Constitutive expression of a complement-like protein in toll and JAK gain-of-function mutants of Drosophila. Proc. Natl. Acad. Sci. USA 97: 11427–11432. 10.1073/pnas.97.21.11427 11027343 PMC17216

[bib176] Lanot R. Zachary D. Holder F. Meister M., 2001 Postembryonic hematopoiesis in Drosophila. Dev. Biol. 230: 243–257. 10.1006/dbio.2000.0123 11161576

[bib177] Lavine M. D. Strand M. R., 2002 Insect hemocytes and their role in immunity. Insect Biochem. Mol. Biol. 32: 1295–1309. 10.1016/S0965-1748(02)00092-9 12225920

[bib178] Lebestky T. Chang T. Hartenstein V. Banerjee U., 2000 Specification of Drosophila hematopoietic lineage by conserved transcription factors. Science 288: 146–149. 10.1126/science.288.5463.146 10753120

[bib179] Lebestky T. Jung S. H. Banerjee U., 2003 A Serrate-expressing signaling center controls Drosophila hematopoiesis. Genes Dev. 17: 348–353. 10.1101/gad.1052803 12569125 PMC195988

[bib180] Leclerc V. Pelte N. El Chamy L. Martinelli C. Ligoxygakis P., 2006 Prophenoloxidase activation is not required for survival to microbial infections in Drosophila. EMBO Rep. 7: 231–235. 10.1038/sj.embor.7400592 16322759 PMC1369246

[bib181] Leitão A. B., and ÉSucena., 2015 Drosophila sessile hemocyte clusters are true hematopoietic tissues that regulate larval blood cell differentiation. Elife 4: e06166. 10.7554/eLife.06166 PMC4357286 25650737

[bib182] Lemaitre B. Hoffmann J., 2007 The host defense of Drosophila melanogaster. Annu. Rev. Immunol. 25: 697–743. 10.1146/annurev.immunol.25.022106.141615 17201680

[bib183] Lemaitre B. Meister M. Govind S. Georgel P. Steward R., 1995 Functional analysis and regulation of nuclear import of dorsal during the immune response in Drosophila. EMBO J. 14: 536–545. 10.1002/j.1460-2075.1995.tb07029.x 7859742 PMC398111

[bib184] Lemaitre B. Reichhart J. M. Hoffmann J. A., 1997 Drosophila host defense: differential induction of antimicrobial peptide genes after infection by various classes of microorganisms. Proc. Natl. Acad. Sci. USA 94: 14614–14619. 10.1073/pnas.94.26.14614 9405661 PMC25070

[bib185] Leptin M., 1991 Twist and snail as positive and negative regulators during Drosophila mesoderm development. Genes Dev. 5: 1568–1576. 10.1101/gad.5.9.1568 1884999

[bib186] Letourneau M. Lapraz F. Sharma A. Vanzo N. Waltzer L., 2016 Drosophila hematopoiesis under normal conditions and in response to immune stress. FEBS Lett. 590: 4034–4051. 10.1002/1873-3468.12327 27455465

[bib187] Lien C. L. Wu C. Mercer B. Webb R. Richardson J. A., 1999 Control of early cardiac-specific transcription of Nkx2–5 by a GATA-dependent enhancer. Development 126: 75–84. 9834187 10.1242/dev.126.1.75

[bib188] Ligoxygakis P. Pelte N. Ji C. Leclerc V. Duvic B., 2002 A serpin mutant links Toll activation to melanization in the host defence of Drosophila. EMBO J. 21: 6330–6337. 10.1093/emboj/cdf661 12456640 PMC136964

[bib189] Lilly B. Galewsky S. Firulli A. B. Schulz R. A. Olson E. N., 1994 D-MEF2: a MADS box transcription factor expressed in differentiating mesoderm and muscle cell lineages during Drosophila embryogenesis. Proc. Natl. Acad. Sci. USA 91: 5662–5666. 10.1073/pnas.91.12.5662 8202544 PMC44056

[bib190] Lindgren M. Riazi R. Lesch C. Wilhelmsson C. Theopold U., 2008 Fondue and transglutaminase in the Drosophila larval clot. J. Insect Physiol. 54: 586–592. 10.1016/j.jinsphys.2007.12.008 18222466

[bib191] Lindner J. R. Hillman P. R. Barrett A. L. Jackson M. C. Perry T. L., 2007 The Drosophila Perlecan gene trol regulates multiple signaling pathways in different developmental contexts. BMC Dev. Biol. 7: 121 10.1186/1471-213X-7-121 17980035 PMC2174950

[bib192] Lo P. C. Skeath J. B. Gajewski K. Schulz R. A. Frasch M., 2002 Homeotic genes autonomously specify the anteroposterior subdivision of the Drosophila dorsal vessel into aorta and heart. Dev. Biol. 251: 307–319. 10.1006/dbio.2002.0839 12435360

[bib193] Louradour I. Sharma A. Morin-Poulard I. Letourneau M. Vincent A., 2017 Reactive oxygen species-dependent Toll/NF-κB activation in the Drosophila hematopoietic niche confers resistance to wasp parasitism. Elife 6: e25496 10.7554/eLife.25496 29091025 PMC5681226

[bib194] Luo H. Hanratty W. P. Dearolf C. R., 1995 An amino acid substitution in the Drosophila hopTum-l Jak kinase causes leukemia-like hematopoietic defects. EMBO J. 14: 1412–1420. 10.1002/j.1460-2075.1995.tb07127.x 7729418 PMC398227

[bib195] Luo H. Rose P. Barber D. Hanratty W. P. Lee S., 1997 Mutation in the Jak kinase JH2 domain hyperactivates Drosophila and mammalian Jak-Stat pathways. Mol. Cell. Biol. 17: 1562–1571. 10.1128/MCB.17.3.1562 9032284 PMC231882

[bib196] Mace K. A. Pearson J. C. McGinnis W., 2005 An epidermal barrier wound repair pathway in Drosophila is mediated by grainy head. Science 308: 381–385. 10.1126/science.1107573 15831751

[bib197] Macpherson L. J. Zaharieva E. E. Kearney P. J. Alpert M. H. Lin T. Y., 2015 Dynamic labelling of neural connections in multiple colours by trans-synaptic fluorescence complementation. Nat. Commun. 6: 10024 10.1038/ncomms10024 26635273 PMC4686661

[bib198] Makhijani K. Alexander B. Tanaka T. Rulifson E. Bruckner K., 2011 The peripheral nervous system supports blood cell homing and survival in the Drosophila larva. Development 138: 5379–5391. 10.1242/dev.067322 22071105 PMC3222213

[bib199] Makhijani K. Alexander B. Rao D. Petraki S. Herboso L., 2017 Regulation of Drosophila hematopoietic sites by activin-beta from active sensory neurons. Nat. Commun. 8: 15990 10.1038/ncomms15990 28748922 PMC5537569

[bib200] Makki R. Meister M. Pennetier D. Ubeda J. M. Braun A., 2010 A short receptor downregulates JAK/STAT signalling to control the Drosophila cellular immune response. PLoS Biol. 8: e1000441 10.1371/journal.pbio.1000441 20689801 PMC2914635

[bib201] Manaka J. Kuraishi T. Shiratsuchi A. Nakai Y. Higashida H., 2004 Draper-mediated and phosphatidylserine-independent phagocytosis of apoptotic cells by Drosophila hemocytes/macrophages. J. Biol. Chem. 279: 48466–48476. 10.1074/jbc.M408597200 15342648

[bib202] Mandal L. Banerjee U. Hartenstein V., 2004 Evidence for a fruit fly hemangioblast and similarities between lymph-gland hematopoiesis in fruit fly and mammal aorta-gonadal-mesonephros mesoderm. Nat. Genet. 36: 1019–1023 (erratum: Nat. Genet. 36: 1126) 10.1038/ng1404 15286786

[bib203] Mandal L. Martinez-Agosto J. A. Evans C. J. Hartenstein V. Banerjee U., 2007 A Hedgehog- and Antennapedia-dependent niche maintains Drosophila haematopoietic precursors. Nature 446: 320–324. 10.1038/nature05585 17361183 PMC2807630

[bib204] Mansfield B. E. Dionne M. S. Schneider D. S. Freitag N. E., 2003 Exploration of host-pathogen interactions using Listeria monocytogenes and Drosophila melanogaster. Cell. Microbiol. 5: 901–911. 10.1046/j.1462-5822.2003.00329.x 14641175

[bib205] Márkus R. Kurucz E. Rus F. Andó I., 2005 Sterile wounding is a minimal and sufficient trigger for a cellular immune response in Drosophila melanogaster. Immunol. Lett. 101: 108–111. 10.1016/j.imlet.2005.03.021 15964636

[bib206] Márkus R. Laurinyecz B. Kurucz E. Honti V. Bajusz I., 2009 Sessile hemocytes as a hematopoietic compartment in Drosophila melanogaster. Proc. Natl. Acad. Sci. USA 106: 4805–4809. 10.1073/pnas.0801766106 19261847 PMC2660760

[bib207] Marshall C. J. Thrasher A. J., 2001 The embryonic origins of human haematopoiesis. Br. J. Haematol. 112: 838–850. 10.1046/j.1365-2141.2001.02537.x 11298579

[bib208] Marshall C. J. Kinnon C. Thrasher A. J., 2000 Polarized expression of bone morphogenetic protein-4 in the human aorta-gonad-mesonephros region. Blood 96: 1591–1593. 10942412

[bib209] Martinek N. Shahab J. Saathoff M. Ringuette M., 2008 Haemocyte-derived SPARC is required for collagen-IV-dependent stability of basal laminae in Drosophila embryos. J. Cell Sci. 121: 1671–1680. 10.1242/jcs.021931 18445681

[bib210] Matova N. Anderson K. V., 2006 Rel/NF-kappaB double mutants reveal that cellular immunity is central to Drosophila host defense. Proc. Natl. Acad. Sci. USA 103: 16424–16429. 10.1073/pnas.0605721103 17060622 PMC1637598

[bib211] Matsubayashi Y. Louani A. Dragu A. Sanchez-Sanchez B. J. Serna-Morales E., 2017 A moving source of matrix components is essential for de novo basement membrane formation. Curr. Biol. 27: 3526–3534.e4. 10.1016/j.cub.2017.10.001 29129537 PMC5714436

[bib212] McNally A. K. Anderson J. M., 2011 Macrophage fusion and multinucleated giant cells of inflammation. Adv. Exp. Med. Biol. 713: 97–111. 10.1007/978-94-007-0763-4_7 21432016

[bib213] Medvinsky A. Dzierzak E., 1996 Definitive hematopoiesis is autonomously initiated by the AGM region. Cell 86: 897–906. 10.1016/S0092-8674(00)80165-8 8808625

[bib214] Millar D. A. Ratcliffe N. A., 1989 The evolution of blood cells: facts and enigmas. Endeavour 13: 72–77. 10.1016/0160-9327(89)90005-7 2476300

[bib215] Miller, A., 1950 The internal anatomy and histology of the imago of Drosophila melanogaster, pp. 420–534 in *Biology of Drosophila*, edited by M. Demerec. Wiley-Interscience, New York.

[bib216] Milton C. C. Grusche F. A. Degoutin J. L. Yu E. Dai Q., 2014 The Hippo pathway regulates hematopoiesis in Drosophila melanogaster. Curr. Biol. 24: 2673–2680. 10.1016/j.cub.2014.10.031 25454587 PMC4269548

[bib217] Minakhina S. Steward R., 2006 Melanotic mutants in Drosophila: pathways and phenotypes. Genetics 174: 253–263. 10.1534/genetics.106.061978 16816412 PMC1569781

[bib218] Minakhina S. Steward R., 2010 Hematopoietic stem cells in Drosophila. Development 137: 27–31. 10.1242/dev.043943 20023157 PMC2796932

[bib219] Minakhina S. Tan W. Steward R., 2011 JAK/STAT and the GATA factor Pannier control hemocyte maturation and differentiation in Drosophila. Dev. Biol. 352: 308–316. 10.1016/j.ydbio.2011.01.035 21295568 PMC3065540

[bib220] Mondal B. C. Mukherjee T. Mandal L. Evans C. J. Sinenko S. A., 2011 Interaction between differentiating cell- and niche-derived signals in hematopoietic progenitor maintenance. Cell 147: 1589–1600. 10.1016/j.cell.2011.11.041 22196733 PMC4403793

[bib221] Mondal B. C. Shim J. Evans C. J. Banerjee U., 2014 Pvr expression regulators in equilibrium signal control and maintenance of Drosophila blood progenitors. Elife 3: e03626 10.7554/eLife.03626 25201876 PMC4185420

[bib222] Montagne J. Stewart M. J. Stocker H. Hafen E. Kozma S. C., 1999 Drosophila S6 kinase: a regulator of cell size. Science 285: 2126–2129. 10.1126/science.285.5436.2126 10497130

[bib223] Moreira S. Stramer B. Evans I. Wood W. Martin P., 2010 Prioritization of competing damage and developmental signals by migrating macrophages in the Drosophila embryo. Curr. Biol. 20: 464–470. 10.1016/j.cub.2010.01.047 20188558

[bib224] Morin-Poulard I. Sharma A. Louradour I. Vanzo N. Vincent A., 2016 Vascular control of the Drosophila haematopoietic microenvironment by Slit/Robo signalling. Nat. Commun. 7: 11634 10.1038/ncomms11634 27193394 PMC4874035

[bib225] Morrison S. J. Spradling A. C., 2008 Stem cells and niches: mechanisms that promote stem cell maintenance throughout life. Cell 132: 598–611. 10.1016/j.cell.2008.01.038 18295578 PMC4505728

[bib226] Mortimer N. T. Goecks J. Kacsoh B. Z. Mobley J. A. Bowersock G. J., 2013 Parasitoid wasp venom SERCA regulates Drosophila calcium levels and inhibits cellular immunity. Proc. Natl. Acad. Sci. USA 110: 9427–9432. 10.1073/pnas.1222351110 23690612 PMC3677475

[bib227] Morton D. B., 2004 Atypical soluble guanylyl cyclases in Drosophila can function as molecular oxygen sensors. J. Biol. Chem. 279: 50651–50653. 10.1074/jbc.C400461200 15485853

[bib228] Morton D. B. Stewart J. A. Langlais K. K. Clemens-Grisham R. A. Vermehren A., 2008 Synaptic transmission in neurons that express the Drosophila atypical soluble guanylyl cyclases, Gyc-89Da and Gyc-89Db, is necessary for the successful completion of larval and adult ecdysis. J. Exp. Biol. 211: 1645–1656. 10.1242/jeb.014472 18456892 PMC2424211

[bib229] Mukherjee T. Kim W. S. Mandal L. Banerjee U., 2011 Interaction between Notch and Hif-alpha in development and survival of Drosophila blood cells. Science 332: 1210–1213. 10.1126/science.1199643 21636775 PMC4412745

[bib230] Mukhopadhyay A. Das D. Inamdar M. S., 2003 Embryonic stem cell and tissue-specific expression of a novel conserved gene, asrij. Dev. Dyn. 227: 578–586. 10.1002/dvdy.10332 12889067

[bib231] Muralidharan S. Mandrekar P., 2013 Cellular stress response and innate immune signaling: integrating pathways in host defense and inflammation. J. Leukoc. Biol. 94: 1167–1184. 10.1189/jlb.0313153 23990626 PMC3828604

[bib232] Muratoglu S. Garratt B. Hyman K. Gajewski K. Schulz R. A., 2006 Regulation of Drosophila friend of GATA gene, u-shaped, during hematopoiesis: a direct role for serpent and lozenge. Dev. Biol. 296: 561–579. 10.1016/j.ydbio.2006.04.455 16730345

[bib233] Nam H. J. Jang I. H. Asano T. Lee W. J., 2008 Involvement of pro-phenoloxidase 3 in lamellocyte-mediated spontaneous melanization in Drosophila. Mol. Cells 26: 606–610. 18852525

[bib234] Nam H. J. Jang I. H. You H. Lee K. A. Lee W. J., 2012 Genetic evidence of a redox-dependent systemic wound response via Hayan protease-phenoloxidase system in Drosophila. EMBO J. 31: 1253–1265. 10.1038/emboj.2011.476 22227521 PMC3297987

[bib235] Nappi A. J. Vass E. Frey F. Carton Y., 1995 Superoxide anion generation in Drosophila during melanotic encapsulation of parasites. Eur. J. Cell Biol. 68: 450–456. 8690025

[bib236] Nässel D. R. Liu Y. Luo J., 2015 Insulin/IGF signaling and its regulation in Drosophila. Gen. Comp. Endocrinol. 221: 255–266. 10.1016/j.ygcen.2014.11.021 25616197

[bib237] Needham A. J. Kibart M. Crossley H. Ingham P. W. Foster S. J., 2004 Drosophila melanogaster as a model host for Staphylococcus aureus infection. Microbiology 150: 2347–2355. 10.1099/mic.0.27116-0 15256576

[bib238] Nehme N. T. Liegeois S. Kele B. Giammarinaro P. Pradel E., 2007 A model of bacterial intestinal infections in Drosophila melanogaster. PLoS Pathog. 3: e173 10.1371/journal.ppat.0030173 18039029 PMC2094306

[bib239] Nehme N. T. Quintin J. Cho J. H. Lee J. Lafarge M. C., 2011 Relative roles of the cellular and humoral responses in the Drosophila host defense against three gram-positive bacterial infections. PLoS One 6: e14743 10.1371/journal.pone.0014743 21390224 PMC3048390

[bib240] Nelson R. E. Fessler L. I. Takagi Y. Blumberg B. Keene D. R., 1994 Peroxidasin: a novel enzyme-matrix protein of Drosophila development. EMBO J. 13: 3438–3447. 10.1002/j.1460-2075.1994.tb06649.x 8062820 PMC395246

[bib241] Neyen C. Binggeli O. Roversi P. Bertin L. Sleiman M. B., 2015 The Black cells phenotype is caused by a point mutation in the Drosophila pro-phenoloxidase 1 gene that triggers melanization and hematopoietic defects. Dev. Comp. Immunol. 50: 166–174. 10.1016/j.dci.2014.12.011 25543001

[bib242] Nguyen H. T. Xu X., 1998 Drosophila mef2 expression during mesoderm development is controlled by a complex array of cis-acting regulatory modules. Dev. Biol. 204: 550–566. 10.1006/dbio.1998.9081 9882489

[bib243] Niethammer P. Grabher C. Look A. T. Mitchison T. J., 2009 A tissue-scale gradient of hydrogen peroxide mediates rapid wound detection in zebrafish. Nature 459: 996–999. 10.1038/nature08119 19494811 PMC2803098

[bib244] Nishikawa M. Tahara T. Hinohara A. Miyajima A. Nakahata T., 2001 Role of the microenvironment of the embryonic aorta-gonad-mesonephros region in hematopoiesis. Ann. N. Y. Acad. Sci. 938: 109–116. 10.1111/j.1749-6632.2001.tb03579.x 11458497

[bib245] Nonaka S. Nagaosa K. Mori T. Shiratsuchi A. Nakanishi Y., 2013 Integrin alphaPS3/betanu-mediated phagocytosis of apoptotic cells and bacteria in Drosophila. J. Biol. Chem. 288: 10374–10380. 10.1074/jbc.M113.451427 23426364 PMC3624420

[bib246] Okuda T. van Deursen J. Hiebert S. W. Grosveld G. Downing J. R., 1996 AML1, the target of multiple chromosomal translocations in human leukemia, is essential for normal fetal liver hematopoiesis. Cell 84: 321–330. 10.1016/S0092-8674(00)80986-1 8565077

[bib247] Oldham S., 2011 Obesity and nutrient sensing TOR pathway in flies and vertebrates: functional conservation of genetic mechanisms. Trends Endocrinol. Metab. 22: 45–52. 10.1016/j.tem.2010.11.002 21216618 PMC3035994

[bib248] Oldham S. Bohni R. Stocker H. Brogiolo W. Hafen E., 2000a Genetic control of size in Drosophila. Philos. Trans. R. Soc. Lond. B Biol. Sci. 355: 945–952. 10.1098/rstb.2000.0630 11128988 PMC1692799

[bib249] Oldham S. Montagne J. Radimerski T. Thomas G. Hafen E., 2000b Genetic and biochemical characterization of dTOR, the Drosophila homolog of the target of rapamycin. Genes Dev. 14: 2689–2694. 10.1101/gad.845700 11069885 PMC317036

[bib250] Olofsson B. Page D. T., 2005 Condensation of the central nervous system in embryonic Drosophila is inhibited by blocking hemocyte migration or neural activity. Dev. Biol. 279: 233–243. 10.1016/j.ydbio.2004.12.020 15708571

[bib251] Orkin S. H. Zon L. I., 2008 Hematopoiesis: an evolving paradigm for stem cell biology. Cell 132: 631–644. 10.1016/j.cell.2008.01.025 18295580 PMC2628169

[bib252] Osman D. Gobert V. Ponthan F. Heidenreich O. Haenlin M., 2009 A Drosophila model identifies calpains as modulators of the human leukemogenic fusion protein AML1-ETO. Proc. Natl. Acad. Sci. USA 106: 12043–12048. 10.1073/pnas.0902449106 19581587 PMC2715513

[bib253] Owusu-Ansah E. Banerjee U., 2009 Reactive oxygen species prime Drosophila haematopoietic progenitors for differentiation. Nature 461: 537–541. 10.1038/nature08313 19727075 PMC4380287

[bib254] Oyallon J. Vanzo N. Krzemien J. Morin-Poulard I. Vincent A., 2016 Two independent functions of Collier/Early B cell factor in the control of Drosophila blood cell homeostasis. PLoS One 11: e0148978 10.1371/journal.pone.0148978 26866694 PMC4750865

[bib255] Pagliarini R. A. Xu T., 2003 A genetic screen in Drosophila for metastatic behavior. Science 302: 1227–1231. 10.1126/science.1088474 14551319

[bib256] Parisi F. Stefanatos R. K. Strathdee K. Yu Y. Vidal M., 2014 Transformed epithelia trigger non-tissue-autonomous tumor suppressor response by adipocytes via activation of Toll and Eiger/TNF signaling. Cell Rep. 6: 855–867. 10.1016/j.celrep.2014.01.039 24582964

[bib257] Park M. Wu X. Golden K. Axelrod J. D. Bodmer R., 1996 The wingless signaling pathway is directly involved in Drosophila heart development. Dev. Biol. 177: 104–116. 10.1006/dbio.1996.0149 8660881

[bib258] Parsons B. Foley E., 2013 The Drosophila platelet-derived growth factor and vascular endothelial growth factor-receptor related (Pvr) protein ligands Pvf2 and Pvf3 control hemocyte viability and invasive migration. J. Biol. Chem. 288: 20173–20183. 10.1074/jbc.M113.483818 23737520 PMC3711285

[bib259] Pastor-Pareja J. C. Wu M. Xu T., 2008 An innate immune response of blood cells to tumors and tissue damage in Drosophila. Dis. Model. Mech. 1: 144–154, discussion 153. 10.1242/dmm.000950 19048077 PMC2562178

[bib260] Pelc R., 1986 The haemocytes and their classification in the larvae and pupae of Mamestra brassicae (L.) 1758 (Lepidoptera; Noctuidae). Can. J. Zool. 64: 2503–2508.

[bib261] Pennetier D. Oyallon J. Morin-Poulard I. Dejean S. Vincent A., 2012 Size control of the Drosophila hematopoietic niche by bone morphogenetic protein signaling reveals parallels with mammals. Proc. Natl. Acad. Sci. USA 109: 3389–3394. 10.1073/pnas.1109407109 22331866 PMC3295293

[bib262] Pérez E. Lindblad J. L. Bergmann A., 2017 Tumor-promoting function of apoptotic caspases by an amplification loop involving ROS, macrophages and JNK in Drosophila. Elife 6: e26747 10.7554/eLife.26747 28853394 PMC5779227

[bib263] Pham L. N. Dionne M. S. Shirasu-Hiza M. Schneider D. S., 2007 A specific primed immune response in Drosophila is dependent on phagocytes. PLoS Pathog. 3: e26 10.1371/journal.ppat.0030026 17352533 PMC1817657

[bib264] Phillips R. L. Ernst R. E. Brunk B. Ivanova N. Mahan M. A., 2000 The genetic program of hematopoietic stem cells. Science 288: 1635–1640. 10.1126/science.288.5471.1635 10834841

[bib265] Pila E. A. Sullivan J. T. Wu X. Z. Fang J. Rudko S. P., 2016 Haematopoiesis in molluscs: a review of haemocyte development and function in gastropods, cephalopods and bivalves. Dev. Comp. Immunol. 58: 119–128. 10.1016/j.dci.2015.11.010 26592965 PMC4775334

[bib266] Qiu P. Pan P. C. Govind S., 1998 A role for the Drosophila Toll/Cactus pathway in larval hematopoiesis. Development 125: 1909–1920. 9550723 10.1242/dev.125.10.1909

[bib267] Rämet M. Lanot R. Zachary D. Manfruelli P., 2002 JNK signaling pathway is required for efficient wound healing in Drosophila. Dev. Biol. 241: 145–156. 10.1006/dbio.2001.0502 11784101

[bib268] Ratcliffe N. A. Rowley A. F., 1981 Invertebrate Blood Cells. Academic Press, London.

[bib269] Ratheesh A. Belyaeva V. Siekhaus D. E., 2015 Drosophila immune cell migration and adhesion during embryonic development and larval immune responses. Curr. Opin. Cell Biol. 36: 71–79. 10.1016/j.ceb.2015.07.003 26210104

[bib270] Razzell W. Evans I. R. Martin P. Wood W., 2013 Calcium flashes orchestrate the wound inflammatory response through DUOX activation and hydrogen peroxide release. Curr. Biol. 23: 424–429. 10.1016/j.cub.2013.01.058 23394834 PMC3629559

[bib271] Regan J. C. Brandao A. S. Leitao A. B. Mantas Dias A. R. Sucena E., 2013 Steroid hormone signaling is essential to regulate innate immune cells and fight bacterial infection in Drosophila. PLoS Pathog. 9: e1003720 10.1371/journal.ppat.1003720 24204269 PMC3812043

[bib272] Rehorn K. P. Thelen H. Michelson A. M. Reuter R., 1996 A molecular aspect of hematopoiesis and endoderm development common to vertebrates and Drosophila. Development 122: 4023–4031. 9012522 10.1242/dev.122.12.4023

[bib273] Reineke A. Asgari S. Ma G. Beck M. Schmidt O., 2002 Sequence analysis and expression of a virus-like particle protein, VLP2, from the parasitic wasp Venturia canescens. Insect Mol. Biol. 11: 233–239. 10.1046/j.1365-2583.2002.00330.x 12000642

[bib274] Reitman Z. J. Sinenko S. A. Spana E. P. Yan H., 2015 Genetic dissection of leukemia-associated IDH1 and IDH2 mutants and D-2-hydroxyglutarate in Drosophila. Blood 125: 336–345. 10.1182/blood-2014-05-577940 25398939 PMC4287640

[bib275] Ribeiro C. Brehelin M., 2006 Insect haemocytes: what type of cell is that? J. Insect Physiol. 52: 417–429. 10.1016/j.jinsphys.2006.01.005 16527302

[bib276] Riechmann V. Irion U. Wilson R. Grosskortenhaus R. Leptin M., 1997 Control of cell fates and segmentation in the Drosophila mesoderm. Development 124: 2915–2922. 9247334 10.1242/dev.124.15.2915

[bib277] Rizki M. T. M., 1957 Alterations in the haemocyte population of Drosophila melanogaster. J. Morphol. 100: 437–458. 10.1002/jmor.1051000303

[bib278] Rizki R. M. Rizki T. M., 1974 Basement membrane abnormalities in melanotic tumor formation of Drosophila. Experientia 30: 543–546. 10.1007/BF01926343 4208981

[bib279] Rizki R. M. Rizki T. M., 1979 Cell interactions in the differentiation of a melanotic tumor in Drosophila. Differentiation 12: 167–178. 10.1111/j.1432-0436.1979.tb01002.x 111992

[bib280] Rizki R. M. Rizki T. M., 1984 Selective destruction of a host blood cell type by a parasitoid wasp. Proc. Natl. Acad. Sci. USA 81: 6154–6158. 10.1073/pnas.81.19.6154 6435126 PMC391878

[bib281] Rizki R. M. Rizki T. M., 1991 Effects of lamellolysin from a parasitoid wasp on Drosophila blood cells in vitro. J. Exp. Zool. 257: 236–244. 10.1002/jez.1402570214 1899269

[bib282] Rizki T. M., 1978 The circulatory system and associated cells and tissues, pp. 397–452 in The Genetics and Biology of Drosophila, edited by Ashburner M. Wright T. R. F. Academic Press, London.

[bib283] Rizki T. M. Rizki R. M., 1983 Blood cell surface changes in Drosophila mutants with melanotic tumors. Science 220: 73–75. 10.1126/science.6402819 6402819

[bib284] Rizki T. M. Rizki R. M., 1992 Lamellocyte differentiation in Drosophila larvae parasitized by Leptopilina. Dev. Comp. Immunol. 16: 103–110. 10.1016/0145-305X(92)90011-Z 1499832

[bib285] Rizki T. M. Rizki R. M. Grell E. H., 1980 A mutant affecting the crystal cells inDrosophila melanogaster. Wilehm Roux Arch Dev Biol 188: 91–99. 10.1007/BF00848799 28304971

[bib286] Rodriguez A. Zhou Z. Tang M. L. Meller S. Chen J., 1996 Identification of immune system and response genes, and novel mutations causing melanotic tumor formation in Drosophila melanogaster. Genetics 143: 929–940. 8725239 10.1093/genetics/143.2.929 PMC1207349

[bib287] Roman G. He J. Davis R. L., 2000 kurtz, a novel nonvisual arrestin, is an essential neural gene in Drosophila. Genetics 155: 1281–1295. 10880488 10.1093/genetics/155.3.1281 PMC1461172

[bib288] Rovira I. I. Finkel T., 2008 Reactive oxygen species as signaling molecules, pp. 293–307 in Oxidative Stress in Aging: From Model Systems to Human Diseases, edited by Miwa S. Beckman K. B. Muller F. L. Humana Press, Totowa, NJ 10.1007/978-1-59745-420-9_16

[bib289] Rugendorff A. Younossi-Hartenstein A. Hartenstein V., 1994 Embryonic origin and differentiation of the Drosophila heart. Rouxs Arch. Dev. Biol. 203: 266–280. 10.1007/BF00360522 28305624

[bib290] Russo J. Dupas S. Frey F. Carton Y. Brehelin M., 1996 Insect immunity: early events in the encapsulation process of parasitoid (Leptopilina boulardi) eggs in resistant and susceptible strains of Drosophila. Parasitology 112: 135–142. 10.1017/S0031182000065173 8587797

[bib291] Sam S. Leise W. Hoshizaki D. K., 1996 The serpent gene is necessary for progression through the early stages of fat-body development. Mech. Dev. 60: 197–205. 10.1016/S0925-4773(96)00615-6 9025072

[bib292] Sánchez-Sánchez B. J. Urbano J. M. Comber K. Dragu A. Wood W., 2017 Drosophila embryonic hemocytes produce laminins to strengthen migratory response. Cell Rep. 21: 1461–1470. 10.1016/j.celrep.2017.10.047 29117553 PMC5695906

[bib293] Scherfer C. Karlsson C. Loseva O. Bidla G. Goto A., 2004 Isolation and characterization of hemolymph clotting factors in Drosophila melanogaster by a pullout method. Curr. Biol. 14: 625–629. 10.1016/j.cub.2004.03.030 15062105

[bib294] Schieber M. Chandel N. S., 2014 ROS function in redox signaling and oxidative stress. Curr. Biol. 24: R453–R462. 10.1016/j.cub.2014.03.034 24845678 PMC4055301

[bib295] Schmid M. R. Anderl I. Vesala L. Vanha-aho L. M. Deng X. J., 2014 Control of Drosophila blood cell activation via Toll signaling in the fat body. PLoS One 9: e102568 10.1371/journal.pone.0102568 25102059 PMC4125153

[bib296] Schmid M. R. Anderl I. Vo H. T. Valanne S. Yang H., 2016 Genetic screen in Drosophila larvae links ird1 function to toll signaling in the fat body and hemocyte motility. PLoS One 11: e0159473 10.1371/journal.pone.0159473 27467079 PMC4965076

[bib297] Schneider D. S. Ayres J. S. Brandt S. M. Costa A. Dionne M. S., 2007 Drosophila eiger mutants are sensitive to extracellular pathogens. PLoS Pathog. 3: e41 10.1371/journal.ppat.0030041 17381241 PMC1829408

[bib298] Sears H. C. Kennedy C. J. Garrity P. A., 2003 Macrophage-mediated corpse engulfment is required for normal Drosophila CNS morphogenesis. Development 130: 3557–3565. 10.1242/dev.00586 12810602

[bib299] Senger K. Harris K. Levine M., 2006 GATA factors participate in tissue-specific immune responses in Drosophila larvae. Proc. Natl. Acad. Sci. USA 103: 15957–15962. 10.1073/pnas.0607608103 17032752 PMC1635109

[bib300] Shia A. K. Glittenberg M. Thompson G. Weber A. N. Reichhart J. M., 2009 Toll-dependent antimicrobial responses in Drosophila larval fat body require Spatzle secreted by haemocytes. J. Cell Sci. 122: 4505–4515. 10.1242/jcs.049155 19934223 PMC2787462

[bib301] Shibata T. Hadano J. Kawasaki D. Dong X. Kawabata S. I., 2017 Drosophila TG-A transglutaminase is secreted via an unconventional Golgi-independent mechanism involving exosomes and two types of fatty acylations. J. Biol. Chem. 292: 10723–10734. 10.1074/jbc.M117.779710 28476891 PMC5481576

[bib302] Shim J. Mukherjee T. Banerjee U., 2012 Direct sensing of systemic and nutritional signals by haematopoietic progenitors in Drosophila. Nat. Cell Biol. 14: 394–400. 10.1038/ncb2453 22407365 PMC4342111

[bib303] Shim J. Mukherjee T. Mondal B. C. Liu T. Young G. C., 2013 Olfactory control of blood progenitor maintenance. Cell 155: 1141–1153. 10.1016/j.cell.2013.10.032 24267893 PMC3865989

[bib304] Shishido E. Ono N. Kojima T. Saigo K., 1997 Requirements of DFR1/Heartless, a mesoderm-specific Drosophila FGF-receptor, for the formation of heart, visceral and somatic muscles, and ensheathing of longitudinal axon tracts in CNS. Development 124: 2119–2128. 9187139 10.1242/dev.124.11.2119

[bib305] Shrestha R. Gateff E., 1982 Ultrastructure and cytochemistry of the cell types in the larval hematopoietic organs and hemolymph of Drosophila melanogaster. Dev. Growth Differ. 24: 65–82. 10.1111/j.1440-169X.1982.00065.x 37281804

[bib306] Simpson T. L., 1984 The Cell Biology of Sponges. Springer-Verlag, New York 10.1007/978-1-4612-5214-6

[bib307] Sinenko S. A. Mathey-Prevot B., 2004 Increased expression of Drosophila tetraspanin, Tsp68C, suppresses the abnormal proliferation of ytr-deficient and Ras/Raf-activated hemocytes. Oncogene 23: 9120–9128. 10.1038/sj.onc.1208156 15480416

[bib308] Sinenko S. A. Mandal L. Martinez-Agosto J. A. Banerjee U., 2009 Dual role of wingless signaling in stem-like hematopoietic precursor maintenance in Drosophila. Dev. Cell 16: 756–763. 10.1016/j.devcel.2009.03.003 19460351 PMC2718753

[bib309] Sinenko S. A. Hung T. Moroz T. Tran Q. M. Sidhu S., 2010 Genetic manipulation of AML1-ETO-induced expansion of hematopoietic precursors in a Drosophila model. Blood 116: 4612–4620. 10.1182/blood-2010-03-276998 20688956 PMC2996118

[bib310] Sinenko S. A. Shim J. Banerjee U., 2011 Oxidative stress in the haematopoietic niche regulates the cellular immune response in Drosophila. EMBO Rep. 13: 83–89. 10.1038/embor.2011.223 22134547 PMC3246251

[bib311] Sinha A. Khadilkar R. J. Roychowdhury S. V. K. Sinha A. Inamdar M. S., 2013 Conserved regulation of the Jak/STAT pathway by the endosomal protein asrij maintains stem cell potency. Cell Rep. 4: 649–658. 10.1016/j.celrep.2013.07.029 23972987 PMC4673900

[bib312] Small C. Ramroop J. Otazo M. Huang L. H. Saleque S., 2014 An unexpected link between notch signaling and ROS in restricting the differentiation of hematopoietic progenitors in Drosophila. Genetics 197: 471–483. 10.1534/genetics.113.159210 24318532 PMC4063908

[bib313] Sorrentino R. P. Carton Y. Govind S., 2002 Cellular immune response to parasite infection in the Drosophila lymph gland is developmentally regulated. Dev. Biol. 243: 65–80. 10.1006/dbio.2001.0542 11846478

[bib314] Sorrentino R. P. Melk J. P. Govind S., 2004 Genetic analysis of contributions of dorsal group and JAK-Stat92E pathway genes to larval hemocyte concentration and the egg encapsulation response in Drosophila. Genetics 166: 1343–1356. 10.1534/genetics.166.3.1343 15082553 PMC1470785

[bib315] Sorrentino R. P. Tokusumi T. Schulz R. A., 2007 The Friend of GATA protein U-shaped functions as a hematopoietic tumor suppressor in Drosophila. Dev. Biol. 311: 311–323. 10.1016/j.ydbio.2007.08.011 17936744

[bib316] Spahn P. Huelsmann S. Rehorn K. P. Mischke S. Mayer M., 2014 Multiple regulatory safeguards confine the expression of the GATA factor Serpent to the hemocyte primordium within the Drosophila mesoderm. Dev. Biol. 386: 272–279. 10.1016/j.ydbio.2013.12.012 24360907

[bib317] Stofanko M. Kwon S. Y. Badenhorst P., 2008 A misexpression screen to identify regulators of Drosophila larval hemocyte development. Genetics 180: 253–267. 10.1534/genetics.108.089094 18757933 PMC2535679

[bib318] Stofanko M. Kwon S. Y. Badenhorst P., 2010 Lineage tracing of lamellocytes demonstrates Drosophila macrophage plasticity. PLoS One 5: e14051 10.1371/journal.pone.0014051 21124962 PMC2988793

[bib319] Stramer B. Wood W. Galko M. J. Redd M. J. Jacinto A., 2005 Live imaging of wound inflammation in Drosophila embryos reveals key roles for small GTPases during in vivo cell migration. J. Cell Biol. 168: 567–573. 10.1083/jcb.200405120 15699212 PMC2171743

[bib320] Stroschein-Stevenson S. L. Foley E. O’Farrell P. H. Johnson A. D., 2006 Identification of Drosophila gene products required for phagocytosis of Candida albicans. PLoS Biol. 4: e4 10.1371/journal.pbio.0040004 16336044 PMC1310651

[bib321] Tang H. Kambris Z. Lemaitre B. Hashimoto C., 2006 Two proteases defining a melanization cascade in the immune system of Drosophila. J. Biol. Chem. 281: 28097–28104. 10.1074/jbc.M601642200 16861233

[bib322] Taylor M. V. Beatty K. E. Hunter H. K. Baylies M. K., 1995 Drosophila MEF2 is regulated by twist and is expressed in both the primordia and differentiated cells of the embryonic somatic, visceral and heart musculature. Mech. Dev. 50: 29–41. 10.1016/0925-4773(94)00323-F 7605749

[bib323] Teleman A. A. Hietakangas V. Sayadian A. C. Cohen S. M., 2008 Nutritional control of protein biosynthetic capacity by insulin via Myc in Drosophila. Cell Metab. 7: 21–32. 10.1016/j.cmet.2007.11.010 18177722

[bib324] Tepass U. Fessler L. I. Aziz A. Hartenstein V., 1994 Embryonic origin of hemocytes and their relationship to cell death in Drosophila. Development 120: 1829–1837. 7924990 10.1242/dev.120.7.1829

[bib325] Terriente-Felix A. Li J. Collins S. Mulligan A. Reekie I., 2013 Notch cooperates with Lozenge/Runx to lock haemocytes into a differentiation programme. Development 140: 926–937. 10.1242/dev.086785 23325760 PMC3557782

[bib326] Thummel C. S., 2001 Molecular mechanisms of developmental timing in C. elegans and Drosophila. Dev. Cell 1: 453–465. 10.1016/S1534-5807(01)00060-0 11703937

[bib327] Tirouvanziam R. Davidson C. J. Lipsick J. S. Herzenberg L. A., 2004 Fluorescence-activated cell sorting (FACS) of Drosophila hemocytes reveals important functional similarities to mammalian leukocytes. Proc. Natl. Acad. Sci. USA 101: 2912–2917. 10.1073/pnas.0308734101 14976247 PMC365719

[bib328] Tokusumi T. Shoue D. A. Tokusumi Y. Stoller J. R. Schulz R. A., 2009a New hemocyte-specific enhancer-reporter transgenes for the analysis of hematopoiesis in Drosophila. Genesis 47: 771–774. 10.1002/dvg.20561 19830816

[bib329] Tokusumi T. Sorrentino R. P. Russell M. Ferrarese R. Govind S., 2009b Characterization of a lamellocyte transcriptional enhancer located within the misshapen gene of Drosophila melanogaster. PLoS One 4: e6429 10.1371/journal.pone.0006429 19641625 PMC2713827

[bib330] Tokusumi T. Tokusumi Y. Brahier M. S. Lam V. Stoller-Conrad J. R., 2017 Screening and analysis of Janelia flyLight project enhancer-Gal4 strains identifies multiple gene enhancers active during hematopoiesis in normal and Wasp-challenged Drosophila larvae. G3 (Bethesda) 7: 437–448. 10.1534/g3.116.034439 27913635 PMC5295592

[bib331] Tokusumi Y. Tokusumi T. Shoue D. A. Schulz R. A., 2012 Gene regulatory networks controlling hematopoietic progenitor niche cell production and differentiation in the Drosophila lymph gland. PLoS One 7: e41604 [corrigenda: PLoS One 8 (2013)]. 10.1371/journal.pone.0041604 22911822 PMC3404040

[bib332] Tothova Z. Kollipara R. Huntly B. J. Lee B. H. Castrillon D. H., 2007 FoxOs are critical mediators of hematopoietic stem cell resistance to physiologic oxidative stress. Cell 128: 325–339. 10.1016/j.cell.2007.01.003 17254970

[bib333] Tung T. T. Nagaosa K. Fujita Y. Kita A. Mori H., 2013 Phosphatidylserine recognition and induction of apoptotic cell clearance by Drosophila engulfment receptor Draper. J. Biochem. 153: 483–491. 10.1093/jb/mvt014 23420848

[bib334] Ulvila J. Vanha-Aho L. M. Ramet M., 2011 Drosophila phagocytosis - still many unknowns under the surface. APMIS 119: 651–662. 10.1111/j.1600-0463.2011.02792.x 21917002

[bib335] Van De Bor V. Zimniak G. Papone L. Cerezo D. Malbouyres M., 2015 Companion blood cells control ovarian stem cell niche microenvironment and homeostasis. Cell Rep. 13: 546–560. 10.1016/j.celrep.2015.09.008 26456819

[bib336] Vanha-Aho L. M. Anderl I. Vesala L. Hultmark D. Valanne S., 2015 Edin expression in the fat body is required in the defense against parasitic wasps in Drosophila melanogaster. PLoS Pathog. 11: e1004895 10.1371/journal.ppat.1004895 25965263 PMC4429011

[bib337] Vermehren-Schmaedick A. Ainsley J. A. Johnson W. A. Davies S. A. Morton D. B., 2010 Behavioral responses to hypoxia in Drosophila larvae are mediated by atypical soluble guanylyl cyclases. Genetics 186: 183–196. 10.1534/genetics.110.118166 20592263 PMC2940286

[bib338] Vetvicka V. Šíma P., 2009 Origins and functions of annelide immune cells: the concise survey. Inv. Surv. J. 6: 138–143.

[bib339] Vodovar N. Vinals M. Liehl P. Basset A. Degrouard J., 2005 Drosophila host defense after oral infection by an entomopathogenic Pseudomonas species. Proc. Natl. Acad. Sci. USA 102: 11414–11419. 10.1073/pnas.0502240102 16061818 PMC1183552

[bib340] Waltzer L. Bataille L. Peyrefitte S. Haenlin M., 2002 Two isoforms of Serpent containing either one or two GATA zinc fingers have different roles in Drosophila haematopoiesis. EMBO J. 21: 5477–5486. 10.1093/emboj/cdf545 12374748 PMC129077

[bib341] Waltzer L. Ferjoux G. Bataille L. Haenlin M., 2003 Cooperation between the GATA and RUNX factors Serpent and Lozenge during Drosophila hematopoiesis. EMBO J. 22: 6516–6525. 10.1093/emboj/cdg622 14657024 PMC291817

[bib342] Wang Z. Wilhelmsson C. Hyrsl P. Loof T. G. Dobes P., 2010 Pathogen entrapment by transglutaminase–a conserved early innate immune mechanism. PLoS Pathog. 6: e1000763 10.1371/journal.ppat.1000763 20169185 PMC2820530

[bib343] Ward E. J. Skeath J. B., 2000 Characterization of a novel subset of cardiac cells and their progenitors in the Drosophila embryo. Development 127: 4959–4969. 11044409 10.1242/dev.127.22.4959

[bib344] Wasserman S. A., 2004 Nature’s fortress against infection. Nat. Immunol. 5: 474–475. 10.1038/ni0504-474 15116113

[bib345] Watson F. L. Puttmann-Holgado R. Thomas F. Lamar D. L. Hughes M., 2005 Extensive diversity of Ig-superfamily proteins in the immune system of insects. Science 309: 1874–1878. 10.1126/science.1116887 16109846

[bib346] Watson K. L. Johnson T. K. Denell R. E., 1991 Lethal(1) aberrant immune response mutations leading to melanotic tumor formation in Drosophila melanogaster. Dev. Genet. 12: 173–187. 10.1002/dvg.1020120302 1907895

[bib347] Weavers H. Prieto-Sanchez S. Grawe F. Garcia-Lopez A. Artero R., 2009 The insect nephrocyte is a podocyte-like cell with a filtration slit diaphragm. Nature 457: 322–326. 10.1038/nature07526 18971929 PMC2687078

[bib348] Weavers H. Evans I. R. Martin P. Wood W., 2016a Corpse engulfment generates a molecular memory that primes the macrophage inflammatory response. Cell 165: 1658–1671. 10.1016/j.cell.2016.04.049 27212238 PMC4912690

[bib349] Weavers H. Liepe J. Sim A. Wood W. Martin P., 2016b Systems analysis of the dynamic inflammatory response to tissue damage reveals spatiotemporal properties of the wound attractant gradient. Curr. Biol. 26: 1975–1989. 10.1016/j.cub.2016.06.012 27426513 PMC4985561

[bib350] Weber G. F. Bjerke M. A. DeSimone D. W., 2011 Integrins and cadherins join forces to form adhesive networks. J. Cell Sci. 124: 1183–1193. 10.1242/jcs.064618 21444749 PMC3115772

[bib351] Weismann A., 1864 Die nachembryonale Entwicklung der Musciden nach Beobachtungen an Musca vomitoria und Sar- cophaga carnaria. Z. Wiss. Zool. 14: 187–336.

[bib352] Weissman I. L. Anderson D. J. Gage F., 2001 Stem and progenitor cells: origins, phenotypes, lineage commitments, and transdifferentiations. Annu. Rev. Cell Dev. Biol. 17: 387–403. 10.1146/annurev.cellbio.17.1.387 11687494

[bib353] Wertheim B. Kraaijeveld A. R. Schuster E. Blanc E. Hopkins M., 2005 Genome-wide gene expression in response to parasitoid attack in Drosophila. Genome Biol. 6: R94 10.1186/gb-2005-6-11-r94 16277749 PMC1297650

[bib354] Williams M. J., 2009 The Drosophila cell adhesion molecule Neuroglian regulates Lissencephaly-1 localisation in circulating immunosurveillance cells. BMC Immunol. 10: 17 10.1186/1471-2172-10-17 19320973 PMC2667480

[bib355] Williams M. J. Wiklund M. L. Wikman S. Hultmark D., 2006 Rac1 signalling in the Drosophila larval cellular immune response. J. Cell Sci. 119: 2015–2024. 10.1242/jcs.02920 16621891

[bib356] Wood W. Martin P., 2017 Macrophage functions in tissue patterning and disease: new insights from the fly. Dev. Cell 40: 221–233. 10.1016/j.devcel.2017.01.001 28171746 PMC5300050

[bib357] Wood W. Jacinto A. Grose R. Woolner S. Gale J., 2002 Wound healing recapitulates morphogenesis in Drosophila embryos. Nat. Cell Biol. 4: 907–912. 10.1038/ncb875 12402048

[bib358] Wood W. Faria C. Jacinto A., 2006 Distinct mechanisms regulate hemocyte chemotaxis during development and wound healing in Drosophila melanogaster. J. Cell Biol. 173: 405–416. 10.1083/jcb.200508161 16651377 PMC2063841

[bib359] Woodcock K. J. Kierdorf K. Pouchelon C. A. Vivancos V. Dionne M. S., 2015 Macrophage-derived upd3 cytokine causes impaired glucose homeostasis and reduced lifespan in Drosophila fed a lipid-rich diet. Immunity 42: 133–144. 10.1016/j.immuni.2014.12.023 25601202 PMC4304720

[bib360] Wu S. C. Cao Z. S. Chang K. M. Juang J. L., 2017 Intestinal microbial dysbiosis aggravates the progression of Alzheimer’s disease in Drosophila. Nat. Commun. 8: 24 10.1038/s41467-017-00040-6 28634323 PMC5478647

[bib361] Xavier M. J. Williams M. J., 2011 The Rho-family GTPase Rac1 regulates integrin localization in Drosophila immunosurveillance cells. PLoS One 6: e19504 10.1371/journal.pone.0019504 21603603 PMC3095607

[bib362] Xiao H. Wang H. Silva E. A. Thompson J. Guillou A., 2015 The Pallbearer E3 ligase promotes actin remodeling via RAC in efferocytosis by degrading the ribosomal protein S6. Dev. Cell 32: 19–30. 10.1016/j.devcel.2014.11.015 25533207 PMC4293263

[bib363] Yang H. Hultmark D., 2017 Drosophila muscles regulate the immune response against wasp infection via carbohydrate metabolism. Sci. Rep. 7: 15713 10.1038/s41598-017-15940-2 29146985 PMC5691183

[bib364] Yang H. Kronhamn J. Ekstrom J. O. Korkut G. G. Hultmark D., 2015 JAK/STAT signaling in Drosophila muscles controls the cellular immune response against parasitoid infection. EMBO Rep. 16: 1664–1672. 10.15252/embr.201540277 26412855 PMC4687419

[bib365] Yano T. Mita S. Ohmori H. Oshima Y. Fujimoto Y., 2008 Autophagic control of listeria through intracellular innate immune recognition in drosophila. Nat. Immunol. 9: 908–916. 10.1038/ni.1634 18604211 PMC2562576

[bib366] Yin Z. Xu X. L. Frasch M., 1997 Regulation of the twist target gene tinman by modular cis-regulatory elements during early mesoderm development. Development 124: 4971–4982. 9362473 10.1242/dev.124.24.4971

[bib367] Yokomizo T. Ogawa M. Osato M. Kanno T. Yoshida H., 2001 Requirement of Runx1/AML1/PEBP2alphaB for the generation of haematopoietic cells from endothelial cells. Genes Cells 6: 13–23. 10.1046/j.1365-2443.2001.00393.x 11168593

[bib368] Yoo S. K. Starnes T. W. Deng Q. Huttenlocher A., 2011 Lyn is a redox sensor that mediates leukocyte wound attraction in vivo. Nature 480: 109–112. 10.1038/nature10632 22101434 PMC3228893

[bib369] Yu S. Zhang G. Jin L. H., 2018 A high-sugar diet affects cellular and humoral immune responses in Drosophila. Exp. Cell Res. 368: 215–224. 10.1016/j.yexcr.2018.04.032 29727694

[bib370] Zaffran S. Xu X. Lo P. C. Lee H. H. Frasch M., 2002 Cardiogenesis in the Drosophila model: control mechanisms during early induction and diversification of cardiac progenitors. Cold Spring Harb. Symp. Quant. Biol. 67: 1–12. 10.1101/sqb.2002.67.1 12858517

[bib371] Zettervall C. J. Anderl I. Williams M. J. Palmer R. Kurucz E., 2004 A directed screen for genes involved in Drosophila blood cell activation. Proc. Natl. Acad. Sci. USA 101: 14192–14197. 10.1073/pnas.0403789101 15381778 PMC521135

[bib372] Zhang C. U. Cadigan K. M., 2017 The matrix protein Tiggrin regulates plasmatocyte maturation in Drosophila larva. Development 144: 2415–2427. 10.1242/dev.149641 28526755 PMC5536868

[bib373] Zhang C. U. Blauwkamp T. A. Burby P. E. Cadigan K. M., 2014 Wnt-mediated repression via bipartite DNA recognition by TCF in the Drosophila hematopoietic system. PLoS Genet. 10: e1004509 10.1371/journal.pgen.1004509 25144371 PMC4140642

[bib374] Zhang G. Hao Y. Jin L. H., 2016 Overexpression of jumu induces melanotic nodules by activating Toll signaling in Drosophila. Insect Biochem. Mol. Biol. 77: 31–38. 10.1016/j.ibmb.2016.08.002 27507244

[bib375] Zhang H. Stallock J. P. Ng J. C. Reinhard C. Neufeld T. P., 2000 Regulation of cellular growth by the Drosophila target of rapamycin dTOR. Genes Dev. 14: 2712–2724. 10.1101/gad.835000 11069888 PMC317034

[bib376] Zhou Z. Rodriguez A. Wu C. Y. Kimbrell D. A., 2001 Drosophila cellular immune system: dorothy encodes a UDP glycosyltransferase. Adv. Exp. Med. Biol. 484: 251–263. 10.1007/978-1-4615-1291-2_24 11418991

[bib377] Zmojdzian M. de Joussineau S. Da Ponte J. P. Jagla K., 2018 Distinct subsets of Eve-positive pericardial cells stabilise cardiac outflow and contribute to Hox gene-triggered heart morphogenesis in Drosophila. Development 145: dev158717. 10.1242/dev.158717 PMC5825839 29247145

[bib378] Zurovec M. Dolezal T. Gazi M. Pavlova E. Bryant P. J., 2002 Adenosine deaminase-related growth factors stimulate cell proliferation in Drosophila by depleting extracellular adenosine. Proc. Natl. Acad. Sci. USA 99: 4403–4408. 10.1073/pnas.062059699 11904370 PMC123660

